# Analysis of GRK2 aggregation in the pathology of Alzheimer disease in animal models

**DOI:** 10.1016/j.xcrm.2026.102707

**Published:** 2026-03-26

**Authors:** Joshua Abd Alla, Alexander Perhal, Xuebin Fu, Andreas Langer, Yasser el Faramawy, Ursula Quitterer

**Affiliations:** 1 Molecular Pharmacology, ETH Zurich, 8057 Zurich, Switzerland; 2 Department of Pharmaceutical Sciences, University of Vienna, 1090 Vienna, Austria; 3 Department of Pediatrics, Northwestern University Feinberg School of Medicine, Chicago, IL 60611, USA; 4 Medical Research Center (MRC), Ain Shams University Hospitals, Cairo 11566, Egypt; 5 Institute of Pharmacology and Toxicology, University of Zurich, 8057 Zurich, Switzerland

**Keywords:** Alzheimer disease, GRK2, ARRB1, TOMM6, senescence, proteasome, molecular docking

## Abstract

The G-protein-coupled receptor kinase 2 (GRK2) exerts essential functions in cell growth and survival. Searching for a connection between GRK2 and the neurodegenerative Alzheimer disease (AD), we find increased aggregated serine-670-phosphorylated GRK2 (phospho-S670-GRK2) in brains of AD mice and patients with dementia likely due to AD. Harmful phospho-S670-GRK2 aggregation is induced by two hallmark proteins of AD: beta-amyloid and the neurofibrillary-tangle-inducing, TAU-P301L. Aggregated phospho-S670-GRK2 triggers aggregation of TOMM6 (translocase of outer mitochondrial membrane 6), promotes mitochondrial dysfunction, and enhances beta-amyloid. Transgenic expression of inactive GRK2-K220R or a GRK-inhibitory peptide proves that neuropathological features are caused by GRK2 inactivation. Restoration of TOMM6 by neuron-specific TOMM6 expression reduces beta-amyloid plaques but enhances soluble beta-amyloid and increases mortality. In contrast, reconstitution of monomeric GRK2 and proteasomal phospho-S670-GRK2 degradation by small molecules counteracts neuropathological AD features, prevents neuronal loss, and improves survival. Thus, targeting of pathological GRK2 aggregation slows aging-induced neurodegeneration.

## Highlights

• Alzheimer brains have increased phospho-S670-GRK2 protein aggregates
• The AD hallmarks, beta-amyloid and hyperphosphorylated Tau, induce GRK2 aggregation
• Aggregated phospho-S670-GRK2 enhances beta-amyloid and mitochondrial dysfunction
• Restoration of functional GRK2 monomers by small molecules retards AD symptoms

## Graphical abstract

**Figure:**
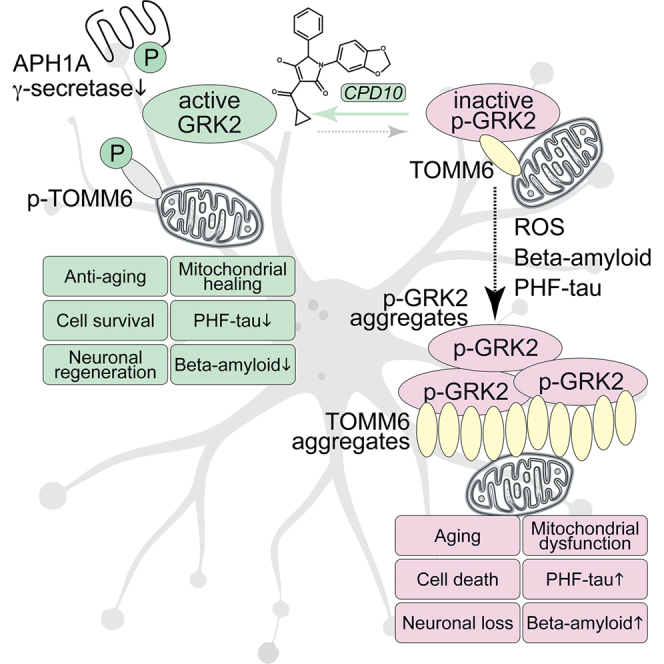

## Teaser

Abd Alla et al. find increased dysfunctional protein aggregates of the G-protein-coupled receptor kinase 2 (GRK2) in Alzheimer brains, causing mitochondrial dysfunction and enhanced beta-amyloid accumulation. Prevention of dysfunctional GRK2 aggregation by transgenic techniques and small molecules retards neurodegeneration, reduces neuronal loss, and prolongs survival.

## Introduction

Alzheimer disease (AD) is the most frequent form of dementia without cure.^1^^,^^2^ Searching for a pharmacological target, we focused on the G-protein-coupled receptor kinase 2 (GRK2).^3^ GRK2 exerts a wide array of neuroprotective activities,^4^^,^^5^^,^^6^^,^^7^ but the impact of GRK2 on AD pathogenesis is not understood.^8^^,^^9^^,^^10^^,^^11^^,^^12^^,^^13^^,^^14^^,^^15^ In AD brains, GRK2 was localized close to damaged mitochondria, which is abnormal.^8^^,^^10^^,^^14^ Mitochondrial GRK2 is triggered by phosphorylation at serine-670, in failing hearts, and exerts mitochondrial dysfunction and pro-death signaling.^16^^,^^17^ In contrast, a fine-tuned equilibrium between phospho-S670-GRK2 degradation and non-phospho-GRK2 is required for cell-cycle progression and cell survival.^18^^,^^19^ Because mitochondrial dysfunction and neurodegeneration are major features of AD pathogenesis,^20^ we asked if aberrant phospho-S670-GRK2 regulation could play a role in AD-related neuropathologies. Our studies found aggregated phospho-S670-GRK2 in brains of AD mice and patients with dementia likely due to AD. Aggregated GRK2 triggered TOMM6 (translocase of outer mitochondrial membrane 6) aggregation and mitochondrial dysfunction, augmented amyloid-beta (Aβ), caused neurodegeneration, and increased mortality. Neurodegenerative features were retarded by GRK2 function modulation and enhanced proteasomal degradation of aggregated phospho-S670-GRK2, both by small molecules.

## Results

### Aggregated phospho-S670-GRK2 on dysfunctional mitochondria of aged AD mice

**Figure 1:**
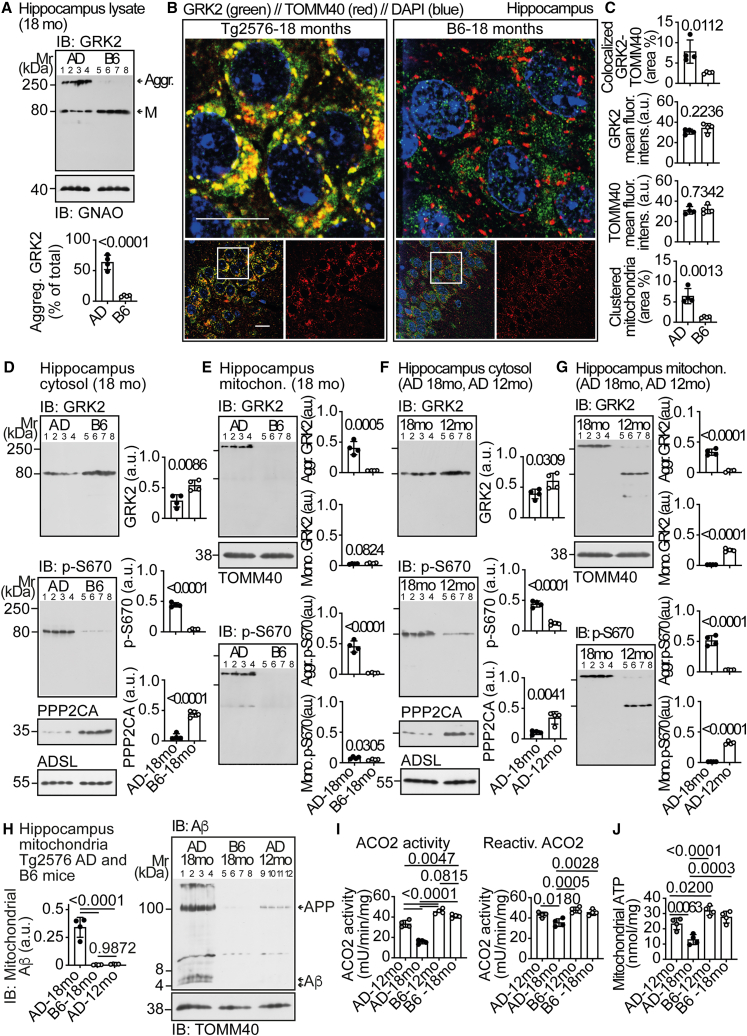
Aggregated phospho-S670-GRK2 on dysfunctional mitochondria of aged AD mice (A) Immunoblot of GRK2 in hippocampal lysates of 18-month-old Tg2576 (AD) and B6 mice. GNAO served as loading control. The lower panel shows quantitative data. (B and C) Immunofluorescence of GRK2 (green) and mitochondrial TOMM40 (red) in the CA1 hippocampal area of 18-month-old Tg2576 (left) and B6 (right) mice. Upper panels show the area marked with a white square frame in a 5-fold higher magnification (scale bars: 20 μm). Nuclei are stained with DAPI (blue). Data are from *n* = 4 mice per group. Replicates are shown in Figures S1A and S1B. Quantitative data are shown in (C) and Figures S1E–S1H. (D–G) Immunoblot of hippocampal cytosolic (D and F) and mitochondrial (E and G) GRK2 and phospho-S670-GRK2 (p-S670) in 18-month-old Tg2576 (AD-18mo), 18-month-old B6 (B6-18mo), and 12-month-old Tg2576 (AD-12mo) mice. Cytosolic PPP2CA was determined (D and F). ADSL and TOMM40 are loading controls. (H) Immunoblot of hippocampal mitochondrial Aβ (IB: Aβ) in 18-month-old Tg2576 (AD-18mo), B6 (B6-18mo), and 12-month-old Tg2576 (AD-12mo) mice. Arrowheads mark monomeric Aβ peptides and APP. TOMM40 is the loading control. (I and J) Hippocampal mitochondrial ACO2 and reactivated ACO2 activity (I) and mitochondrial ATP (J) of 12- and 18-month-old Tg2576 and B6 mice. Data are mean ± SD; *n* = 4 mice per group (A, C, D, F, I, and J) or *n* = 4 biological replicates (E, G, and H; hippocampi from three mice were pooled for one isolation, which is defined as a biological replicate); unpaired, two-tailed *t* test [df = 6; t = 9.179 (A); 3.613, 1.357, 0.3557, 5.674 (C); 3.833, 22.22, 10.66 (D); 6.815, 2.083, 9.466, 2.817 (E); 2.806, 11.00, 4.495 (F); 11.47, 18.28, 11.65, 23.38 (G)]; one-way ANOVA and Tukey’s test [F(2,9) = 55.77 (H); F(3,12) = 113.9, 12.37 (I); 21.54 (J)]. See also Figures S1A, S1B, S1E–S1H, and S2.

The role of GRK2 in AD was investigated in 18-month-old Tg2576 AD mice with substantial Aβ plaque load.^21^^,^^22^ Immunoblot analysis found that 63.5% ± 11.9% of total hippocampal GRK2 was an aggregated GRK2 species of >250 kDa in aged Tg2576 AD mice compared to only 8.5% ± 1.4% in non-transgenic B6 controls (Figure 1A). The aggregated GRK2 was stable in SDS-containing PAGE under reducing conditions and supplemented with urea (Figure 1A).

Immunofluorescence localized the (aggregated) GRK2 on hippocampi of aged AD mice and found GRK2 in the cytosol and on mitochondria where it co-localized with the translocase of outer mitochondrial membrane 40, TOMM40 (Figures 1B, 1C, S1A, S1B, and S1E). B6 mice had a predominantly cytosolic GRK2 (Figure 1B). GRK2 and TOMM40 contents were not significantly different (Figures 1B, 1C, S1F, and S1G). Hippocampi of aged AD mice showed clustered mitochondria in neuronal cell bodies covering an area of 6.4% ± 1.9% compared to only 1.3% ± 0.2% in aged B6 mice (Figures 1B, 1C, and S1H). Dysfunctional, mitochondrial aggregates in neuronal cell bodies are a typical feature of AD brains and contribute to neuronal and synaptosomal degeneration in AD patients.^23^

Because GRK2 translocation to mitochondria is induced by serine-670 phosphorylation,^16^^,^^17^ hippocampal (phospho-S670)-GRK2 contents were determined in cytosolic and mitochondrial proteins fractions (Figures 1D–1G, S2A, and S2B). Cytosolic GRK2 was slightly decreased in aged AD mice, but cytosolic phospho-S670-GRK2 was significantly higher in 18-month-old Tg2576 AD mice than in 18-month-old non-transgenic B6 and 12-month-old Tg2576 AD mice (Figures 1D and 1F). The mitochondrial fractions of 18-month-old Tg2576 mice contained aggregated (phospho-S670)-GRK2 (Figures 1E and 1G). The mitochondrial (phospho-S670)-GRK2 of 12-month-old AD mice was predominantly monomeric (Figure 1G), and mitochondrial (phospho-S670)-GRK2 was almost absent in non-transgenic B6 mice (Figure 1E). Aggregated phospho-S670-GRK2 was accompanied by decreased hippocampal PPP2CA (protein phosphatase 2 catalytic subunit alpha) levels (Figures 1D and 1F), which is a characteristic of hippocampal biopsy specimens of AD patients.^24^

Significantly increased cytosolic phospho-S670-GRK2 and aggregated mitochondrial phospho-S670-GRK2 were also present in brains from patients with dementia likely due to AD (Figures S2C–S2E). As in mice, PPP2CA levels were significantly reduced (Figures S2C and S2D).

Hippocampal phospho-S670-GRK2 aggregation was accompanied by increased mitochondrial Aβ, elevated reactive oxygen species (ROS) measured by a reduced mitochondrial aconitase (ACO2) activity, and decreased mitochondrial ATP (Figures 1H–1J). Thus, disease progression in Tg2AD 576 mice leads to aggregated phospho-S670-GRK2 and mitochondrial dysfunction.

### TOMM6 is an interaction partner of phospho-S670-GRK2

**Figure 2:**
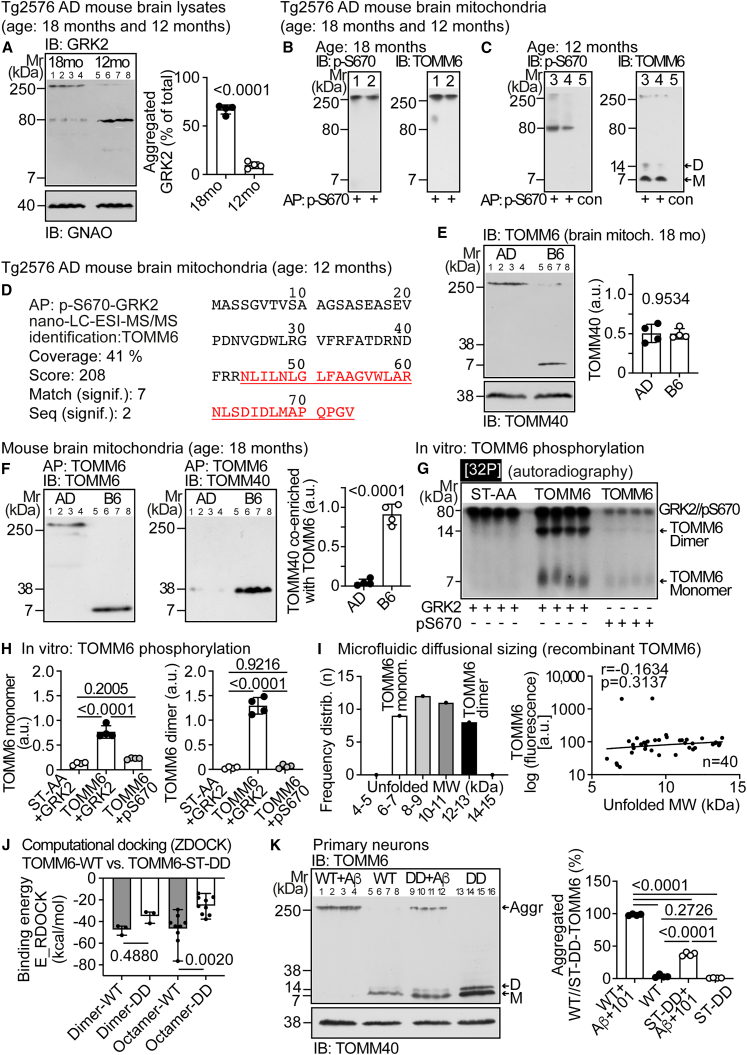
TOMM6 is an interaction partner of phospho-S670-GRK2 (A) Immunoblot of GRK2 in brain lysates of 18- and 12-month-old Tg2576 AD mice. Data are mean ± SD; *n* = 4 mice; (unpaired, two-tailed *t* test; df = 6, t = 20.02). (B and C) Immunoaffinity enrichment of phospho-S670-GRK2 (AP: p-S670, lanes 1–4) from 18-month-old (B; lanes 1 and 2) and 12-month-old (C; lanes 3 and 4) Tg2576 mouse brain mitochondria followed by immunoblot detection (IB) of enriched phospho-S670-GRK2 (left) and co-enriched TOMM6 (right). Lane 5 (C) shows a control immuno-affinity matrix (con). Two biological replicates are from 18-month-old mice, and three biological replicates are from 12-month-old Tg2576 mice. (D) Immunoaffinity enrichment of phospho-S670-GRK2 (AP: p-S670-GRK2) from brain mitochondria of 12-month-old Tg2576 mice and nano-LC-ESI-MS/MS identification of co-enriched TOMM6. (E) Immunoblot (IB) of TOMM6 (upper) and TOMM40 (lower) on brain mitochondria from 18-month-old Tg2576 and B6 mice. The right panel shows quantitative data. (F) Immunoaffinity enrichment of TOMM6 (AP: TOMM6) from brain mitochondria of 18-month-old Tg2576 (AD) and B6 (B6) mice, followed by immunoblot detection (IB) of enriched TOMM6 (left) and co-enriched TOMM40 (middle). The right panel shows quantitative data. (E and F) Data are mean ± SD; *n* = 4 biological replicates; unpaired, two-tailed *t* test [df = 6; t = 0.06099 (E), 12.59 (F)]. (G and H) *In vitro* phosphorylation of TOMM6 by GRK2 or p-S670-GRK2 (pS670). TOMM6-ST-AA was not phosphorylated by GRK2. (H) shows quantitative data; mean ± SD; *n* = 4 biological replicates; one-way ANOVA and Tukey’s test; F(2,9) = 83.12, 206.5. (I) MDS data of TOMM6 showing the frequency distribution of the unfolded molecular weight (MW) of TOMM6 (*n* = 40 measurements; left) and the linear regression analysis of the relationship between unfolded MW and TOMM6 (right). (J) Binding energies (E_RDOCK) of monomer-monomer docking, yielding dimers, and tetramer-tetramer docking yielding octamers, of wild-type TOMM6 (WT) and phosphomimetic TOMM6-ST-DD. Data represent means with range; one-way ANOVA and Tukey’s test [F(3,20) = 7.127]. (K) Immunoblot (IB) of TOMM6 in primary neurons expressing wild-type TOMM6 (WT) or TOMM6-ST-DD. Neurons were treated without and with aggregated Aβ and the GRK2 inhibitor, CMPD101 [+Aβ (immunoblot), +Aβ+101 (bar graph)]. Data represent mean ± SD; *n* = 4 biological replicates; one-way ANOVA and Tukey’s test [F(3,12) = 2141]. See also Figure S2.

To elucidate pathomechanisms triggered by (phospho-S670)-GRK2, we searched for an interaction partner. The GRK2 in brain lysates of 18-month-old Tg2576 mice was highly aggregated compared to 12-month-old Tg2576 mice (Figure 2A). Likewise, the immunoaffinity-enriched mitochondrial (phospho-S670)-GRK2 from 18-month-old Tg2576 mice was the aggregated form of >250 kDa (Figure 2B), and the enriched (phospho-S670)-GRK2 from 12-month-old Tg2576 mice was mainly the monomer of 80 ± 3 kDa (Figure 2C). The co-enrichment study identified TOMM6 as a phospho-S670-GRK2-interacting protein (Figures 2B–2D). Aggregated TOMM6 of >250 kDa was co-enriched with aggregated (phospho-S670)-GRK2 from 18-month-old AD mice (Figure 2B). Monomeric and dimeric TOMM6 of 7 ± 1 kDa and 14 ± 2 kDa were co-enriched with monomeric phospho-S670-GRK2 from 12-month-old AD mice (Figure 2C). The identity of TOMM6 was confirmed by nano-LC-ESI-MS/MS (Figure 2D). Aggregated TOMM6 was also an interaction partner of aggregated phospho-S670-GRK2 in human brains from patients with dementia likely due to AD (Figures S2F and S2G).

### Aggregated TOMM6 is dysfunctional and does not interact with TOMM40 *in vivo*

An important physiological function of TOMM6 is the assembly and stabilization of the TOMM core complex by interaction with TOMM40.^25^ In aged AD brains, TOMM6 was aggregated (Figure 2E). Co-enrichment showed that aggregated TOMM6 from AD mice did not interact with TOMM40 (Figures 2E and 2F). In contrast, TOMM40 was co-enriched with monomeric TOMM6 from non-transgenic B6 mice (Figure 2F). Thus, aggregated TOMM6 of AD brains is dysfunctional and does not interact with TOMM40.

### GRK2 phosphorylates TOMM6, whereas phospho-S670-GRK2 is inactive

Recombinant TOMM6 was phosphorylated by GRK2 *in vitro* (Figures 2G and 2H). In contrast, TOMM6 phosphorylation by phospho-S670-GRK2 was almost undetectable (Figures 2G and 2H), confirming that phospho-S670-GRK2 is largely inactive.^26^ GRK2 phosphorylates TOMM6 in the amino-terminal region, because the TOMM6-ST-AA mutant (ST-AA) with replacement of N-terminal phosphorylation sites S3, S4, T5, S9, S13, and T17 by alanine was not phosphorylated by GRK2 (Figures 2G and 2H). The autoradiogram after *in vitro* phosphorylation of TOMM6 by GRK2 revealed the TOMM6 monomer of 7 ± 2 kDa and the TOMM6 dimer of 14 ± 2 kDa (Figures 2G and 2H).

### Microfluidic diffusional sizing reveals TOMM6 monomers and dimers in solution

Microfluidic diffusional sizing (MDS) confirmed the existence of TOMM6 monomers and dimers in solution by showing that TOMM6 had a molecular weight in the range of 6–13 kDa (Figure 2I). TOMM6 dimerization did not depend on the TOMM6 protein concentration (Figure 2I, right panel). The TOMM6 dimer was also detected in previous studies with cells and was present in the cryo-EM structure of the TOMM complex, in which the TOMM6 dimer contributed to formation of a tetrameric TOMM complex.^25^

### Computational docking suggests GRK2-inactivation-dependent TOMM6 aggregation

Computational docking investigated the impact of GRK2-mediated phosphorylation on TOMM6 oligomerization by the phosphomimetic TOMM6-ST-DD mutant with GRK2 phosphorylates sites (S3-S4-T5-S9-S13-T17) replaced by aspartate. Protein-protein docking found no significant difference for the monomer-monomer interaction between wild-type TOMM6 and TOMM6-ST-DD (Figure 2J). In contrast, unphosphorylated, wild-type TOMM6 octamers were energetically favored compared to TOMM6-ST-DD octamers (Figure 2J). These *in silico* data suggest that phosphorylation of TOMM6 by GRK2 reduces the probability of TOMM6 to form aggregates. Dysfunctional TOMM6 aggregates could be triggered by inactive phospho-S670-GRK2, which disturbs GRK2-mediated TOMM6 phosphorylation.

This hypothesis was confirmed with primary neurons (Figure 2K). TOMM6-ST-DD formed significantly less aggregates than wild-type TOMM6 in neurons treated with Aβ and the GRK2 inhibitor, CMPD101, to trigger GRK2 inhibition together with ROS, like in AD brains. Without Aβ and CMPD101, wild-type TOMM6 and TOMM6-ST-DD formed monomers/dimers (Figure 2K).

### Inactive GRK2 enhances TOMM6 aggregation, Aβ plaques, and mortality, whereas wild-type GRK2 is beneficial

**Figure 3:**
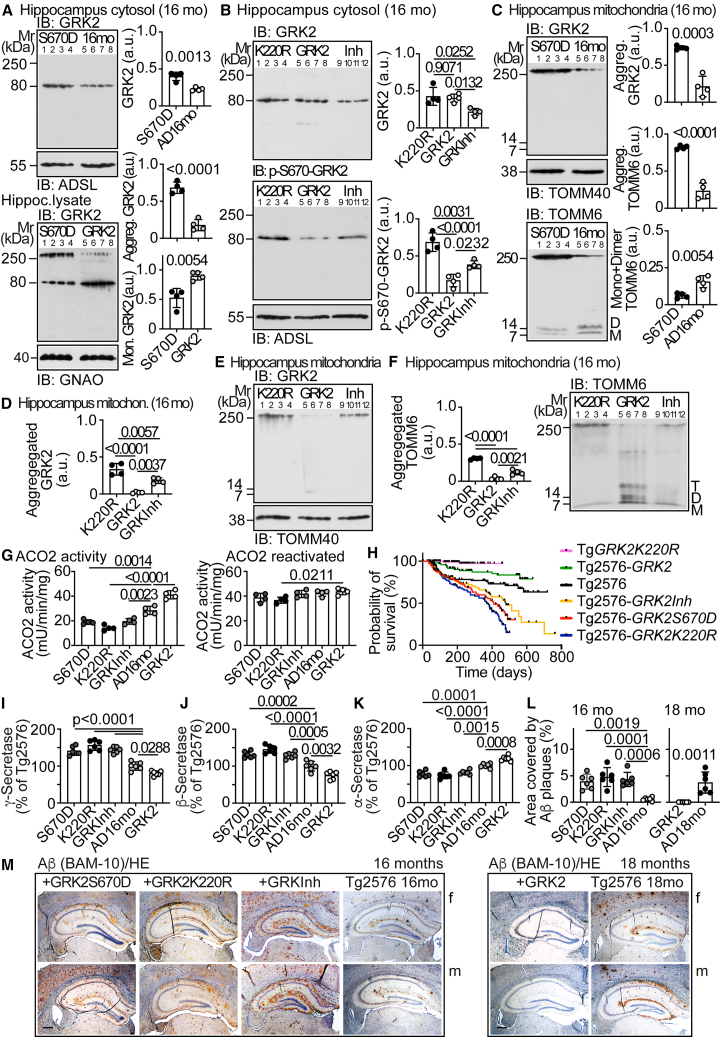
Inactive GRK2 enhances TOMM6 aggregation, Aβ plaques, and mortality, whereas wild-type GRK2 is beneficial (A) Immunoblot of hippocampal cytosolic GRK2 in 16-month-old Tg2576-*GRK2S670D* (S670D) and Tg2576 (AD16mo) mice (upper blot). The control blot detects ADSL (second blot). The third blot shows GRK2 in hippocampal lysates from 16-month-old Tg2576-*GRK2S670D* (S670D) and 16-month-old Tg2576-*GRK2* (GRK2) mice. The lower control blot detects GNAO. (B) Immunoblot of hippocampal cytosolic GRK2 (upper) and p-S670-GRK2 (middle) in 16-month-old Tg2576-*GRK2K220R* (K220R), Tg2576-*GRK2* (GRK2), and Tg2576-*GRKInh* (Inh) mice. ADSL is the cytosolic loading control. (A and B) Data represent mean ± SD; *n* = 4 mice per group; unpaired, two-tailed *t* test (A; df = 6, t = 5.640, 9.369, 4.242); one-way ANOVA and Tukey’s test [B; F(2,9) = 7.991, 31.84]. (C) Immunoblot of hippocampal mitochondrial GRK2 (upper) and mitochondrial TOMM6 (lower) in 16-month-old Tg2576-*GRK2S670D* (S670D) and Tg2576 (16 mo) mice. TOMM40 is a loading control (middle panel). (D–F) Immunoblot of hippocampal mitochondrial GRK2 (D and E) and TOMM6 (F) in 16-month-old Tg2576-*GRK2K220R* (K220R), Tg2576-*GRK2* (GRK2), and Tg2576-*GRKInh* (Inh) mice. TOMM40 is the loading control (E, lower panel). (D and F) present quantitative data. (C, D, and F) Data are mean ± SD; *n* = 4 biological replicates; unpaired, two-tailed *t* test (C; df = 6, t = 7.403, t = 10.67, t = 4.247); one-way ANOVA and Tukey’s test [D,F; F(2,9) = 38.19, 137.0]. (G) Hippocampal mitochondrial ACO2 activity without (left) and with reactivation (right) in 16-month-old Tg2576-*GRK2S670D* (S670D), Tg2576-*GRK2K220R* (K220R), Tg2576-*GRKInh* (GRKInh), and Tg2576-*GRK2* (GRK2) mice in comparison to 16-month-old Tg2576 (AD16mo) mice. Data are mean ± SD; *n* = 4 mice per group; one-way ANOVA and Tukey’s test; F(4,15) = 63.00 (left), 4.111 (right). (H) Probability of survival of the indicated transgenic mouse lines with Kaplan-Meier survival analysis and log rank (Mantel-Cox) test; *n* = 103 male and female mice (*n* = 136 for Tg-*GRK2K220R*); df = 1; chi-square 4.940 and *p* = 0.0264 for Tg2576-*GRK2* vs. Tg2576; chi-square 16.83 and *p* < 0.0001 for Tg2576-*GRK2S670D* vs. Tg2576; df = 1; chi-square 20.70 and *p* < 0.0001 for Tg-*GRK2K220R* vs. Tg2576; chi-square 7.459 and *p* = 0.0063 for Tg2576-*GRKInh* vs. Tg2576; chi-square 28.51 and *p* < 0.0001 for Tg2576-*GRK2K220R* vs. Tg2576. (I–K) Hippocampal γ-secretase (I), β-secretase (J), and α-secretase (K) of 16-month-old Tg2576-*GRK2S670D* (S670D), Tg2576-*GRK2K220R* (K220R), Tg2576-*GRKInh* (GRKInh), and Tg2576-*GRK2* (GRK2) mice compared to Tg2576 (AD16mo) mice. Data represent mean ± SD; *n* = 6 mice per group; one-way ANOVA and Tukey’s test; F(4,25) = 55.73 (I), 44.06 (J), 41.47 (K). (L) Aβ plaque load of histological sections shown in (M); Figures S3E and S4C. Data are mean ± SD; *n* = 6 mice per group; one-way ANOVA and Tukey’s test [left panel; F(3,20) = 12.13]; unpaired, two-tailed *t* test (right panel; df = 10, t = 4.537). (M) Hippocampal Aβ plaques (brown) of 16-month-old Tg2576-*GRK2S670D*, Tg2576-*GRK2K220R*, Tg2576-*GRKIn*h, and Tg2576 (Tg2576 16mo) mice and 18-month-old Tg2576-*GRK2* and Tg2576 (Tg2576 18mo) female (f) and male (m) mice. Counterstaining was performed with hematoxylin (HE). Immunohistology is representative of six mice per group; scale bars: 200 μm. Replicates are shown in Figures S3E and S4C. See also Figures S3 and S4.

Is the inactive phospho-S670-GRK2 causally involved in TOMM6 aggregation *in vivo*, as suggested? We used the phosphomimetic GRK2-S670D, which is inactive like serine-670-phosphorylated GRK2,^26^ and generated double-transgenic Tg2576-*GRK2S670D* mice (Figure S3A), with 1.8-fold higher cytosolic hippocampal GRK2-(S670D) than single-transgenic Tg2576 AD mice (Figure 3A). Cytosolic GRK2-(S670D) was monomeric, whereas GRK2-(S670D) aggregation was prominent in total hippocampal protein lysates (Figure 3A). GRK2-(S670D) aggregation was not caused by increased protein levels of GRK2-S670D in Tg2576-*GRK2S670D* mice because hippocampal lysates from Tg2576-*GRK2* mice with comparably increased wild-type GRK2 levels showed predominantly GRK2 monomers (Figure 3A).

Phospho-S670-GRK2 and phosphomimetic GRK2-S670D have a strongly impaired kinase activity.^26^ To determine the impact of GRK2 inactivation *in vivo*, we used (1) the kinase-function-deficient mutant, GRK2-K220R,^27^ in double-transgenic Tg2576-*GRK2K220R* mice and (2) the peptide GRK(2) inhibitor, GRKInh,^28^ in double-transgenic Tg2576-*GRKInh* mice. As a control, Tg2576-*GRK2* mice with transgenic expression of wild-type GRK2 were generated. The cytosolic hippocampal GRK2 proteins appeared as monomers in all double-transgenic Tg2576 mouse lines (Figures 3A, 3B, and S4A).

Inactive GRK2 proteins (S670D, K220R, and GRKInh-inhibited) were aggregated on hippocampal mitochondria of 16-month-old, double-transgenic AD mice and accompanied by TOMM6 aggregation, mitochondrial dysfunction, and increased ROS (Figures 3C–3G). In contrast, increased transgenic wild-type GRK2 did not accumulate on mitochondria, prevented TOMM6 aggregation, and led to reduced ROS, as documented by the increased ACO2 activity of Tg2576-*GRK2* mice (Figures 3E–3G and S4B). Mitochondrial ROS enhances the generation of Aβ and Aβ plaques.^20^^,^^23^ With increased mitochondrial ROS due to inactive GRK2, accumulation of insoluble, soluble, and mitochondrial Aβ was accelerated (Figures S3B–S3D). Increased (soluble) Aβ levels are neurotoxic^29^ and could account for the higher mortality of double-transgenic AD mice with inactive GRK2 (Figure 3H). Complementary to previous studies with cells,^30^ pro-amyloidogenic β-secretase and γ-secretase activities were enhanced by inactive GRK2 while beneficial α-secretase activity was reduced (Figures 3I–3K). Immunohistology showed increased hippocampal Aβ plaques in AD mice with inactive GRK2 (Figures 3L, 3M, and S3E). Vice versa, wild-type GRK2 led to an improved survival of AD mice, reduced pro-amyloidogenic APP-processing enzyme activities, and decreased Aβ peptides and Aβ plaques (Figures 3H–3M and S4C–S4G).

Data from different transgenic Tg2576 mouse lines with inactive phospho-S670-GRK2 aggregates could be relevant for the human disease because human brain specimens from patients with dementia likely due to AD were also characterized by increased inactive phospho-S670-GRK2 and TOMM6 aggregates (Figures S2C–S2G).

### GRK2 activities differ from ARRB1-mediated effects

ARRB1 is the direct target of GRK2.^7^ We asked whether ARRB1 exerted similar effects as GRK2 and generated double-transgenic Tg2576-*ARRB1* mice (Figure S3A). Aged Tg2576-*ARRB1* mice with 2.6-fold increased hippocampal cytosolic ARRB1 had reduced Aβ plaques and lower insoluble Aβ than Tg2576 mice (Figures S4C, S4D, S4F, and S4I). Surprisingly, the γ-secretase activity and soluble Aβ peptides were higher in Tg2576-*ARRB1* mice (Figures S4E and S4G). The increased γ-secretase activity could be caused by the interaction of ARRB1 with APH1A (Figure S4H), which favors γ-secretase protein complex formation.^31^ Cytosolic phospho-S670-GRK2 was slightly higher in Tg2576-*ARRB1* mice than in Tg2576 mice (Figures S4J and S4K). Despite increased phospho-S670-GRK2, and more soluble and mitochondrial Aβ, the hippocampal mitochondrial function was improved by ARRB1, as documented by increased mitochondrial ATP, and TOMM6 and mitochondrial GRK2 aggregates were reduced (Figures S4L–S4P). Thus, effects of ARRB1 differ from GRK2 in Tg2576 AD mice.

### Neuron-specific TOMM6 counteracts insoluble Aβ aggregation but increases soluble Aβ and mortality

**Figure 4:**
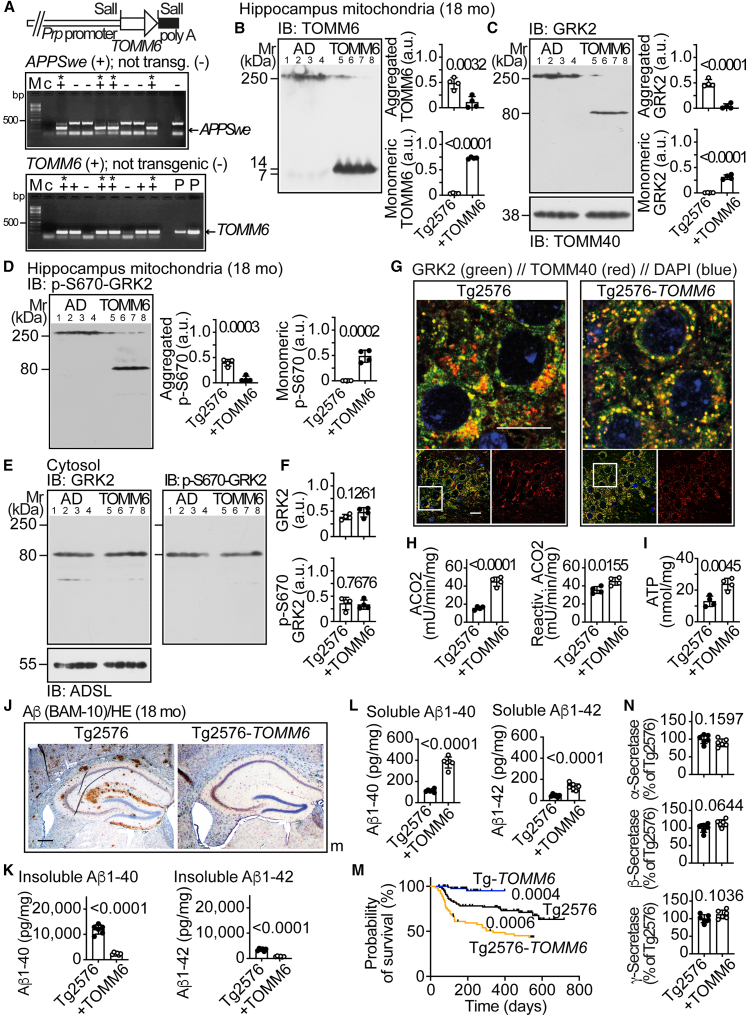
Neuron-specific TOMM6 counteracts insoluble Aβ aggregation but increases soluble Aβ and mortality (A) Plasmid for generation of Tg2576-*TOMM6* mice (upper) and PCR genotyping (middle and lower panels); c, control without genomic DNA; P, positive plasmid control; M, DNA marker. Double-transgenic mice are marked with an asterisk. (B–D) Immunoblot of hippocampal mitochondrial TOMM6 (B), GRK2 (C), and phospho-S670-GRK2 (D) of 18-month-old Tg2576 and Tg2576-*TOMM6* (+TOMM6) mice. The control immunoblot (C, lower panel) detects TOMM40. (E and F) Immunoblot of hippocampal, cytosolic GRK2 (E, left) and phospho-S670-GRK2 (E, right) of 18-month-old Tg2576 and Tg2576-*TOMM6* (+TOMM6) mice. The control blot detects ADSL. Quantitative data are shown in (F). (B, C, D, and F) Data are mean ± SD; *n* = 4 biological replicates per group (B, C, and D) and *n* = 4 mice per group (F); unpaired, two-tailed *t* test; df = 6; t = 4.741, 74.19 (B); 10.33, 13.47 (C); 7.329, 8.060 (D); 1.776, 0.3092 (F). (G) Immunofluorescence of GRK2 (green) and TOMM40 (red) in the hippocampal CA1 area of 18-month-old Tg2576 (left) and Tg2576-*TOMM6* (right) mice. Nuclei are stained with DAPI (blue). The upper panels show the area marked by a white frame in a 5-fold higher magnification (scale bars: 20 μm). Immunofluorescence is representative of *n* = 4 mice per group. Replicates and quantitative data are shown in Figures S1A, S1C, and S1E–S1H. (H and I) Hippocampal mitochondrial ACO2 activity (H) and mitochondrial ATP (I) of 18-month-old Tg2576 and Tg2576-*TOMM6* (+TOMM6) mice. Data are mean ± SD (*n* = 4 mice per group); unpaired, two-tailed *t* test; df = 6; t = 10.77, 3.346 (H); t = 4.420 (I). (J) Immunohistology of hippocampal Aβ plaques (brown) of 18-month-old Tg2576 and Tg2576-*TOMM6* male (m) mice. Nuclei were stained with hematoxylin (HE). Immunohistology is representative of six mice per group (scale bar: 200 μm). Replicates and quantitative data are shown in Figures S5A and S5B. (K and L) Insoluble (K) and soluble (L) Aβ1-40 and Aβ1-42 in brains of 18-month-old Tg2576 and Tg2576-*TOMM6* (+TOMM6) mice. Data are mean ± SD; *n* = 6 mice per group; unpaired, two-tailed *t* test; df = 10; t = 12.59, 13.23 (K); 12.06, 9.365 (L). (M) Probability of survival of male and female Tg2576-*TOMM6* (*n* = 62), Tg2576 (*n* = 103), and Tg-*TOMM6* mice (*n* = 103) was performed by Kaplan-Meier survival analysis with log rank (Mantel-Cox) test; df = 1, chi-square 12.52 and *p* = 0.0004 for Tg-*TOMM6* vs. Tg2576; chi-square 11.7 and *p* = 0.0006 for Tg2576-*TOMM* vs. Tg2576. (N) Hippocampal α-, β-, and γ-secretase in 18-month-old Tg2576 and Tg2576-*TOMM6* mice. Data represent mean ± SD; *n* = 6 mice per group (unpaired, two-tailed *t* test; df = 10, t = 1.519, 2.078, 1.791). See also Figures S1A, S1C, S1E–S1H, and S5.

Because ARRB1 reduced TOMM6 aggregation and Aβ plaques, we focused on TOMM6 as a possible target. TOMM6 is an essential protein, and *Tomm6* deficiency in mice causes a phenotype of premature aging, with an early death at an age of 4–5 weeks^32^ To replenish functional TOMM6, we generated Tg2576-*TOMM6* mice with neuron-specific expression of *TOMM6* under control of the neuron-specific prion protein (*Prp*) promoter (Figure 4A).

Increased neuronal *TOMM6* largely prevented dysfunctional TOMM6 aggregation (Figure 4B). Mitochondrial GRK2 and phospho-S670-GRK2 aggregates were significantly lower in Tg2576-*TOMM6* mice but mitochondrial phospho-S670-GRK2 monomers were present (Figures 4C and 4D). Cytosolic GRK2 and phospho-S670-GRK2 were unaltered (Figures 4E and 4F). Immunofluorescence revealed evenly distributed mitochondria with significantly less clustering and the presence of mitochondrial GRK2 on hippocampal specimens of Tg2576-*TOMM6* mice (Figures 4G, S1A, S1C, and S1E–S1H).

Increased functional TOMM6 improved the mitochondrial function, reduced hippocampal ROS, and increased ATP (Figures 4H and 4I). Aβ plaques and insoluble Aβ were also strongly reduced (Figures 4J, 4K, S5A, and S5B). Surprisingly, soluble Aβ and mitochondrial Aβ were significantly higher in brains of Tg2576-*TOMM6* mice compared to single-transgenic Tg2576 mice (Figures 4L, S5C, and S5E). Moreover, the senescence marker, urokinase plasminogen activator surface receptor (UPAR), was elevated (Figures S5D and S5F), and the mortality of Tg2576-*TOMM6* mice was increased (Figure 4M). Activities of APP-processing enzymes were not significantly altered (Figure 4N). Thus, with sustained amyloidogenic APP-processing, soluble Aβ is continuously generated and accumulates intracellularly. Soluble Aβ from *APP*^*Swe*^ induces neurotoxicity,^29^ which could account for accelerated aging and increased mortality of Tg2576-*TOMM6* mice. In contrast, single-transgenic Tg-*TOMM6* mice without *APP*^*Swe*^ had no increased mortality (Figure 4M).

### Development of a GRK2 function modulator that prevents phospho-S670-GRK2 and TOMM6 aggregation

**Figure 5:**
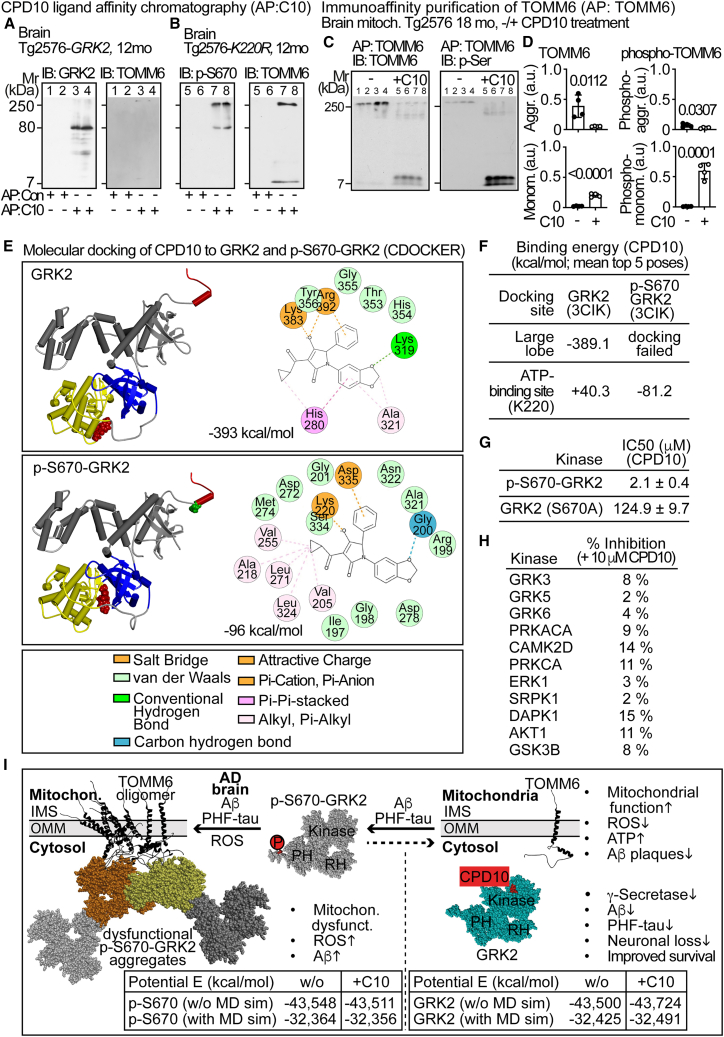
Development of a GRK2 function modulator that prevents phospho-S670-GRK2 and TOMM6 aggregation (A) CPD10 ligand affinity chromatography (AP:C10) with brain lysates of 12-month-old Tg2576-*GRK2* mice. CPD10-interacting GRK2 (lanes 3 and 4; left) was eluted from the CPD10-containing affinity matrix and detected by immunoblot (IB). TOMM6 did not interact and was below detection limit (lanes 3 and 4; right). Lanes 1 and 2 show a control affinity matrix (AP: Con). (B) Enrichment of p-S670-GRK2(K220R) (lanes 7 and 8; left) and co-enrichment of TOMM6 (lanes 7 and 8; right) by CPD10 ligand affinity chromatography (AP:C10) from brains from 12-month-old Tg2576-*GRK2K220R* mice. Lanes 5 and 6 show a control affinity matrix. (A and B) Two biological replicates are shown for each transgenic mouse line. (C and D) Immunoaffinity enrichment (AP) of TOMM6 from brain mitochondria of 18-month-old Tg2576 mice without or with CPD10 treatment (±C10) for 6 months followed by immunoblot detection of enriched TOMM6 (IB: TOMM6) and phosphorylated TOMM6 (IB: p-Ser). Representative immunoblots (C) and quantitative data (D) are shown. Data represent mean ± SD; *n* = 4 biological replicates per group; unpaired, two-tailed *t* test; df = 6; t = 3.611 (TOMM6 aggr.), 11.74 (TOMM6 monomer), 2.811 (phospho-TOMM6 aggr.), 9.016 (phospho-TOMM6 monomer). (E) Molecular docking of CPD10 (CPK model, red) to GRK2 (upper) and to p-S670-GRK2 (middle) by CDOCKER. Left images show GRK2 and p-S670-GRK2 with modeled C-terminus (red), the large lobe (yellow), the small lobe (blue), and N-terminal and C-terminal domains of GRK2 (gray). The S670 phosphorylation is shown in green (CPK model). Right panels show the docking site of CPD10 in the large lobe of GRK2 (upper) and in the ATP-binding site of p-S670-GRK2 (middle). (F) Binding energies (kcal/mol) of CPD10 (S-isomer) docking to GRK2 and phospho-S670-GRK2. Data are mean of top five poses ± SD: −389.1 ± 12.1; +40.3 ± 32.7; −81.2 ± 30.3. (G) IC_50_ values of CPD10 for inhibition of TOMM6 phosphorylation by p-S670-GRK2 and GRK2-S670A (S670A). Data are mean ± SD; *n* = 4 independent experiments with four technical replicates each. (H) CPD10 (10 μM) did not substantially inhibit GRK2-related kinases. (I) Conceptual model and molecular dynamics simulation of GRK2 function modulation by CPD10. CPD10 could stabilize the GRK2 monomer (cyan, right) and prevent phospho-S670-GRK2 and TOMM6 aggregation (left); OMM: outer mitochondrial membrane; IMS: intermembrane space. Potential energies (E) of GRK2 (lower right) and phospho-S670-GRK2 (p-S670; lower left) without (w/o) and with CPD10 (+C10) interaction are given after energy minimization without (w/o MD sim) and with MD simulation (MD sim). See also Figure S6 and Table S1.

Restoration of TOMM6 only counteracted ROS-dependent protein aggregation and Aβ plaque formation but had no major effects on other neuropathological features triggered by inactive phospho-S670-GRK2 such as the amyloidogenic processing of APP. Because wild-type GRK2 reduced phospho-S670-GRK2 aggregation and major neuropathological AD features, we aimed to prevent phospho-S670-GRK2 aggregation with a small GRK2-stabilizing molecule. Compound development focused on small molecules that contain the benzodioxole moiety of paroxetine as a prototypic GRK2-interacting molecule.^33^ By this approach, CPD10 (1-(1,3-benzodioxol-5-yl)-4-(cyclopropanecarbonyl)-3-hydroxy-2-phenyl-2H-pyrrol-5-one) was developed. CPD10 ligand affinity chromatography showed that CPD10 interacts with non-phosphorylated GRK2 and phospho-S670-GRK2 in brain lysates from AD mice (Figures 5A and 5B). TOMM6, as the mitochondrial interaction partner of phospho-S670-GRK2 (Figures 2B–2D), was not (co-)enriched by CPD10 from Tg2576-*GRK2* brains with predominantly non-phosphorylated GRK2 (Figure 5A). In contrast, monomeric and aggregated TOMM6 were co-enriched by the CPD10 affinity matrix when phospho-S670-GRK2(K220R)-containing brain samples were applied (Figure 5B).

In a cellular AD model of primary cortical neurons, in which aggregated Aβ1-42 increased mitochondrial phospho-S670-GRK2 aggregation (Figure S6A), CPD10 enhanced the active, non-phosphorylated GRK2 and prevented mitochondrial GRK2 and TOMM6 aggregation (Figures S6B and S6C). CPD10 also prevented the Aβ1-42-induced mitochondrial dysfunction, as measured by ACO2 activity, with an EC50 value of 155.6 ± 19.6 nM (Figures S6D and S6E). Likewise, CPD10 largely prevented the TOMM6 aggregation in brains of Tg2576 AD mice (Figures 5C and 5D). In agreement with prevention of TOMM6 aggregation by GRK2-mediated phosphorylation, TOMM6 monomers/dimers were highly phosphorylated (Figures 5C and 5D).

Molecular docking identified binding sites of CPD10 on GRK2 and phospho-S670-GRK2. Because the carboxyl-terminal domain of GRK2 with residues S670-L689 was not resolved in crystal structures,^34^^,^^35^ the carboxyl-terminal tail was added by homology modeling (Figure 5E). CPD10 docked to a binding site in the kinase large lobe of GRK2, with a highly favorable binding energy of −389.1 kcal/mol (Figures 5E and 5F). CPD10 docked preferentially to non-phosphorylated GRK2, whereas docking of CPD10 to these residues of phospho-S670-GRK2 failed (Figures 5E and 5F). Instead, CPD10 was docked with an average binding energy of −81.2 kcal/mol to a binding site surrounding K220 and D335 of phospho-S670-GRK2 (Figures 5E and 5F), which are part of the triphosphate subsite of the ATP-binding site and essential for kinase activity.^34^ Binding of CPD10 to this binding sphere in the non-phosphorylated GRK2 was unfavorable with a positive binding energy of +40.3 kcal/mol (Figures 5E and 5F). Congruent with molecular docking, CPD10 inhibited the phospho-S670-GRK2 with an IC_50_ value of 2.2 ± 0.5 μM, whereas only a high concentration of >100 μM affected the kinase activity of wild-type GRK2 (Figure 5G).

Other kinases were not inhibited by 10 μM of CPD10 (Figure 5H). Furthermore, CPD10 did not inhibit the monoamine transporters for serotonin, noradrenaline, and dopamine, had no off-target effects on more than 40 CNS-related pharmacological targets, and did not inhibit the acetylcholinesterase (Table S1).

### Molecular dynamics simulations of GRK2 function modulation by CPD10

Based on the data, the following conceptual model is proposed. CPD10 binds to GRK2 and phospho-S670-GRK2. Because of energetically favored binding of CPD10 to GRK2, the equilibrium between GRK2 and phospho-S670-GRK2 could be shifted toward GRK2. This concept is supported by a molecular dynamics simulation protocol for proteins.^36^ CPD10 induced a favorable decrease of the potential energy of GRK2, without and with molecular dynamics (MD) simulations (Figure 5I, right). In contrast, the potential energy of phospho-S670-GRK2 was unfavorably higher after interaction with CPD10 (Figure 5I, left). Thus, CPD10 could stabilize the non-phosphorylated GRK2 and destabilize the aggregation-prone phospho-S670-GRK2 (Figures S6B and S6C). This is relevant because accumulated phospho-S670-GRK2 forms oligomers, which could be caused by dysfunctional Gβγ-GRK2 interaction.^26^^,^^37^ Oligomers of phospho-S670-GRK2 could become covalent aggregates in AD brains due to increased mitochondrial ROS, triggered by AD hallmark proteins, Aβ and PHF-tau (Figure 5I). Aggregated, inactive phospho-S670-GRK2 enhances TOMM6 aggregation, and traps its substrate TOMM6 in a neurodegenerative protein aggregation complex (Figure 5I). CPD10 shifts the equilibrium from aggregation-prone phospho-S670-GRK2 toward monomeric, non-phosphorylated GRK2. Thereby, CPD10 prevents TOMM6 aggregation and the build-up of harmful phospho-S670-GRK2-TOMM6 protein aggregates and could counteract major AD symptoms (Figure 5I).

### GRK2 function modulation counteracts dysfunctional phospho-S670-GRK2 aggregation and retards AD progression and mortality in mice

**Figure 6:**
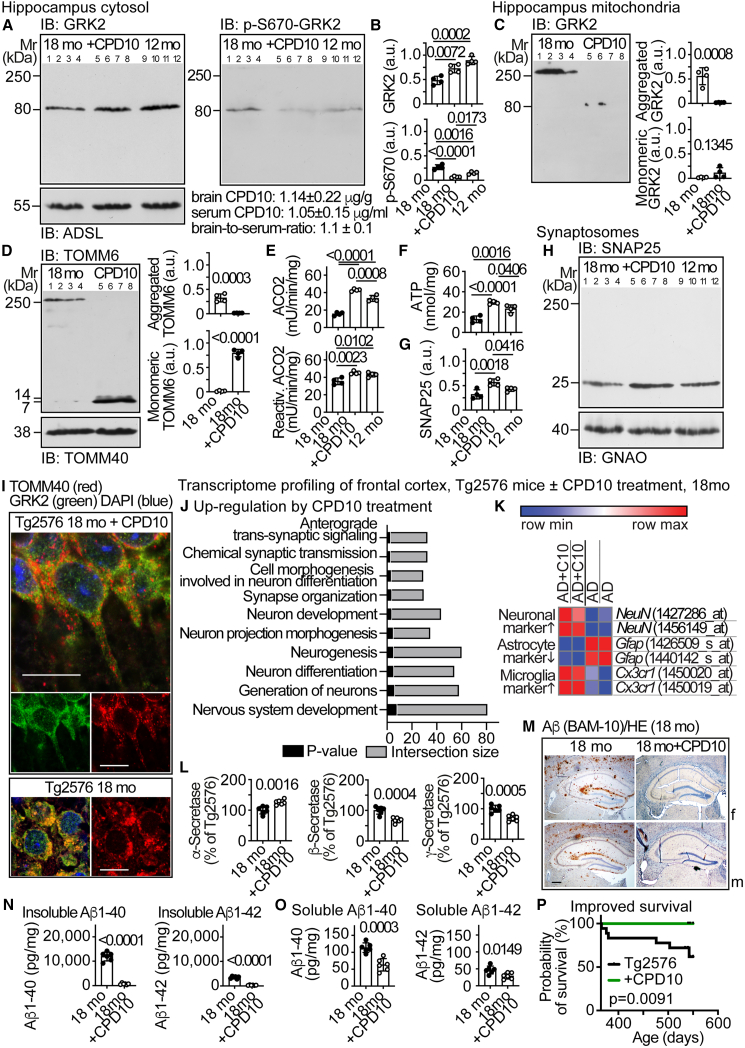
GRK2 function modulation counteracts dysfunctional phospho-S670-GRK2 aggregation and retards AD progression and mortality in mice (A and B) Immunoblot of hippocampal, cytosolic GRK2 (A, left) and p-S670-GRK2 (A, right) of 18-month-old, untreated (18 mo), CPD10-treated (18mo + CPD10; 8 mg/kg/d), and 12-month-old, untreated (12 mo) Tg2576 mice. The control blot detects ADSL. Data are mean ± SD; *n* = 4 mice; one-way ANOVA and Tukey’s test; F(2,9) = 23.54, 37.45 (B). Below the right panel (A), brain and serum concentrations, and brain-to-serum ratios of CPD10 (mean ± SD; *n* = 6 mice) are shown. See also Figures S5G and S5H. (C and D) Immunoblot of hippocampal, mitochondrial GRK2 (C) and TOMM6 (D) of 18-month-old, untreated (18 mo) and CPD10-treated (18mo + CPD10) Tg2576 mice. The control blot detects TOMM40. Data represent mean ± SD; *n* = 4 biological replicates; unpaired, two-tailed *t* test; df = 6; t = 6.179, 1.729 (C); t = 7.540, 20.48 (D). (E and F) Hippocampal mitochondrial ACO2 activity without and with reactivation (E), and ATP contents (F) were determined in 18-month-old, untreated (18 mo), CPD10-treated (18mo + CPD10), and 12-month-old, untreated (12 mo) Tg2576 mice. Data represent mean ± SD (*n* = 4 mice per group); one-way ANOVA and Tukey’s test; F(2,9) = 135.2, 13.11 (E); 30.06 (F). (G and H) Immunoblot of synaptosomal, hippocampal SNAP25 in 18-month-old, untreated (18 mo), CPD10-treated (18mo + CPD10), and 12-month-old, untreated (12 mo) Tg2576 mice. The control blot detects GNAO. Data are mean ± SD (*n* = 4 mice per group); one-way ANOVA and Dunnett’s test; F(2,9) = 11.64 (G). (I) Immunofluorescence of mitochondrial TOMM40 (red) and GRK2 (green) in the hippocampal CA1 area of brain specimens from 18-month-old, CPD10-treated (Tg2576 18mo + CPD10) and untreated (Tg2576 18mo) Tg2576 mice. Nuclei were stained with DAPI (blue); scale bars: 20 μm. Immunofluorescence is representative of *n* = 7 mice per group. Three replicates are shown in Figure S7. Four replicates are shown in Figures S1A and S1D. (J) Overrepresentation analysis of significantly up-regulated transcripts (≥2-fold; *p* < 0.05) in frontal cortices of 18-month-old CPD10-treated Tg2576 mice compared to untreated mice. Biological processes (GO:BP) with *p* values <0.001 in the overrepresentation analysis and involvement in neurogenesis are shown. *p* values are presented as negative log10 of adjusted *p* values. Data are shown in Table S5. (K) Heatmap of significantly different (*p* < 0.05) transcripts (*NeuN*, *Gfap*, and *Cx3cr1*) in frontal cortices of 18-month-old, CPD10-treated Tg2576 (AD + C10) compared to untreated Tg2576 mice (AD). Data are shown in Table S2. (L) Activities of hippocampal α-, β-, and γ-secretase in 18-month-old, untreated (18 mo) and CPD10-treated (18mo + CPD10) Tg2576 mice. Data represent mean ± SD (*n* = 6 mice per group); unpaired, two-tailed *t* test; df = 10, t = 4.303, 5.280, 5.049. (M) Immunohistological detection of Aβ plaques (brown) on coronal brain sections of 18-month-old, untreated (18 mo) and CPD10-treated (18mo + CPD10) female (f) and male (m) Tg2576 mice. Counterstaining was performed with hematoxylin (HE). Immunohistology is representative of six mice per group (scale bar: 200 μm). Replicates and quantitative data are shown in Figures S5A and S5B. (N and O) Insoluble (N) and soluble (O) Aβ1-40 and Aβ1-42 in brains of 18-month-old, untreated (18 mo) and CPD10-treated (18mo + CPD10) Tg2576 mice. Data represent mean ± SD (*n* = 6 mice per group); unpaired, two-tailed *t* test; df = 10; t = 14.91, 16.01 (N); 5.352, 2.937 (O). (P) Probability of survival of CPD10-treated (+CPD10) and untreated Tg2576 mice (*n* = 18 mice) was determined by Kaplan-Meier survival analysis with log rank (Mantel-Cox) test; df = 1, chi-square = 6.800. See also Figures S1A, S1D–S1H, S5, S7, and S8; Tables S2 and S5.

Does CPD10 exert the above-suggested effects *in vivo*? Six months of CPD10 treatment slightly increased cytosolic, hippocampal GRK2 and reduced phospho-S670-GRK2 in 18-month-old Tg2576 mice (Figures 6A and 6B). The six-month treatment period from 12 to 18 months is the time when Aβ plaques accumulate in Tg2576 mouse brain.^21^^,^^22^ CPD10 efficiently penetrated the blood-brain barrier with brain levels of 1.14 ± 0.22 μg/g, serum levels of 1.05 ± 0.15 μg/mL, and a brain-to-serum ratio of 1.10 ± 0.10 (Figures 6A, S5G, and S5H). CPD10 largely prevented dysfunctional GRK2 and TOMM6 aggregates on hippocampal mitochondria and improved the mitochondrial function with higher mitochondrial ACO2 activities and ATP levels than untreated, 18- and 12-month-old Tg2576 mice (Figures 6C–6F).

Mitochondrial dysfunction promotes synaptic degeneration and loss of synaptic proteins such as SNAP25.^23^ CPD10 significantly increased SNAP25 in brains of 18-month-old, treated Tg2576 mice compared to untreated 18- and 12-month-old Tg2576 mice (Figures 6G and 6H).

Immunofluorescence showed largely evenly distributed mitochondria in neuronal cell bodies and axons of CPD10-treated Tg2576 hippocampi, whereas untreated mice showed prominent mitochondrial clustering (Figures 6I, S1A, S1D, S1E–S1H, and S7A–S7D).

Microarray gene expression analysis of frontal cortices from 18-month-old Tg2576 mice documented that CPD10 significantly up-regulated transcripts related to nervous system development (Figure 6J; Table S5). CPD10-induced neurogenesis was documented by the increased neuronal marker, *NeuN* (Figure 6K; Table S2). The inflammatory gliosis response, a typical feature of AD brains,^38^ was reduced by CPD10, as reflected by the reduced astrocyte marker, *Gfap* (Figure 6K). The AD protective and Aβ-clearing microglia marker, *Cx3cr1*,^39^ was increased (Figure 6K).

CPD10 shifted the APP-processing activities toward the non-amyloidogenic route (Figure 6L). Hippocampal Aβ plaques, insoluble and soluble Aβ, and mitochondrial Aβ were strongly reduced by CPD10 treatment (Figures 6M–6O, S5A–S5C, and S5E) in a dose-dependent manner (Figures S5G–S5I).

By reduction of neurotoxic Aβ peptides, CPD10-treated Tg2576 mice were protected from the increased Aβ-related mortality (Figure 6P). In addition, hippocampal contents of the senescence marker, UPAR, were significantly reduced (Figures S5D and S5F). Thus, the GRK2 function modulator counteracted major neurodegenerative AD symptoms, promoted neurogenesis, slowed senescence, and enhanced survival.

### GRK2 function modulation retards neurodegenerative PHF-tau hyperphosphorylation and neuronal loss

Aβ and hyperphosphorylated PHF-tau are the two major AD hallmark proteins. Hippocampal PHF-tau was enhanced in 14-month-old, male Tg2576 mice by 2 months of chronic unpredictable mild stress, CUMS.^22^ Treatment with CPD10 during the CUMS protocol prevented the CUMS-enhanced PHF-tau (Figures S8A, S8B, S8K, S8L, and S8N). CPD10 maintained inhibitory phosphorylation of the tau kinase, GSK3B, at serine-9 (Figure S8C). These effects could be mediated by CPD10-mediated restoration of GRK2, which enhances inhibitory GSK3B phosphorylation.^40^ CPD10 also retarded the CUMS-induced neuronal loss and improved the spatial memory of CUMS-subjected Tg2576 mice in the Morris water maze test (Figures S8D and S8E).

CPD10 also reduced the CUMS-induced mitochondrial phospho-S670-GRK2 aggregates (Figure S8F). Phospho-S670-GRK2 aggregation in CUMS-subjected Tg2576 mice was triggered by increased PHF-tau because 12-month-old, double-transgenic Tg2576-MAPT∗P301L (Tg2576-TAU) mice with increased brain and mitochondrial PHF-tau also showed significantly higher mitochondrial phospho-S670-GRK2 aggregates than age-matched, single-transgenic Tg2576 mice without increased PHF-tau (Figures S8G–S8I).

Immunofluorescence found co-localization of (aggregated) phospho-S670-GRK2 with PHF-tau on clustered mitochondria of cortex specimens from CUMS-subjected Tg2576 mice and double-transgenic Tg2576-MAPT∗P301L mice, with Pearson coefficients of 0.67 ± 0.04 and 0.59 ± 0.08, respectively (Figures S8J–S8N). Mitochondrial PHF-tau was confirmed by immunoblot (Figure S8H) and by co-localization with the mitochondrial TOMM40 (Figure S8O). There was a prominent mitochondrial co-localization of inactive phospho-S670-GRK2 with hyperphosphorylated PHF-tau in brains of CUMS-subjected Tg2576 mice and Tg2576-MAPT∗P301L mice (Figures S8F and S8I–S8O). This co-localization strongly indicates that inactive phospho-S670-GRK2 does not substantially inhibit tau phosphorylation *in vivo*, most likely because GRK2 is only a weak tau kinase.^12^ Moreover, the co-localization of GRK2 with cytosolic PHF-tau and PHF-tau(-P301L) was low, with Pearson coefficients of 0.18 ± 0.03 and 0.24 ± 0.03 (Figure S8J).

Taken together, CPD10 prevented dysfunctional GRK2 and TOMM6 aggregation, mitochondrial dysfunction, and major neurodegenerative AD symptoms including Aβ, synaptic degeneration, PHF-tau, and neuronal loss.

### A proteasome activity enhancer augments phospho-S670-GRK2 degradation and retards neurodegenerative AD symptoms

**Figure 7:**
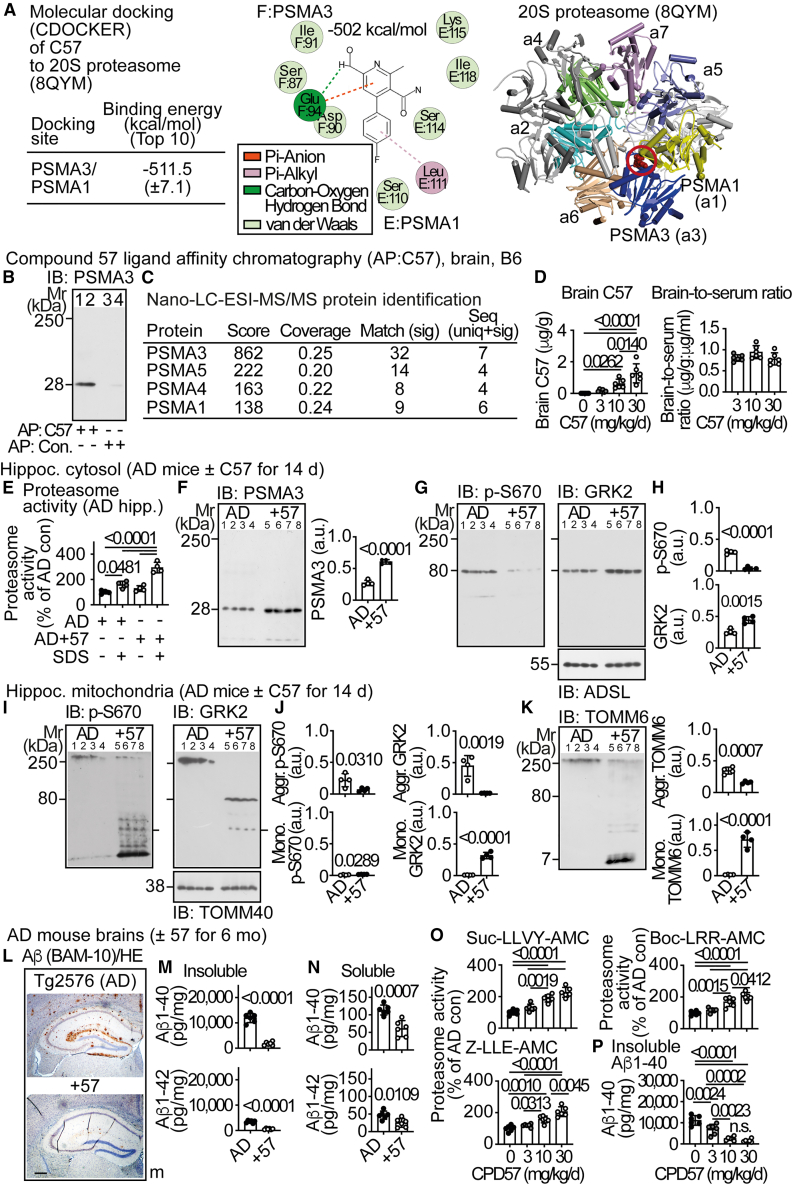
A proteasome activity enhancer augments phospho-S670-GRK2 degradation and retards neurodegenerative AD symptoms (A) Binding energy (left), docking site (middle), and molecular docking of CPD57 to PSMA1 (chain E) and PSMA3 (chain F) of the 20S proteasome (8QYM) by CDOCKER. Data (left) are mean ± SD, of top 10 poses. (B) CPD57 ligand affinity chromatography (AP:C57) with brain protein lysates of 6-month-old B6 mice. CPD57-interacting PSMA3 (lanes 1 and 2) was detected in immunoblot (IB). Lanes 3 and 4 are controls from a control affinity matrix (AP: Con.). Two biological replicates are shown (1,3; 2,4). (C) Nano-LC-ESI-MS/MS identification of PSMA3 as a CPD57-interacting protein in brain lysates of B6 mice. (D) Brain contents of CPD57 (left) and brain-to-serum ratios (right) of 18-month-old Tg2576 mice after 6 months of treatment with the indicated doses of C57. Data are mean ± SD (*n* = 6 mice per group); one-way ANOVA and Tukey’s test; F(3,20) = 16.93 (left); F(2,15) = 2.695 (right). (E) Enhanced proteasome activity in hippocampi of 18-month-old Tg2576 (AD) mice after 14 days of treatment with CPD57 (AD+57; 10 mg/kg/d). As indicated, the 20S proteasome was activated by SDS (0.03%, +). Proteasome activity is presented as % of the untreated AD control (mean ± SD; *n* = 4 mice per group); one-way ANOVA and Tukey’s test; F(3,12) = 38.09. (F) Immunoblot (IB) of PSMA3 in hippocampal, cytosolic proteins of 18-month-old Tg2576 mice without treatment (AD) and with 14 days of treatment with CPD57 (+57). The right panel shows quantitative immunoblot data (df = 6; t = 12.14). (G and H) Immunoblot (IB) of phospho-S670-GRK2 (G, left) and GRK2 with GRK2-specific antibodies against the N-terminus of GRK2 (G, right) in hippocampal cytosolic proteins of 18-month-old Tg2576 AD mice without treatment (AD) and with 14 days of treatment with CPD57 (+57). The lower control blot detects ADSL. Bar graphs (H) show quantitative data (df = 6; t = 14.45, 5.535). (I and J) Immunoblot (IB) of phospho-S670-GRK2 (I, left) and GRK2 with GRK2-specific antibodies against the N-terminus (I, right) on hippocampal mitochondria of 18-month-old Tg2576 mice without treatment (AD) and with 14 days of treatment with CPD57 (+57). The control blot detects TOMM40. Bar graphs (J) show quantitative data (df = 6; t = 2.805, 2.858; 5.266, 12.40). (K) Immunoblot (IB) of TOMM6 on hippocampal mitochondria of 18-month-old Tg2576 mice without treatment (AD) and with 14 days of treatment with CPD57 (+57). Bar graphs (right panels) show quantitative immunoblot data of aggregated (aggr.) and monomeric (mono.) TOMM6 (df = 6; t = 6.417, 9.231). (F, H, J, and K) Data represent mean ± SD; *n* = 4 mice per group (F and H) and *n* = 4 biological replicates per group (J and K); unpaired, two-tailed *t* test. (L) Immunohistological detection of hippocampal Aβ plaques (brown) on coronal brain sections of 18-month-old Tg2576 male (m) mice without treatment (AD) and with 6 months of CPD57 treatment (+57; 10 mg/kg/d). Nuclei were stained with hematoxylin (HE); scale bar: 200 μm. Immunohistology is representative of six mice per group. Replicates and quantitative data are shown in Figures S5A and S5B. (M and N) Insoluble (M) and soluble (N) Aβ1-40 (upper) and Aβ1-42 (lower) in brains of 18-month-old Tg2576 mice without (AD) and with 6 months of CPD57 treatment (+57). Data are mean ± SD (*n* = 6 mice per group); unpaired, two-tailed *t* test; df = 10; t = 12.73, 13.34 (M); 4.822, 3.117 (N). (O) Proteasome activities of brains from 18-month-old Tg2576 (AD) mice after 6 months of treatment with the indicated doses of CPD57. (P) Contents of insoluble Aβ1-40 in brains of 18-month-old Tg2576 (AD) mice after 6 months of treatment with the indicated doses of CPD57. (O and P) Data are mean ± SD (*n* = 6 mice per group); one-way ANOVA and Tukey’s test; F(3,20) = 34.26 (Suc-LLVY-AMC), 32.49 (Boc-LRR-AMC), 27.87 (Z-LLE-AMC), 40.90 (P). See also Figure S5.

Normally, phosphorylation of GRK2 at serine-670 enhances its degradation by the proteasome, which is required for phospho-S670-GRK2-dependent cell-cycle progression.^18^^,^^41^ In contrast, in AD brains, there was extensive accumulation of aggregated phospho-S670-GRK2, which could be at least in part caused by the proteasome dysfunction in AD.^42^ We aimed to stimulate the degradation of aggregated phospho-S670-GRK2 with a proteasome enhancer. Proteasome enhancers modulate proteasome assembly and act as gate openers by interacting with alpha-subunits of the 20S proteasome.^43^ By molecular docking of drug candidates to the 20S proteasome gate,^44^ we identified CPD57, 4-(4-fluorophenyl)-6-(hydroxymethyl)-2-methyl-pyridine-3-carboxamide, as a proteasome-binding small molecule (Figure 7A). CPD57 was docked with a highly favorable binding energy of −511.5 kcal/mol to a binding site at the interface between PSMA3 and PSMA1 (proteasome subunit alpha type-3 and type-1) of the 20S proteasome pore (Figure 7A).

Ligand affinity chromatography showed that CPD57 interacted with PSMA3 in brain protein extracts from B6 mice (Figure 7B). The identity of the CPD57-enriched PSMA3 was confirmed by nano-LC-ESI-MS/MS (Figure 7C). CPD57 had a good brain permeability with a brain-to-serum ratio of 0.84 ± 0.14 (Figure 7D).

PSMA3 facilitates the degradation of intrinsically disordered proteins and interacts with MDM2, which is an E3 ubiquitin ligase targeting phospho-S670-GRK2 for degradation.^41^^,^^45^ Treatment of 18-month-old Tg2576 mice for 14 days with the PSMA3-interacting CPD57 (10 mg/kg/d) significantly augmented the hippocampal 20S proteasome activity and the hippocampal PSMA3 (Figures 7E and 7F). Concomitantly, CPD57 enhanced the degradation of mitochondrial phospho-S670-GRK2 aggregates while non-phosphorylated GRK2 monomers increased (Figures 7G–7J). With degradation of phospho-S670-GRK2 (aggregates), TOMM6 protein aggregates were also significantly reduced (Figure 7K).

Long-term treatment for 6 months largely prevented hippocampal Aβ plaques, mitochondrial Aβ, and insoluble and soluble Aβ in brains of 18-month-old Tg2576 mice (Figures 7L–7P, S5A–S5C, and S5E). The senescence marker, UPAR, was also significantly reduced by CPD57 (Figures S5D and S5F). Effects of CPD57 on the three different proteasome activities and on Aβ were dose-dependent (Figures 7O and 7P).

## Discussion

This study found increased aggregated serine-670-phosphorylated GRK2 (phospho-S670-GRK2) in brains of Tg2576 AD mice and patients with dementia likely due to AD. The two AD hallmark proteins, Aβ and PHF-tau, triggered phospho-S670-GRK2 aggregation, most likely because of mitochondrial dysfunction, oxidative stress, and sustained ERK1/ERK2 activation.^26^^,^^46^ Aggregated phospho-S670-GRK2 could further accumulate due to the decreased brain PPP2CA activity of AD brains^24^ and the proteasome dysfunction present in AD.^42^

Aggregated phospho-S670-GRK2 could be pathological because it trapped its kinase substrate, TOMM6, and promoted TOMM6 aggregation by not phosphorylating TOMM6. Aggregated TOMM6 was also dysfunctional because it did not interact with the pore-forming TOMM40 of the TOMM complex. Impaired TOMM6 function could further enhance AD-induced mitochondrial degeneration and aging because loss of *Tomm6* causes a phenotype of premature aging with an early death at an age of 4–5 weeks^32^

To inhibit dysfunctional GRK2 aggregation, we investigated the effect of ARRB1, which only partially ameliorated the AD-related neuropathology. On one hand, moderately increased ARRB1 in Tg2576-*ARRB1* mice improved the mitochondrial function, reduced TOMM6 and GRK2 aggregation, and decreased Aβ plaques. This could be due to an improved mitochondrial function caused by ARRB1-enhanced mitophagy and reduced mitochondrial fission.^47^^,^^48^ On the other hand, increased ARRB1 elevated the γ-secretase activity and accelerated soluble Aβ peptide accumulation, through interaction with APH1A.^31^

Replenishment of TOMM6 by neuron-specific TOMM6 expression also inhibited aggregated phospho-S670-GRK2 and aggregated TOMM6 and prevented the ROS-dependent protein aggregation and Aβ plaque formation but left the hippocampal phospho-S670-GRK2 untouched. Consequently, amyloidogenic APP processing was unaltered, soluble neurotoxic Aβ peptides accumulated, and the mortality of Tg2576-*TOMM6* mice was increased. Soluble Aβ (oligomers) are neurotoxic and enhance the mortality of Tg2576 mice by causing epileptic seizures.^29^ Moreover, the increased TOMM6 could promote the co-assembly of TOM complex components, which enhances neuronal degeneration.^49^

To reduce phospho-S670-GRK2 and TOMM6 aggregates simultaneously, we developed CPD10 as a GRK2 function modulator (Figure 5I). Inhibition of phospho-S670-GRK2 aggregation and reconstitution of the equilibrium between non-phosphorylated and phospho-S670-GRK2 by CPD10 could exert several neuroprotective activities. (I) By phosphorylation of TOMM6, the reconstituted GRK2 could prevent TOMM6 aggregation and mitochondrial dysfunction. (II) By enhancing cholinergic neurotransmission,^4^ and by promoting phosphorylation of APH1A^30^ and GSK3B,^40^ GRK2 could enhance the non-amyloidogenic processing of APP and prevent PHF-tau, neurodegeneration, and neuronal loss. (III) By reduction of neurotoxic, soluble Aβ and by reconstitution of the anti-aging TOMM6, GRK2 function modulation could decrease the senescence marker, UPAR,^50^^,^^51^ reduce the AD-related mortality, and prolong survival. (IV) By phospho-S670-GRK2 inhibition, CPD10 could exert neuroprotection by inhibiting HDAC6 (histone deacetylase 6),^19^^,^^52^ and counteract age-related cardiac dysfunction.^16^^,^^17^ (V) By phospho-S670-GRK2 (aggregate) degradation, the GRK2 function modulator could restore the physiological equilibrium between non-phosphorylated and phospho-S670-GRK2, which is required for cell survival and cell-cycle progression.^18^^,^^41^ Thereby, CDP10 could promote neuronal survival and neurogenesis.

The herein deduced neuroprotective and anti-aging activities of GRK2 reconstitution by small molecules are supported by previous studies, which showed the neuroprotective and anti-aging potential of GRK2 in mice.^4^^,^^5^^,^^6^^,^^53^ The study could also be relevant for the human disease because we detected aggregated phospho-S670-GRK2 on cortex biopsy specimens from human patients with dementia likely due to AD. A limitation of the study is the small number of human patient specimens. However, human brain specimens were taken during surgery and not postmortem (Figure S9; Table S3), which could facilitate the detection of phospho-S670-GRK2. With the additional caveat that treatment data need to be translated to human patients, this study identified pathological, serine-670-phosphorylated GRK2 aggregates as a neurodegenerative factor that can be targeted by small molecules in a well-tolerated treatment approach for several AD- and age-related pathologies.

### Limitations of the study

This study found increased aggregated p-S670-GRK2 in Alzheimer brains. Aggregation of p-S670-GRK2 enhanced AD pathologies and mitochondrial dysfunction. A limitation of the study is the open question whether the aggregated phospho-S670-GRK2 protein on mitochondria is the main neuropathologic factor or whether the reduction of functional GRK2 in the cell, which is caused by GRK2 aggregation, is another major driver of neuropathologic features and beta-amyloid generation. Studies with GRK2-deficient organisms are needed to address this important question.

## Resource availability

### Lead contact

Further information and requests for resources and reagents should be directed to and will be fulfilled by the lead contact, Ursula Quitterer (ursula.quitterer@pharma.ethz.ch).

### Materials availability

• Sperm of transgenic mouse lines generated in this study were cryopreserved by mouse repositories.
• CPD10, NH2-CPD10, and CPD57 generated in this study will be made available on request, but we may require a payment and/or a completed Materials Transfer Agreement if there is potential for commercial application.
• All other unique/stable reagents generated in this study are available from the lead contact without restriction.

### Data and code availability

• Whole-genome microarray gene expression data have been deposited to NCBI GEO: GSE317145 (https://www.ncbi.nlm.nih.gov/geo/query/acc.cgi?acc=GSE317145), GSE318045 (https://www.ncbi.nlm.nih.gov/geo/query/acc.cgi?acc=GSE318045), GSE318046 (https://www.ncbi.nlm.nih.gov/geo/query/acc.cgi?acc=GSE318046), and GSE318047 (https://www.ncbi.nlm.nih.gov/geo/query/acc.cgi?acc=GSE318047. Mass spectrometry proteomics data are available via ProteomeXchange: PXD073643 (https://doi.org/10.6019/PXD073643) and PXD073574 (https://doi.org/10.6019/PXD073574).
• Data analyzed and reported have been deposited in the online repository ETH Zurich Research Collection (https://doi.org/10.3929/ethz-c-000795228).
• No custom code was generated in this study.
• Any additional information required to reanalyze the data reported in this paper is available from the lead contact upon request.

## Acknowledgments

We thank Dr. S. AbdAlla (ETH Zurich) for mouse phenotyping and treatments, and Dr. A. El Missiery (MRC Cairo) for research support. This work was supported in part by grants ETH-18 14-2, SNSF140679, and SNSF169354 (to U.Q.).

## Author contributions

J.A.A., A.P., A.L., X.F., Y.F., and U.Q. conducted the experiments; J.A.A. performed molecular modeling, MD simulations, and *in silico* docking studies; J.A.A. and U.Q. designed the experiments and wrote the paper. All authors read and approved the final version of the manuscript.

## Declaration of interests

CPD10 and CPD57 are covered by ETH Zurich patent applications.

## STAR★Methods

### Key resources table

**Table:** 

REAGENT or RESOURCE	SOURCE	IDENTIFIER
**Antibodies**		

Mouse monoclonal anti-GRK2 antibody (C-9); Lot E0917; Lot G1410, Lot I1623, Lot L1119	Santa Cruz Biotechnology	Cat. No. sc-13143; RRID:AB_626751
Rabbit polyclonal anti-ADRBK1/GRK2 antibodies against the N-terminus of GRK2; Lot WOVUS 31186	LSBio	LS-C358899
Rabbit polyclonal anti-GRK2 antibodies	Fu et al.^27^; Abd Alla et al.^28^	N/A
Rabbit polyclonal anti-phospho-GRK2 (serine-670) antibodies; Lot ZH4432180D, ZI448570A	Invitrogen	Cat. No. PA5-77851; RRID:AB_2736379
Rabbit polyclonal anti-phospho-GRK2 (serine-670) antibodies	This paper	N/A
Rabbit monoclonal anti-ARRB1 antibody (Clone D803J); Lot 3	Cell Signaling Technology	Cat. No. 12697S RRID:AB_2797996
Mouse monoclonal anti-ARRB1 antibody (Clone 1H8E4); Lot 10015333	Proteintech	Cat. No. 67580-1-Ig
Rabbit polyclonal anti-APH1A antibodies Picoband® against the C-terminus of human APH1A, HRP-conjugated; Lot 25XD703K03	Boster Biological Technology	Cat. No. PB9421-HRP
Rabbit polyclonal anti-APH1A antibodies; Lot 00002255	Proteintech	Cat. No. 11643-1
Mouse monoclonal anti-Gao (GNAO) antibody (Clone A2); Lot A1005	Santa Cruz Biotechnology	Cat. No. sc-13532; RRID:AB_2111645
Mouse monoclonal anti-calcineurin (α-subunit) (PPP3CA) antibody (Clone CN-A1); Lot 094M4820V	Invitrogen; Sigma-Aldrich	Cat. No. C1956; RRID:AB_258774
Rabbit polyclonal anti-ADSL antibodies; Lot A118656	Invitrogen; ThermoFisher Scientific	Cat. No. PA5-51401
Rabbit polyclonal anti-ADSL antibodies; Lot AC46454371	Invitrogen; ThermoFisher Scientific	Cat. No. PA5-92861
Mouse monoclonal anti-ATP5A antibody (clone 15H4C4); Lot 21010533059	Abcam	Cat. No. ab14748; RRID:AB_301447
Rabbit polyclonal anti-TOMM6 antibodies; Lot 000044616, Lot R07460, Lot A105468	Sigma Prestige Antibodies	Cat. No. HPA004801; RRID:AB_1859553
Rabbit polyclonal anti-TOMM6 antibodies	This paper	N/A
Rabbit polyclonal anti-TOMM40 antibodies; Lot 00105350, Lot 00040008	Proteintech	Cat. No. 18409-1-AP; RRID:AB_2303725
Mouse monoclonal anti-ERK1 and ERK2 antibody (clone ERK-7D8); Lot GR208721-6	Abcam	Cat. No. ab54230 RRID:AB_2139967
Mouse monoclonal anti-Phosphoserine antibody (Clone 4A4); Lot 3754811, Lot 4290029	EMD Millipore Corp.	Cat. No. 05-1000
Mouse monoclonal anti-beta-amyloid antibody (clone BAM-10); ascites fluid; Lot # 103M4762; Lot 037K4877; Lot 087M4874V; Source # 0000127507 with Batch #0000132482; Batch number 062M4782	Sigma-Aldrich	Cat. No. A5213; RRID:AB_476742
Mouse monoclonal anti-human PHF-Tau (Ser202, Thr205) antibody (clone AT8); Lot HG105303; Lot IB112040; Lot TL26525315	Pierce Biotechnology, Invitrogen	Cat. No. MN1020; RRID:AB_223647
Rabbit recombinant monoclonal TAU antibody [EP2456Y]; Lot GR3402049-7	Abcam	Cat. No. ab76128; RRID:AB_1524475
Mouse monoclonal anti-Neuronal Nuclei (NeuN) antibody; clone A60; Lot LV1388597	Upstate/Chemicon	Cat. No. MAB377; RRID:AB_2298772
Rabbit monoclonal anti-SNAP25 antibody [EP3274]; Lot 1018041-12	Abcam	Cat. No. ab108990; RRID:AB_10888111
Mouse monoclonal anti-GSK3B antibody; clone 1F7; Lot B0117, Lot D1718	Santa Cruz Biotechnology	Cat. No. sc-53931; RRID:AB_783601
Mouse monoclonal anti-phospho-GSK-3b (Ser9) antibody; clone F-2; Lot G2219, Lot S2217	Santa Cruz Biotechnology	Cat. No. sc-373800; RRID:AB_10920410
Rabbit polyclonal anti-PPP2CA antibodies; Lot 00023021	Proteintech	Cat. No. 13482-1-AP; RRID:AB_2169485
Rabbit recombinant monoclonal anti-PSMA3 antibody [EPR5455]; Lot 1003633-7	Abcam	Cat. No. ab109532; RRID:AB_10862353
Mouse monoclonal anti-UPAR antibody (Clone E−3; Lot IO817)	Santa Cruz Biotechnology	Cat. No. sc-376494; RRID:AB_11150125
Goat Anti-Mouse IgG, Peroxidase-conjugated AffiniPure F(ab’)2 Fragment, Fcgamma Fragment Specific, (minimal cross-reaction to Human, Bovine, and Horse Serum Proteins); Lot 77832, Lot 118230, Lot 151768	Jackson ImmunoResearch Laboratories	Cat. No. 115-036-071; RRID:AB_2338524
Goat Anti-Rabbit IgG, Peroxidase-conjugated AffiniPure F(ab’)2 Fragment, Fcgamma Fragment Specific, (minimal cross-rection to Human Serum Proteins); Lot 84989, Lot 113992	Jackson ImmunoResearch Laboratories	Cat. No. 111-036-046; RRID:AB_2337944
Alexa Fluor™ 488 goat anti-Mouse IgG (H + L) Highly Cross-Adsorbed Secondary Antibody; Lot 57465A	Invitrogen; Molecular Probes	Cat. No. A-11029; RRID:AB_2534088
Alexa Fluor™ 568 goat anti-Rabbit IgG (H + L); Lot 1811756	Invitrogen by Thermo Fisher Scientific	Cat. No. A-11011; RRID:AB_143157

**Bacterial and virus strains**		

BL21(DE3)pLysS competent bacteria Novagen®	Merck	Cat. No. 69451

**Biological samples**		

Human cerebral cortex specimens of 38 glioblastoma patients. Four patients were diagnosed with dementia likely due to AD and glioblastoma. See Figure S9 and Table S3 for details.	MRC (Medical Research Center), Faculty of Medicine, Cairo, Egypt	Ain Shams University Hospitals, Cairo, Egypt

**Chemicals, peptides, and recombinant proteins**		

Urea electrophoresis purity reagent	Bio-Rad	Cat. No. 1610730
SDS (Sodium Dodecyl Sulfate) electrophoresis purity reagent	Bio-Rad	Cat. No. 1610302
Dithiothreitol (DTT)	Bio-Rad	Cat. No. 161-610
Iodoacetamide	Bio-Rad	Cat. No. 1632109
Coomassie® Brilliant Blue R 250	Serva	17525
Aqua-Poly/Mount	Polysciences, Inc.	Cat. No. 1860620
Poly-Mount Xylene	Polysciences, Inc.	Cat. No. 24176120
Mayer’s hemalum solution	Merck KGaA	Cat. No. 1.09249.0500
DAPI solution (1 mg/mL)	Thermo Fisher Scientific	Cat. No. 62248
Amicon® Ultra Centrifugal Filter, 3kDa MWCO	Merck Millipore; Sigma-Aldrich	Cat. No. UFC8003
Protease Inhibitor Cocktail	Sigma-Aldrich	Cat. No. P8340
Protein phosphatase inhibitor cocktail	Sigma-Aldrich	Cat. No. PPC1010
Ni-NTA Agarose	Qiagen	Cat. No. 30210
PD-10 desalting columns packed with Sephadex G-25 resin	GE Healthcare GmbH	Cat. No. 17-0851-01
Sephadex™ LH-20	Cytiva	Cat. No. 17-0090-01
Zeba™ spin desalting columns (7K MWCO)	Thermo Fisher Scientific	Cat. No. 89889
Pierce™ Iodination Reagent (1,3,4,6-tetrachloro-3α,6α-diphenyl-glycoluril) (Iodo-Gen®)	Pierce; Thermo Fisher Scientific	Prod. 28600
Percoll™	GE Healthcare GmbH	Cat. No. GE17-0891-01
B-27 Supplement	Gibco; Thermo Fisher Scientific	Cat. No. 17504-044
Neurobasal medium	Gibco; Thermo Fisher Scientific	Cat. No. 21103049
TC-100 Insect Medium	Gibco; Thermo Fisher Scientific	Cat. No. 13055025
1-(1,3-benzodioxol-5-yl)-4-(cyclopropanecarbonyl)-3-hydroxy-2-phenyl-2H-pyrrol-5-one (CPD10)	This paper (synthesized by ChiroBlock GmbH, 06766 Bitterfeld-Wolfen, Germany	N/A
2-(4-aminophenyl)-1-(1,3-benzodioxol-5-yl)-4-(cyclopropanecarbonyl)-3-hydroxy-2H-pyrrol-5-one (NH2-CPD10)	This paper (synthesized by ChiroBlock GmbH, 06766 Bitterfeld-Wolfen, Germany	N/A
4-(4-fluorophenyl)-6-(hydroxymethyl)-2-methyl-pyridine-3-carboxamide (CPD57)	This paper (synthesized by ChiroBlock GmbH, 06766 Bitterfeld-Wolfen, Germany	N/A
CMPD101 hydrochloride	Sigma-Aldrich	Cat. No. SML2784
Affigel-10	Bio-Rad	Cat. No. 1536099
Epoxy-activated Sepharose 6B	Cytiva	Cat. No. 17048001
Alexa Fluor™ 647 NHS Ester (Succinimidyl Ester) (Invitrogen™)	Thermo Fisher Scientific	Cat. No. A37573
Fluorogenic α-secretase substrate Ac-RE(EDANS)-VHHQKLVF-K(DABCYL)-R-OH	Calbiochem; Merck	Cat. No. 565767
Fluorogenic β-secretase substrate H-K(DABSYL)-SEVNLDAEFRQ(LY)	Calbiochem; Merck	Cat. No. 565781
Fluorogenic γ-secretase substrate NMA-GGVVIATVK(DNP)-DRDRDR-NH_2_	Calbiochem; Merck	Cat. No. 565764
Protein A, Peroxidase Conjugate	Calbiochem; Merck; Millipore	Cat. No. 539253
Protein G, HRP conjugate	EMD Millipore Corporation	Catalog #18-161
Syn-PER™ Reagent	Thermo Fisher Scientific	Cat. No. 87793
RNAlater®	Qiagen	Cat. No. 1017980
Amyloid-beta (1–42) peptide trifluoroacetate salt	Cayman Chemical	Cay20574-1
Recombinant His_6_-tagged human GRK3	Eurofins DiscoverX	Cat. No. 15-028
Recombinant His_6_-tagged human GRK6	Eurofins DiscoverX	Cat. No. 14-715
ATP, [γ-^32^P], specific activity 10 Ci/mmol	PerkinElmer	BLU002001MC
Na[^125^I], specific activity 17.4 Ci/mg	PerkinElmer	NEZ033A001MC

**Critical commercial assays**		

RNeasy Mini kit	Qiagen	Cat. No. 74104
DAB Enhanced Liquid Substrate System	Sigma-Aldrich	Cat. No. D3939
Amersham ECL Prime Western Blotting Detection Reagent	Cytiva	RPN2232
Pierce™ ECL Plus Western Blotting Substrate	Thermo Fisher Scientific	Cat. No. 32132
Bac-To-Bac™ Baculovirus Expression System	Thermo Fisher Scientific	Cat. No. 10359016
GeneChip Human Genome U133 Plus 2.0 Array Affymetrix	Affymetrix	Cat. No. 900466
GeneChip Mouse Genome 430 2.0 Array	Affymetrix	Cat. No. 900497
Proteasome 20S Assay Kit	Enzo Life Sciences	BML-AK740
Proteasome Activity Fluorometric Kit II	UBPBio	Cat. J4120
Aconitase Assay Kit	Cayman Chemical	Item No. 705502
ATP determination kit	Thermo Fisher Scientific	Cat. No. A22066
Human Amyloid beta 40 ELISA kit	Thermo Fisher Scientific	KHB3481
Human amyloid beta 42 ELISA kit	Thermo Fisher Scientific	KHB3441
Fluidity one-W disposable chips	Fluidic Analytics Ltd., Cambridge, UK	Item number: 100009

**Deposited data**		

Whole transcriptome microarray gene expression data of human cerebral cortex specimens of 38 glioblastoma patients including 4 patients with dementia likely due to AD.	This paper	NCBI GEO database: GSE318045 (https://www.ncbi.nlm.nih.gov/geo/query/acc.cgi?acc=GSE318045); GSE318046 (https://www.ncbi.nlm.nih.gov/geo/query/acc.cgi?acc=GSE318046); GSE318047 (https://www.ncbi.nlm.nih.gov/geo/query/acc.cgi?acc=GSE318047)
Whole transcriptome microarray gene expression data of 18-month-old Tg2576 mouse frontal cortex specimens without and with 6 months of treatment with CPD10	This paper	NCBI GEO database: (https://www.ncbi.nlm.nih.gov/geo/query/acc.cgi?acc=GSE317145)
TOMM6 identification by nano-LC-ESI-MS/MS analysis	This paper	ProteomeXchange dataset identifier: PXD073643 (https://doi.org/10.6019/PXD073643)
PSMA3 identification by nano-LC-ESI-MS/MS analysis	This paper	ProteomeXchange dataset identifier: PXD073574 (https://doi.org/10.6019/PXD073574)-
Data analyzed and reported	This paper	Online repository ETH Zurich Research Collection: https://doi.org/10.3929/ethz-c-000795228
Alphafold model of mitochondrial import receptor subunit TOM6 homolog	https://alphafold.ebi.ac.uk/entry/Q96B49	AF-Q96B49-F1-v4
Atomic structure of human TOM core complex	Wang et al.^25^	PDB ID: 7CK6
20S proteasome assembly structure	Adolf et al.^44^	PDB ID: 8QYM
Human GRK2 in Complex with Gbetagamma subunits	Tesmer et al.^35^	PDB ID: 3CIK
Bovine GRK2 tetramer	Lodowski et al.^37^	PDB ID: 1YM7

**Experimental models: Cell lines**		

Primary fetal mouse cortical neurons	This paper	N/A
*Sf9* Cell Line from *Spodoptera frugiperda* pupal ovarian tissue	Sigma-Aldrich	89070101-1VL

**Experimental models: Organisms/strains**		

Mouse: B6 (C57BL/6J)	Charles River	JAX Mice Stock Number 000664
Mouse: Tg(APPSWE2576Kha)	Taconic Biosciences	Model 1349
Mouse: Tg(APPSWE)2576Kha-Tg(Prnp-MAPT∗P301L)JNPL3Hlmc	Taconic Biosciences	Model 2469
Mouse: FVB/N-Tg(CosHa*TOMM6*) 4 Sjaa	This paper	JAX strain ID 419255
Mouse: FVB/N-Tg(CMV*GRK2*) 3 Sjaa	This paper	JAX strain ID 419257
Mouse: FVB/N-Tg(CMV*GRK2S670D*) Sjaa	This paper	Janvier Labs ID 202.586 LASC Pharmakologie
Mouse: C57BL/6Tg (CMV*GRK2K220R*) 1 Sjaa	This paper	JAX strain ID 911824
Mouse: C57BL/6Tg(CMV*GRKInh*) 1 Sjaa	Fu et al.^27^	JAX strain ID 911820
Mouse: FVB/N-Tg(CMV*GRKInh*) 4 Sjaa	Fu et al.^27^	JAX strain ID 419256
Mouse: C57BL/6-Tg(CMV*ARRB1*) 1 Sjaa	Quitterer et al.^54^	Janvier Labs ID 181.077 and 182.534 ETH Zurich

**Oligonucleotides**		

See Table S4 for oligonucleotides.	This paper	N/A

**Recombinant DNA**		

Plasmid: pFastBac1	ThermoFisher Scientific	Cat. No. 10360014
Plasmid: pFastBac1-His_6_-GRK2	This paper	N/A
Plasmid: pFastBac1-His_6_-GRK2S670A	This paper	N/A
Plasmid: PET-3d expression plasmid Novagen	Merck	Cat. No. 69421-3
Plasmid: PET-3d-His_6_-TOMM6	This paper	N/A
Plasmid: PET-3d-His_6_-TOMM6-ST-AA	This paper	N/A
Plasmid: pET-3d-His_6_-ERK1	This paper	N/A
Plasmid: pcDNA3.1 Invitrogen™	Thermo Fisher Scientific	Cat. No. V79020
Plasmid: pcDNA3.1-GRK2	Fu et al.^27^	N/A
Plasmid: pcDNA3.1-GRK2-S670D	This paper	N/A
Plasmid: pcDNA3.1-GRK2-K220R	Fu et al.^27^	N/A
Plasmid: pcDNA3.1-GRKInh	Fu et al.^27^	N/A
Plasmid: pcDNA3.1-ARRB1	Quitterer et al.^54^	N/A
Plasmid: pLenti4/V5-DEST Invitrogen™	Thermo Fisher Scientific	Cat. No. V49810
Plasmid: pLenti4TOMM6-ST-DD	This paper	N/A
Plasmid: pLenti4TOMM6	This paper	N/A
Plasmid: CosSHa.Tet Vector	InPro Biotechnology	EXV-COST
Plasmid: CosSHa.Tet-TOMM6	This paper	N/A
I.M.A.G.E. Fully Sequenced cDNA Clone TOMM6, Locus accession number BC015975; cDNA clone IMAGE: 4133975	Source BioScience Clones	IRAUp969E0270D

**Software and algorithms**		

Biovia Discovery Studio Studio 2024 (v24.1.0.23298)	Dassault Systèmes Biovia Corp.	https://www.3ds.com/de/products/biovia/discovery-studio
GraphPad Prism Version 10.4.1	GraphPad	https://www.graphpad.com/updates/prism-10-4-0-release-notes
R for macOS	R Foundation	https://stat.ethz.ch/CRAN/
Fiji/ImageJ ImageJ 1.54p	Fiji/ImageJ	https://imagej.net/software/fiji/downloads
g:GOSt of g:Profiler (g:Profiler version e113_e.g.,59_p19_f6a03c19, database updated on 23/05/2025)	ELIXIR Estonia	https://biit.cs.ut.ee/gprofiler/gost
Morpheus	Broad Institute	https://software.broadinstitute.org/morpheus
MS/MS Ions Search, Mascot version 3.0.0	Matrix Science	https://www.matrixscience.com/server.html
GeneChip ® Operating Software (GCOS) v1.2	Affymetrix	N/A
Hydrodynamic radius converter	Fluidic Sciences	https://fluidic.com/molecular-weight-to-hydrodynamic-radius-converter/
Adobe Illustrator 2026, version 30.0	Adobe Systems Software Ireland Limited	https://www.adobe.com/ch_de/creativecloud/plans.html
The table does not report custom code		

**Other**		

SSBK-Z′-LYTE™ Assay Thermo Fisher Scientific’s SelectScreen™ Profiling Service	Thermo Fisher	N/A
SSBK-ADAPTA Assay Thermo Fisher Scientific’s SelectScreen™ Profiling Service	Thermo Fisher	N/A
Target profiling service of CPD10	Eurofins Discovery	N/A

### Experimental animals, cells, and human subjects details

#### Cell culture

Primary cortical neurons were isolated from dissected cortical tissue of brains from fetal male and female B6 mice (embryonic age: E16-E19). Primary cortical neurons were kept in Neurobasal medium supplemented with 1% heat-inactivated horse serum, 2% B-27 Supplement (Gibco, Thermo Fisher Scientific), 0.5 mM glutamine, 33 mM glucose, and 1% penicillin-streptomycin for 72 h. After 72 h of *in vitro* culture in a humidified atmosphere at 37°C with 5% CO_2_, cells were transferred to serum-free Neurobasal medium, and 50% of the medium were replaced every 3–4 days.

The Sf9 insect cell line from *Spodoptera frugiperda* pupal ovarian tissue (89070101-1VL, Sigma-Aldrich) was grown (culture type: adherent) in TC-100 medium supplemented with 2 mM glutamine and 10% FCS in a humidified atmosphere at 27°C with ambient CO_2_.

All cells were negative for mycoplasma contamination.

#### Animals

The study used B6;SJL-Tg(APPSWE)2576Kha (Tg2576) mice, as a model of familial AD (Model 1349, Taconic Biosciences). The impact of the two major hallmark proteins of AD, amyloid-beta (Aβ) and the fibrillary-tangle-inducing TAU-P301L (MAPT∗P301L), on serine-670 phosphorylation of GRK2, was investigated with Tg2576-*MAPT∗P301L* mice (Tg(APPSWE)2576Kha-Tg(Prnp-MAPT∗P301L)JNPL3Hlmc; Model No. 2469, Taconic Biosciences). Tg2576 and Tg2576-MAPT∗P301L mice with B6 (C57BL/6J) background were obtained by backcrossing. Histologically detectable Aβ plaques start in Tg2576 AD mice at an age of 12-month and continue to accumulate until the end of the observation period at 18 months^21^^,^^22^ To determine Aβ plaque accumulation, the study compared groups of single-transgenic Tg2576 mice (age groups of 12-, 16-, and 18-month-old, female and male mice) with age-matched, double-transgenic, female and male Tg2576-CMV*GRK2*, Tg2576-CMV*GRK2S670D*, Tg2576-CMV*GRK2K220R*, Tg2576-CMV*GRKInh*, Tg2576-CMV*ARRB1*, Tg2576-CosSHa*TOMM6* and Tg2576-*MAPT∗P301L* mice. The double-transgenic mouse lines were generated by crossbreeding of male Tg2576 mice with the respective female, homozygous, single-transgenic mice. The single-transgenic mouse lines were generated in frame of the study (see method below). The presence of both transgenes was verified by PCR genotyping of ear-punch biopsies. As control groups, 6-month-old, 12-month-old and 18-month-old non-transgenic B6 mice (female and male) were also included in the study. After reaching the indicated age, mice were anesthetized (ketamine/xylazine 100/10 mg/kg; i.p.) under temperature control to avoid hypothermia, intracardially perfused with PBS, and brains were rapidly isolated on ice.

Female and male Tg2576 mice were used for the study. All mice were housed in groups of 2–3 animals under specified pathogen-free conditions with a 12 h light/12 h dark cycle and humidity and temperature control. If aggressive, male mice were single housed with environmental enrichment. Mice with (healed) bite wounds were excluded. All mice had free access to food and water *ad libitum*, unless the CUMS protocol required a restriction. Groups of sex-matched littermates were assigned to treatment and control groups by the researcher based on genotype. All animal experiments were performed according to NIH and Swiss guidelines and were approved by the local committees on animal research (Cantonal Veterinary Office Zurich; 126/2009 approval 04.08.2009; 145-G, approval 14.02.2013; ZH014/2023 approval 12.04.2023; Medical Research Center, Ain Shams University, Cairo; approval 02.01.2007).

#### Human samples

The study analyzed cerebral cortex biopsy specimens from 38 patients, which were obtained in frame of surgery for glioblastoma (Table S3). Four of these patients had dementia with the clinical diagnosis of probable Alzheimer’s disease (AD) according to the NINCDS-ADRDA criteria (f/m: 3/1; age 66.3 ± 4.9 years [mean ± s.d.]), and four gender and age-matched patients with glioblastoma but without dementia (f/m: 3/1; age 66.3 ± 5.9 years [mean ± s.d.]) served as the comparison group for protein studies (Table S3). It is well established that histopathological AD symptoms are present in a substantial proportion of glioblastoma patients.^55^ To confirm the diagnosis of probable AD in the four patients with dementia on transcriptome level, whole-genome microarray gene expression profiling was performed. The whole genome gene expression profiling study included the cerebral cortex biopsy specimens from the four patients with dementia and additional cerebral cortex specimens from 34 patients without dementia. Thus, specimens of 38 glioblastoma patients (31 females, 7 males; age 59.2 ± 9.6 years [mean ± s.d.]; IDH wild-type) were analyzed. Cerebral cortex specimens were isolated from three groups of glioblastoma patients (Figure S9). Group-I includes cerebral cortex specimens from 10 glioblastoma patients which were collected and processed in 2007. Group-II includes cerebral cortex specimens from 16 glioblastoma patients which were isolated and processed in 2008. Group-III includes cerebral cortex specimens from 12 glioblastoma patients which were isolated and processed in 2012 (Figure S9). The whole-genome microarray gene expression profiling study documented that human brain specimens from glioblastoma patients with the clinical diagnosis of probable AD were characterized by significantly increased AD-related transcripts including the tau pathology-related *C3AR1*,^56^ the amyloid-beta-related *OLR1*,^57^ and *BACE2*,^58^ compared to cerebral cortex specimens from patients without dementia (Figures S9A–S9C and S9E). Furthermore, cortex specimens from the four patients with dementia showed elevated insoluble Aβ contents in immunoblot analysis compared to four cerebral cortex specimens from age- and gender-matched patients without dementia (Figure S9D). Characteristics of all patients are shown in Table S3. Whole genome microarray gene expression data of all cerebral cortex specimens from three groups of glioblastoma patients (Group-I, Group-II, Group-III) have been deposited in the NCBI GEO database: GSE318045 (https://www.ncbi.nlm.nih.gov/geo/query/acc.cgi?acc=GSE318045), GSE318046 (https://www.ncbi.nlm.nih.gov/geo/query/acc.cgi?acc=GSE318046), and GSE318047 (https://www.ncbi.nlm.nih.gov/geo/query/acc.cgi?acc=GSE318047).

Written informed consent to use the biopsy specimens for research purposes was obtained from all patients or their guardians. The study was approved by the ethical committee of the Medical Research Center (MRC), Ain Shams University Hospitals, Cairo, Egypt.

### Method details

#### Animal models

This study used Tg2576 mice as a mouse model of AD (B6;SJL-Tg(APPSWE)2576Kha; Model No. 1349, Taconic Biosciences). These mice overexpress the Swedish double mutation of APP (695 amino acid isoform; APP_695_^K670N,M671L^; APPSWE) under control of the neuron-specific hamster prion protein promoter. To assess the impact of the two major hallmark proteins of AD, Aβ and fibrillary-tangle-inducing TAU-P301L, on serine-670-GRK2 phosphorylation, double-transgenic mice Tg(APPSWE)2576Kha-Tg(Prnp-MAPT∗P301L)JNPL3Hlmc (Model No. 2469, Taconic Biosciences) were used at an age of 12 months. Aβ plaques start to accumulate in Tg2576 mice at an age of 12 months, and substantial Aβ plaque load can be detected in brains of Tg2576 mice at an age of 18 months^21^^,^^22^ Therefore the progression of hippocampal Aβ plaque load was determined in groups of female and male mice at an age of 12 months, 16 months, and 18 months, as indicated. Oral treatment of Tg2576 mice with either CPD10 (2.7, 8, and 24 mg/kg/d in drinking water or by gavage) or CPD57 (3, 10, and 30 mg/kg/d by oral gavage) of Tg2576 mice was started at an age of 12 months and continued for 6 months until the age of 18 months. CPD57 at 10 mg/kg/d was also given for 14 days to a group of 18-month-old Tg2576 mice. Diffuse Aβ plaques start to accumulate in brains of Tg2576 mice at an age of 12 months,^21^^,^^22^ and treatment was started at this age. Formation of diffuse plaques also marks the Aβ deposition in human AD brains.^21^ The control groups consisted of age-matched and sex-matched, 18-month-old, untreated Tg2576 mice. To mimic environmental stress as a risk factor of AD, 14-month-old, male Tg2576 mice were subjected to the chronic unpredictable mild stress (CUMS) protocol,^22^ for eight weeks. According the CUMS protocol, the following stimuli were administered each week in random order, for eight weeks: two periods of 45° cage tilt (3 h); soaked cage for 12 h once a week; water deprivation (12 h), once a week; illumination for 9 h during the dark phase, once a week; noise (85 dB) in the room for 3 h, three times a week; flashing light (60 flashes/min) for 30 min, three times a week. The sucrose preference test (performed with a 1% sucrose solution in water) was performed immediately after a period of water deprivation. After eight weeks of stress, more than 90% of untreated Tg2576 mice showed a decrease in sucrose preference with ≤50% of sucrose consumption compared to that of non-stressed, age-matched Tg2576 mice. The sucrose preference of the CUMS-subjected Tg2576 group treated with compound CPD10 (8 mg/kg/d) during the CUMS protocol was not different from the non-stressed control group. Spatial memory was assessed by the Morris water maze test (120 cm diameter, filled with opaque white milk solution, and with extra-maze cues surrounding the pool) with hidden platform which was submerged 1.5 cm below the liquid surface.^22^ The observer was blinded to treatment, and mice of the two groups were tested in a random order. Behavioral testing was started after the stress period. Mice received 10 days of acquisition training with four trials per day. During the training period, the mice were each day randomly let into the pool from four directions. If mice found the platform within 60 s, they were allowed to stay there for 30 s. If mice did not find the platform, they were guided to the platform and stayed there for 30 s. After 10 days of training, the submerged platform was removed, and memory retention was assessed in a single probe trial of 60 s during which the time spent in the four different quadrants was measured.^22^

#### Generation of transgenic mice

For the study, the following double-transgenic mouse lines were generated: Tg2576-CMV*GRK2*, Tg2576-CMV*GRK2S670D*, Tg2576-CMV*GRK2K220R*, Tg2576-CMV*GRKInh*, Tg2576-CosSHa*TOMM6*, and Tg2576-CMV*ARRB1* mice. Double-transgenic mice were generated by crossbreeding of the respective female, homozygous, single-transgenic mice with male Tg2576 mice. The method of transgenic mouse generation was performed according to our standard protocols,^27^^,^^28^^,^^54^^,^^59^ which were adapted as follows. For generation of single-transgenic mice with neuron-specific expression of *TOMM6* under control of the Syrian hamster prion protein promoter (*Prp*), the cDNA encoding *TOMM6* was inserted into the Sal I site of the CosSHa.Tet Vector (InPro Biotechnology, Inc.). The linearized, purified Not I fragment (1–2 ng/μL in microinjection buffer, consisting of 8 mM Tris, 0.15 mM EDTA, pH 7.4, in ultrapure water) was microinjected into the pronucleus of fertilized oocytes of super-ovulated B6 (C57BL/6J) and FVB (FVB/N) mice. After overnight incubation, two-cell stage embryos were transferred into pseudo-pregnant CD-1 foster mice by oviduct transfer. Offspring founder mice with integration of the transgene into genomic DNA were identified by genotyping PCR of ear-punch biopsies and used for further breeding. For transgene expression under control of the CMV promoter, the cDNAs encoding *GRK2*,^27^ *GRK2S670D*,^26^ *GRK2K220R*,^27^ *GRKInh,*^27^^,^^28^ and *ARRB1*,^54^ were inserted into the pcDNA3.1 plasmid (Invitrogen). After removal of plasmid sequences by MluI and DraIII digestion, purified, linearized DNA (1–2 ng/μL) was microinjected into the pronucleus of fertilized oocytes of super-ovulated B6 (C57BL/6J) and FVB (FVB/N) mice as detailed above. Transgenic mice with FVB background were backcrossed for 10 generations into B6 background. Female homozygous offspring were used for crossbreeding with male Tg2576 mice having B6 background to generate double-transgenic mice. The stable integration of both transgenes into the genomic DNA was routinely controlled by genotyping PCR. The following oligonucleotides were used for genotyping PCR (Table S4): CosSHa-forward 5′-GCT TCA GCC TGC GTG CTG GAC AAT GAC GTG-3`; TOMM6-reverse 5′-CCT GGC TGA GGT GCC ATG AGG TCA ATG TCA C-3`; Prp-forward 5′-AAG CGG CCA AAG CCT GGA GGG TGG AAC A-3′; APP-forward 5′-CTG ACC ACT CGA CCA GGT TCT GGG T-3′; Prp-reverse 5′-GTG GAT AAC CCC TCC CCC AGC CTA GAC CA-3′; GRK2-forward 5′-GCC TGC CCA TGG AGG AGA TCC AGT CGG-3′; GRKInh-forward 5′-ATG GCC AAG TTC GAG CGC CTG CAG ACC GTG-3′; Sp6-reverse 5′-TAG AAG GCA CAG TCG AGG CTG ATC AGC GAG-3′; CMVforward 5′-CGC AAA TGG GCG GTA GGC GTG-3′; ARRB1-reverse 5′-GGT CAG GCC CAG GAC ATC CAG GTC CTC CCG-3′; TAU-forward 5′-GAC CAA GAG GGT GAC ACG GAC GC-3′; TAU-reverse 5′-TGC CGC CTC CCG GGA CGT GTT TG-3′.

Sperm of transgenic mouse lines generated in frame of the study were cryopreserved by mouse repositories and have the following numbers: FVB/N-Tg(CMV*GRK2*) 3 Sjaa, JAX strain ID 419257; FVB/N Tg-(CMV*GRK2S670D*) Sjaa, Janvier Labs ID 202.586 LASC PHARMAKOLOGIE; FVB/N-Tg(CosHa*TOMM6*) 4 Sjaa, JAX strain ID 419255; C57BL/6Tg(CMV*GRKInh*) 1 Sjaa, JAX strain ID 911820; C57BL/6Tg(CMV*GRKInh*) 4 Sjaa, JAX strain ID 419256; C57BL/6Tg(CMV*GRK2K220R*) 1 Sjaa, JAX strain ID 911824; C57BL/6-Tg(CMV*ARRB1*) 1 Sjaa, Janvier Labs ID 181.077, and 182.534 ETH Zurich.

The study compared groups of single-transgenic Tg2576 mice (age groups of 16-, and 18-month-old, female and male mice) with age-matched, double-transgenic Tg2576-CMV*GRK2*, Tg2576-CMV*GRK2S670D*, Tg2576-CMV*GRK2K220R*, Tg2576-CMV*GRKInh*, Tg2576-CMV*ARRB1*, and Tg2576-CosSHa*TOMM6* mice (female and male). In addition, protein studies were performed with brains isolated from 12-month-old and 18-month-old Tg2576 mice, Tg2576-CMV*GRK2* mice and Tg2576-CMV*GRK2K220R* mice (female and male), as indicated. As controls for protein studies, 6-month-old, 12-month-old and 18-month-old non-transgenic B6 mice (female and male) were included. The impact of the two major hallmark proteins of AD, Aβ and neurofibrillary-tangle-inducing, TAU-P301L, on serine-670-GRK2 phosphorylation was investigated with 12-month-old, male, double-transgenic Tg2576-Tg(Prnp-MAPT∗P301L)JNPL3Hlmc mice in comparison to 12-month-old, male, single-transgenic Tg2576 mice. After the indicated observation periods, anesthetized mice (ketamine/xylazine 100/10 mg/kg, i.p.) under temperature control to avoid hypothermia were intracardially perfused with PBS, and brains were rapidly isolated on ice.

For immunoblots and biochemical analyses, processing of brain specimens was performed as detailed below. After formalin fixation (10% neutral buffered formalin), histological tissue specimens were processed for paraffin embedding with a vacuum tissue processor (ASP200, Leica Biosystems). Paraffin-embedded sections (8 μm) were cut with a microtome (Leica RM 2235). For OCT-embedding, paraformaldehyde-fixed (4%, freshly prepared) specimens were infiltrated with 15% sucrose in PBS for 6 h followed by 30% sucrose in PBS overnight, mounted on a tissue holder with OCT (Tissue-Tek) and frozen with cryo spray. Sections (10 μm) were cut with a cryostat (Microm HM 525).

#### Microarray gene expression profiling

To confirm the diagnosis of probable AD in four patients with dementia and glioblastoma, whole genome microarray gene expression profiling was performed of cerebral cortex biopsy specimens obtained in frame of brain surgery for glioblastoma. The comparison group consisted of 34 cerebral cortex biopsy specimens from 34 glioblastoma patients without dementia. In total, cerebral cortex specimens from 38 glioblastoma patients (31 females, 7 males; age 59.15 ± 9.60 years [mean ± s.d.]) were included in the study. Specimens were processed for whole genome microarray gene expression profiling similarly as described.^59^ Patient characteristics are shown in Table S3. For RNA isolation, cerebral cortex biopsy specimens were immediately stabilized in RNAlater and stored at −80°C. Total RNA was isolated from cerebral cortex specimens with the RNeasy Mini kit according to the protocol of the manufacturer (Qiagen). For whole genome microarray gene expression profiling, the synthesis of complementary DNA, labeling, and hybridization of fragmented cRNA (15 μg) with the gene chip (GeneChip Human Genome U133 Plus 2.0 Array Affymetrix) were performed according to the Affymetrix protocol (Affymetrix GeneChip Expression Analysis Technical Manual Rev. 5). Selected, AD-related transcripts with up-regulation in brain specimens of the four patients with dementia likely due to AD are shown in Figure S9.

With an essentially similar workflow, whole genome microarray gene expression profiling was performed of frontal cortex specimens from 18-month-old, male Tg2576 mice without and with treatment for 6 months with CPD10 in drinking water (8 mg/kg/d). At the end of the observation period, frontal cortices were isolated from brains, conserved in RNAlater, and rapidly frozen in liquid nitrogen. The total RNA was isolated with the RNeasy Mini kit and processed for microarray gene expression profiling as detailed above. Fragmented cRNA (15 μg) was hybridized with the GeneChip (Affymetrix GeneChip Mouse Genome MG430 2.0 Array). For hybridization, fragmented cRNA was pooled from frontal cortices of three male mice per group (6 male mice for two GeneChips per group). Probe sets of genes with significant up-regulation in the CPD10-treated Tg2576 group compared to the untreated control group (with *p* < 0.05, as determined with the unpaired, two-tailed *t* test, just alpha, no correction, and ≥2-fold up-regulation, and probe set intensity ≥100) were selected (*n* = 326 genes; Table S5) and used for overrepresentation analysis with g:GOSt of g:Profiler (g:Profiler version e113_e.g.,59_p19_f6a03c19, database updated on 23/05/2025). Up-regulated genes were categorized by GO analysis selecting GO biological process (GO:BP; query size *n* = 309). CPD10 treatment led to up-regulation of genes with high overrepresentation in GO:BP terms related to neurogenesis with a significance threshold of *p* < 0.001 (cf. Figure 6J; Table S5).

Whole genome microarray gene expression data have been deposited in the NCBI GEO database: GSE317145 (https://www.ncbi.nlm.nih.gov/geo/query/acc.cgi?acc=GSE317145), GSE318045 ((https://www.ncbi.nlm.nih.gov/geo/query/acc.cgi?acc=GSE318045), GSE318046 (https://www.ncbi.nlm.nih.gov/geo/query/acc.cgi?acc=GSE318046), GSE318047 (https://www.ncbi.nlm.nih.gov/geo/query/acc.cgi?acc=GSE318047).

#### Serum and brain levels of CPD10 and CPD57

Serum concentrations of CPD10 and CPD57 were determined with serum (0.2 mL) taken by cardiac puncture from anesthetized 18-month-old Tg2576 mice at the end of the observation period, after oral dosing of CPD10 (2.7, 8, 24 mg/kg/d) and of CPD57 (3, 10, 30 mg/kg/d) for six months. Serum proteins were removed by acetonitrile precipitation (3:1). After centrifugation at 10,000 × g, CPD10 and CPD57 in the supernatant were extracted by addition of chloroform (1:1). After phase separation, the chloroform phase was evaporated to dryness, and the dried sample was dissolved in 20 μL of acetonitrile. The concentrated sample was analyzed (injector volume 10 μL) on an HPLC-C18 column (Poroshell 120 EC-C18, 3.0 × 50 mm, particle size 2.7 μm, Agilent), by using a linear solvent gradient (30%–95% of solvent B for CPD10; and 5%–95% of solvent B for CPD57; solvent A: 0.1% TFA in H_2_O; solvent B: acetonitrile) on an HPLC system (Agilent 1100 Series) and detection at OD 280 nm (CPD10) and at OD 254 nm or 245 nm (CPD57), in comparison to standards (analysis time 20 min; column temperature 40°C). Initially, the extraction and detection methods were validated by addition of fixed amounts of internal standard, to compound-free serum and brain samples. Brain contents of CPD10 and CPD57 were determined with whole brains (without cerebellum) of 18-month-old Tg2576 mice at the end of the treatment period. Brains were frozen in liquid nitrogen and homogenized in physiological salt solution. After removal of proteins by acetonitrile precipitation, and after chloroform extraction, brain contents of CPD10 and CPD57 were determined as detailed above.

#### Recombinant GRK2 and GRK2-S670A proteins

Recombinant GRK2 and GRK2-S670A were expressed in *Spodoptera frugiperda* (Sf9) cells by the baculovirus expression system. The cDNAs encoding hexahistidine-tagged His_6_-*GRK2* and His_6_-*GRK2S670A* were inserted into the pFastBac1 expression vector (Invitrogen; Thermo Fisher Scientific) using the SalI-HindIII restriction sites. Recombinant baculoviruses were generated by the Bac-To-Bac Baculovirus Expression System (Invitrogen; Thermo Fisher Scientific, Cat. No. 10359016) according to the protocol of the company (Version D, 2004, and MAN0000414, revision A.0, 2015). Forty-eight hours after viral transduction at a multiplicity of infection (MOI) of 2–3, recombinant baculovirus-infected Sf9 cells were harvested by centrifugation, washed with PBS, and frozen in liquid nitrogen. Frozen cell pellets were thawed and suspended in lysis buffer (300 mM NaCl, 50 mM HEPES, pH 7.5 supplemented with 1% NP40, 1 mM PMSF and protease inhibitor cocktail 1:100 [Cat. No. P8340, Sigma]). After sonication on ice and centrifugation at 4°C for 50 min (4500 × g), cytosolic proteins in the supernatant were collected. Protein samples were applied to the Ni-NTA agarose resin (Cat. No. 30210, Qiagen) pre-washed with 20 column volumes of lysis buffer. After overnight incubation at 4°C under gentle agitation, unbound proteins were removed by washing with lysis buffer (20 × column volumes), followed by a washing step with one column volume of 30 mM imidazole-containing lysis buffer. Bound proteins were eluted by 300 mM imidazole in lysis buffer and desalted by PD10 column chromatograpy. Proteins were freshly used or supplemented with 20% glycerol and stored at −80°C for further use.

#### Recombinant TOMM6 and *TOMM6-ST-AA* proteins

The cDNAs encoding human *His*_*6*_*-TOMM6*, and *His*_*6*_*-TOMM6-ST-AA* (TOMM6 mutant with S3-S4-T5-S9-S13-T17 exchanged to alanine), and *ERK1* were subcloned into the PET-3d expression vector (Novagen) and used for expression in BL21(DE3)pLysS bacteria (Novagen). Bacteria were collected by centrifugation, the bacterial pellet was frozen in liquid nitrogen and thawed on ice in lysis buffer (8 M urea, 300 mM NaCl, 50 mM HEPES, 10 mM imidazole, 10 mM 2-mercaptoethanol, pH 7.5). Thereafter the bacterial lysates were homogenized by sonication on ice, and centrifuged for 15 min at 4000 × g at 4°C. The supernatant was applied to the Ni-NTA resin prewashed with 20 mL of lysis buffer. After overnight incubation at 4°C under gentle agitation, the flow-through was discarded, and the Ni-NTA affinity matrix was subjected to 3 different washing steps. Each washing step included 30 min of incubation at 4°C with 20 mL of each washing buffer per mL of Ni-NTA affinity resin. The three different washing buffers consisted of 300 mM NaCl, 50 mM HEPES, 20 mM imidazole, pH 7.5, supplemented freshly with 10 mM 2-mercaptoethanol, but contained different concentrations of urea (4 M, 2M and 0 M urea). After the different washing steps, proteins were eluted with elution buffer (300 mM NaCl, 50 mM HEPES, 500 mM imidazole, pH 7.5, supplemented freshly with 10 mM 2-mercaptoethanol). Buffer exchange was performed by PD-10 column chromatography, and protein concentration was performed by centrifugation through Amicon Ultra Centrifugal Filter units with a molecular weight cut-off of 3 kDa (Merck). Proteins were freshly used, or supplemented with 20% glycerol for storage at −80°C.

#### *In vitro* phosphorylation assay by GRK2

The *in vitro* phosphorylation assay by GRK2 was performed similarly as described,^26^ with the following modifications. The *in vitro* phosphorylation assay used recombinant, serine-670-phosphorylated GRK2 (phospho-GRK2), GRK2, and GRK2-S670A. The serine-670 phosphorylation of GRK2 was induced as described,^26^ by incubation of purified, recombinant GRK2 (2 μM) with purified, pre-activated ERK1 (30 nM) for 45 min at 30°C in incubation buffer (40 mM HEPES, pH 7.5, 5 mM MgCl_2_, 2 mM dithiothreitol) supplemented with ATP (60 μM) and heparin (molecular weight 4000–6000 Da; 7 μg/mL). The reaction was stopped by removal of heparin and ATP by Zeba Spin desalting columns (7K MWCO; Thermo Fisher Scientific). After the addition of 150 mM NaCl, samples were cleared from ERK1 by binding to anti-ERK1 antibodies coupled to Affigel-10 matrix. The phospho-GRK2-containing flow-through was collected and subjected to buffer exchange with Zeba Spin desalting columns. Phospho-S670-GRK2 contents and the absence of ERK1 were assessed by immunoblot analysis. Freshly prepared phospho-S670-GRK2, GRK2 and GRK2-S670A (100 nM) were each used for substrate phosphorylation, which was performed in reaction buffer (50 μL of 20 mM Tris, 2 mM EDTA, 10 mM MgCl_2_, 1 mM dithiothreitol pH 7.5) supplemented with 60 μM ATP, and radiolabeled [γ-^32^P]-ATP (1 × 10^6^ DPM, specific activity 3000 Ci/mmol) with 300–500 nM of substrate (TOMM6, TOMM6-ST-AA with S3-S4-T5-S9-S13-T17 exchanged to alanine) without and with increasing concentrations of CPD10, as indicated. After an incubation for 30 min at 30°C, each reaction was stopped by the addition of 5 × SDS-Laemmli buffer. Proteins were separated by SDS-PAGE and subjected to autoradiography.

If the phosphorylation assay was performed as a filter assay, the phosphorylation reaction was stopped by the addition of 5 volumes of ice-cold PBS washing buffer. The reaction mixtures were immediately applied to glass fiber filters (GF/C, Whatman). After three washing steps with 5 mL of washing buffer, filter-bound radioactivity was quantified in a β-counter (Beckman Coulter LS6500). As a control, background phosphorylation (<3%) was determined in the presence of the GRK2 inhibitor heparin (7 μg/mL).

#### Biochemical kinase profiling

*In vitro* phosphorylation assays in the absence and presence of CPD10 (10 μM) were performed by Thermo Fisher Scientific’s SelectScreen Profiling Service with the SSBK-Z′-LYTE assay for profiling of kinases GRK5, PRKACA (PKA), CAMK2D, PRKCA (PKC alpha), MAPK3 (ERK1), SRPK1, AKT1, GSK3B, and with the SSBK-ADAPTA assay for DAPK1. Kinase activities of GRK3 and GRK6 were assessed with recombinant purified kinases (Eurofins DiscoverX, Catalog no. 15–028, 14–715) and tubulin as a kinase substrate.

#### Target profiling of CPD10

Target profiling by radioligand competition assay in the absence and presence of CPD10 (10 μM) was performed by EUROFINS Discovery for the following target proteins: ADORA2A, ADRA1A, ADRA2A, ADRB1, ADRB2, BZD ([^3^H]flunitrazepam binding site, rat cerebral cortex), CNR2, CNR1, CCKAR (CCK1), DRD1, DRD2 (short isoform), EDNRA, NMDA receptor (rat cerebral cortex), HRH1, HRH2, MAOA ([^3^H]Ro 41–1049 binding site, rat cerebral cortex), CHRM1, CHRM2, CHRM3, CHRNA4, OPRD1, OPRK1, OPRM1, HTR1A, HTR1B, HTR2A, HTR2B, HTR3A, NR3C1 (GR), AR (human endogenous of LNCaP cells), AVPR1A, Ca2+ channel (L-dihydropyridine site, rat cerebral cortex), KCNH2 (Potassium channel hERG; [^3^H]Dofetilide binding site), Kv-channel ([^125^I]α-dendrotoxin binding site, rat cerebral cortex), NET, DAT1, and SERT. For radioligand competition assays, the human recombinant protein expressed in HEK293 cells or CHO cells was applied if not indicated otherwise. The inhibition of the enzymatic activity by 10 μM of CPD10 was tested for human recombinant enzymes LCK, COX1, COX2, PDE3A, PDE4D2 and ACHE (acetylcholinesterase). Data are presented in Table S1, and show no off-target effects of CPD10, i.e., no inhibition of any of the 43 tested pharmacological and toxicological targets by more than 40% with 10 μM of CPD10.

#### MDS of recombinant TOMM6

Microfluidic diffusional sizing (MDS) used a Fluidity One-W Serum machine (Fluidic Analytics, Cambridge, UK) with Fluidity One-W disposable chips (Item Number: 100009; Fluidic Analytics Ltd., Cambridge, UK) to determine the hydrodynamic radius of recombinant TOMM6 in solution. For MDS, TOMM6 was labeled with Alexa Fluor 647 NHS Ester (Invitrogen), by incubation of 0.3 mg of purified recombinant His_6_-TOMM6 in 500 μL of 0.1 M sodium bicarbonate solution, pH 8.3, with 100 μg of Alexa Fluor 647 NHS Ester for 1 h, at RT, under continuous stirring. After removal of free Alexa Fluor 647 by centrifugation over Zeba spin desalting columns (7K MWCO) and buffer exchange, MDS was performed with different concentrations of Alexa Fluor 647-labeled TOMM6 (10 nM - 1 μM) in 10 mM HEPES buffer, pH 7.4, at 24°C. MDS determined the hydrodynamic radius (Rh) of TOMM6, which is the Stokes radius. Conversion into the unfolded molecular weight of TOMM6 was performed by the hydrodynamic radius converter (Fluidic Sciences; https://fluidic.com/molecular-weight-to-hydrodynamic-radius-converter/; accessed on January 31, 2026).

#### Phospho-S670-GRK2-interacting proteins

The method of immunoaffinity enrichment of phospho-S670-GRK2 from brain mitochondrial proteins of 18-month-old and 12-month-old, female and male Tg2576 mice, and from human cerebral cortex mitochondrial proteins, and the method for identification of co-enriched, phospho-S670-GRK2-interacting proteins, was adapted from a previously described protocol.^59^ For a typical immunoaffinity enrichment (defined as one biological replicate), frontal cortices and hippocampi from at least five female or five male mouse brains were dissected on ice, and pooled. The mitochondrial proteins were isolated as detailed below, and solubilized in solubilization buffer (1% sodium deoxycholate, 0.05% SDS, 0.05% Tween 20 in PBS, pH 7.4 supplemented with protease inhibitor cocktail, P8340 [Sigma], and phosphatase inhibitors) for 30 min at 4°C. After centrifugation to remove insoluble material, the supernatant was diluted 1:5 in PBS (supplemented with protease inhibitors and phosphatase inhibitors) and applied to the affinity matrix with covalently coupled polyclonal anti-phospho-S670-GRK2 antibodies (6 mg of affinity-purified IgG coupled to 1 mL of Affigel-10; Bio-Rad). Polyclonal anti-phospho-S670-GRK2 antibodies were raised in rabbit against a KLH-conjugated phospho-peptide encompassing the S670-phosphorylated carboxyl-terminal region of human GRK2 (Cat. No. PA5-77851, Invitrogen, and this work).

After overnight incubation of the mitochondrial lysates with the anti-phospho-S670-GRK2 antibody-coupled affinity matrix under gentle agitation at 4°C, unbound proteins were removed by washing with PBS (10 column volumes). Bound proteins were eluted with 0.25 M NH_4_OH, 10% dioxane (pH 11), and the pH of the eluate was immediately adjusted to pH 7.4. Thereafter, eluted proteins were concentrated by acetone precipitation, dissolved in 2 × Laemmli sample buffer, and subjected to urea-containing SDS-PAGE under reducing conditions. Enriched phospho-S670-GRK2, and co-enriched proteins were identified by immunoblot detection. As indicated, phospho-S670-GRK2-interacting proteins were also identified by nano-LC-ESI-MS/MS analyses (as detailed below).

#### Co-enrichment of TOMM40 with TOMM6

Aggregated TOMM6 was immunoaffinity-enriched from brain (hippocampus and frontal cortex) mitochondrial lysates of 18-month-old, female and male Tg2576 mice with an anti-TOMM6 antibody-containing affinity matrix (6 mg of affinity-purified IgG coupled to 1 mL of Affigel-10; Bio-Rad). The controls were 18-month-old, female and male, non-transgenic B6 mice with monomeric TOMM6. The method of immunoaffinity enrichment of TOMM6 was essentially performed as described above, with mitochondrial proteins isolated from frontal cortices and hippocampi pooled from three female or three male mice for one immunoaffinity enrichment (defined as one biological replicate). Solubilized proteins were incubated overnight at 4°C under gentle agitation with the anti-TOMM6-antibody-coupled immunoaffinity matrix. After removal of unbound proteins by washing steps with PBS, bound proteins were eluted, concentrated, and subjected to urea-containing SDS-PAGE under reducing conditions, as detailed above. Immunoaffinity-enriched TOMM6 was identified by immunoblot with anti-TOMM6 antibodies (Cat. No. HPA004801; Sigma Prestige Antibodies), and co-enriched TOMM40 was identified by immunoblot detection with anti-TOMM40 antibodies (Cat. No. 18409-1-AP; Proteintech).

#### Detection of phosphorylated TOMM6

Aggregated and monomeric TOMM6 proteins were enriched by immunoaffinity chromatography from mitochondrial brain proteins (frontal cortex, hippocampus) of 18-month-old, female and male Tg2576 mice without and with 6 months of treatment with CPD10 (8 mg/kg/d) with an anti-TOMM6 antibody-containing affinity matrix (6 mg of affinity-purified IgG coupled to 1 mL of Affigel-10; Bio-Rad), as detailed above. Brains (frontal cortices and hippocampi) from three female or three male mice were pooled for one protein isolation, which is defined as a biological replicate. Eluted TOMM6 was concentrated and subjected to urea-containing SDS-PAGE under reducing conditions, followed by immunoblot detection of immunoaffinity-enriched TOMM6 with anti-TOMM6 antibodies (Cat. No. HPA004801, Sigma Prestige Antibodies). The phosphorylation status of immunoaffinity-enriched TOMM6 was assessed by immunoblot with anti-phosphoserine-specific antibody (clone 4A4; Cat. No. 05–1000; EMD Millipore Corp.).

#### Co-enrichment of ARRB1 with APH1A

APH1A was immunoaffinity enriched from the microsomal membrane fractions prepared from brains (frontal cortex and hippocampus) of 18-month-old Tg2576 mice and 18-month-old Tg2576-*ARRB1* mice. Frontal cortices and hippocampi from three female or three male mice were pooled for one isolation (defined as one biological replicate). Membranes were solubilized for 30 min in solubilization buffer (10 mM PBS, pH 7.4, containing 1% sodium deoxycholate, and supplemented with protease inhibitors and phosphatase inhibitors). After a centrifugation step, the supernatant was diluted 1:5 with PBS (supplemented with protease inhibitors and phosphatase inhibitors) and incubated overnight at 4°C with the affinity matrix (Affigel-10; Bio-Rad) with covalently coupled rabbit polyclonal anti-APH1A antibodies (Cat. No. 11643-1; Proteintech). Bound proteins were eluted after washing steps, concentrated, and subjected to SDS-PAGE supplemented with urea under reducing conditions (as detailed above). Immunoaffinity-enriched APH1A(-L) was detected in immunoblot with APH1A antibodies (1:2000; rabbit polyclonal anti-APH1A antibodies, HRP-conjugated, raised against the C-terminus of human APH1A; Cat. No. PB9421; Boster Biological Technology), and co-enriched ARRB1 was detected in immunoblot with anti-ARRB1 antibody (dilution 1:1000; mouse monoclonal anti-ARRB1 antibody (Clone 1H8E4; Cat. No. 67580-1-Ig; Proteintech).

#### Ligand affinity chromatography

The ligand affinity chromatography matrix was generated by coupling of NH2-CPD10 [2-(4-aminophenyl)-1-(1,3-benzodioxol-5-yl)-4-(cyclopropanecarbonyl)-3-hydroxy-2H-pyrrol-5-one], and CPD57 to Affigel-10 (5 mg/mL; Bio-Rad), and to Epoxy-activated Sepharose 6B (5 mg/mL; Cytiva), according to the protocol of the manufacturers. Frontal cortices and hippocampal specimens from three female or three male mice (transgenic mice and non-transgenic B6 mice, as indicated) were pooled for one ligand affinity chromatography experiment (defined as one biological replicate). Proteins were pulverized under liquid nitrogen and solubilized for 30 min at 4°C in solubilization buffer (10 mM PBS, pH 7.4, containing 1% sodium deoxycholate, and supplemented with protease inhibitors and phosphatase inhibitors). After centrifugation to remove insoluble material (60 min, 100,000 × g, 4°C), the supernatant was collected, diluted 1:3 - 1:5 with PBS (with protease inhibitors and phosphatase inhibitors) and applied to the ligand affinity matrix. After overnight incubation at 4°C under gentle agitation, unbound proteins were removed by washing with 20 column volumes of PBS. Elution of bound proteins was performed with elution buffer (0.25 M glycine, pH 2.5; or 0.25 M NH_4_OH, 10% dioxane, pH 11), followed by immediate neutralization of the eluted protein fractions to pH 7.4. For concentration of eluted proteins, centrifugation through Amicon Ultra centrifugal filters (Amicon Ultra Centrifugal Filter, molecular weight cut off 3 kDa, Merck Millipore, Cat. No. UFC8003) was performed. Thereafter, proteins were dissolved in 2 × Laemmli sample buffer and subjected to SDS-PAGE under reducing conditions. Enriched proteins were identified by immunoblot detection and by nano-LC-ESI-MS/MS analyses (as detailed below).

#### Nano-HPLC-ESI-MS/MS analyses

After enrichment of mitochondrial phospho-S670-GRK2 from hippocampal and frontal cortex specimens of 12-month-old Tg2576 mice, co-enriched phospho-S670-GRK2-interacting proteins were identified by nano-LC-ESI-MS/MS analyses. To this end, after immuno-affinity enrichment, isolated proteins were concentrated, dissolved in 2 × Laemmli sample buffer, and subjected to SDS-PAGE under reducing conditions. Enriched protein bands were visualized by Coomassie Brilliant Blue staining of the SDS-containing gel, cut out from the gel, and subjected to nano-HPLC-ESI-MS/MS analysis. The phospho-S670-GRK2-interacting TOMM6 protein was identified in the gel slice encompassing the 6–12 kDa protein range.

CPD57 ligand affinity chromatography was performed as detailed above. Proteins, which were eluted from the ligand affinity matrix, were concentrated, dissolved in 2 × Laemmli sample buffer and separated by SDS-containing PAGE under reducing conditions. CPD57-enriched proteins in the SDS-containing gel were stained with Coomassie Brilliant Blue, protein bands were cut out and subjected to nano-HPLC-ESI-MS/MS analysis. The CPD57-interacting PSMA3 and the other 20S proteasome alpha subunits were identified in the gel slice encompassing the 25–30 kDa protein range.

The nano-HPLC-ESI-MS/MS analysis was performed by Proteome Factory AG (Berlin, Germany), as described.^59^ Proteins were identified with an LC-MS system consisting of an Agilent 1100 nano-HPLC system (Agilent, Waldbronn, Germany), a PicoTip electrospray emitter (New Objective, Woburn, MA) and an Orbitrap Velos mass spectrometer (Thermo Fisher Scientific, Bremen, Germany). First, peptides were trapped and desalted on the enrichment column (Zorbax 300SB-C18, 0.3 × 5 mm, Agilent; solvent: 2.5% acetonitrile/0.5% formic acid). Thereafter peptides were separated on a Zorbax 300SB-C18 column (Agilent; 0.075 × 150 mm column; linear gradient from 15% to 40% solvent B; solvent A: 0.1% formic acid in water; solvent B: 0.1% formic acid in acetonitrile). Ions of interest were data-dependently subjected to MS/MS according to the expected charge state distribution of peptide ions. Proteins were identified by database searches against sequences from *M. musculus* (UP589_M-musculus 202501126: 63,149 sequences, 28,420,943 residues) from the Uniprot database and a database with known protein contaminants (contaminants 20160129; 247 sequences; 128,130 residues) using MS/MS Ions Search of the Mascot search engine, Mascot version 3.0.0 (timestamp 2025-02-01T08:45:16Z for TOMM6; timestamp 2025-02-01T08:03:51Z for PSMA3).

The following settings for the Mascot MS/MS Ions Search were used: The number of missed and/or non-specific cleavages permitted was adjusted to 1. The mass tolerance for precursor ions was set to ±3 ppm, and the mass tolerance for fragment ions was ±0.6 Da. Trypsin was used as the protease to generate peptides. The settings of the Mascot search were adjusted to identify carbamidomethyl (C) as a fixed modification, and deamidation (NQ) and oxidation (M) as variable modifications. Results were refined by machine learning with features calculated by Mascot. Protein matches had at least two significant unique sequences and a score of 200 or above.

Mass spectrometry proteomics data of TOMM6 and PSMA3 identification by nano-LC-ESI-MS/MS analysis have been deposited to the ProteomeXchange Consortium via the PRIDE,^60^ partner repository with the dataset identifiers: PXD073643 (https://doi.org/10.6019/PXD073643) and PXD073574 (https://doi.org/10.6019/PXD073574).

#### Isolation of synaptosomal proteins

For isolation of synaptosomal proteins, hippocampal specimens of female and male mice of the indicated mouse lines were homogenized on ice in Syn-PER Reagent (10 mL reagent per gram of tissue; Cat. No. 87793; Thermo Fisher), supplemented with freshly added protease and phosphatase inhibitor cocktail with a Dounce tissue grinder. The homogenate was centrifuged at 1200 × g for 10 min at 4°C. The resulting supernatant was collected, and centrifuged at 15,000 × g for 20 min at 4°C. The crude synaptosomal pellet was collected. Synaptosomal proteins were solubilized in SDS-sample buffer containing 2% SDS, 0.1 M DTT (dithiothreitol), supplemented with protease and phosphatase inhibitors and 6 M urea for 90 min at room temperature. After the addition of iodoacetamide (10 mM), samples were stored at −70°C for further use.

#### Mitochondria isolation

The mitochondria isolation method was adapted from a previously published protocol.^61^ For mitochondria isolation, mouse hippocampi (six freshly isolated hippocampi from three female or three male mice of the indicated mouse lines were pooled for one biological replicate) or mouse brains (freshly isolated frontal cortices and hippocampi from three female or three male mice were pooled for one biological replicate) and human cortex specimens (0.3 g–0.5 g, fresh biopsy specimens obtained during surgery for glioblastoma) were suspended in ice-cold isolation buffer (5 mM HEPES, pH 7.2, 225 mM mannitol, 75 mM sucrose, 2 mM K_2_PO_4_, pH 7.4, supplemented with protease inhibitors and phosphatase inhibitors). After mincing, the isolation buffer was decanted and replaced by fresh isolation buffer (brain mass to buffer volume ratio 1:10). The brain tissue was homogenized with a Potter-Elvehjem homogenizer and diluted 1:1 with isolation buffer. The homogenate was centrifuged at 1500 × g for 5 min at 4°C. The supernatant was collected and centrifuged for 10 min at 10,300 × g at 4°C to yield the crude mitochondrial pellet (with contaminating synaptosomal membranes, myelin, ER, and MAMs), and the cytosolic proteins in the supernatant. The supernatant was centrifuged for 60 min at 95,000 × g to remove ER proteins, and the resulting cytosolic protein fraction in the supernatant was collected. The cytosolic protein fraction was then concentrated by centrifugation through Amicon Ultra centrifugal filters (Amicon Ultra-4 Centrifugal Filter Unit, molecular weight cutoff 3 kDa). Concentrated cytosolic proteins were solubilized as described below. The crude mitochondrial pellet from above was suspended in 1 mL of isolation buffer and layered above 10 mL of 30% Percoll in isolation buffer (vol/vol; GE Healthcare). After ultracentrifugation at 95,000 × g for 60 min at 4°C, the mitochondria-containing band with the highest density was collected and diluted with isolation buffer (1:10). Mitochondria were pelleted by centrifugation for 10 min at 8,000 × g at 4°C, collected, resuspended and washed again with 10-fold volume of isolation buffer and finally pelleted by centrifugation. The supernatant was discarded, and the mitochondrial pellet was solubilized in SDS-sample buffer containing 2% SDS, 0.1 M DTT, supplemented with protease inhibitors and phosphatase inhibitors and 6 M urea for 90 min at room temperature. Cytosolic proteins were processed likewise. After the addition of iodoacetamide (10 mM), samples were stored at a final protein concentration of 1 μg/μL at −80°C for further use.

#### Isolation of total brain proteins

For immunoblot detection of total hippocampal and frontal cortex proteins, frozen hippocampal and frontal cortex specimens (from female and male mice of the indicated mouse lines) were pulverized under liquid nitrogen and extracted with RIPA buffer (150 mM NaCl, 1% NP40, 0.5% sodium deoxycholate, 0.1% SDS, 25 mM Tris, pH 7.4) supplemented with protease inhibitor and phosphatase inhibitor cocktail. Particulate material was removed by centrifugation (20,000 × g for 15 min at 4°C). Proteins were precipitated and delipidated by acetone/methanol (12:2; final concentration 83%) for 90 min at 4°C. The precipitate was collected by centrifugation (5000 × g, 10 min, 4°C), which was followed by three washing steps with 0.2 mL of cold acetone. The pellet was dissolved in SDS-sample buffer containing 2% SDS, 0.1 M DTT, supplemented with protease inhibitors and phosphatase inhibitors and 6 M urea for 90 min at room temperature. After the addition of iodoacetamide (10 mM), samples were stored at −80°C for further use.

#### Immunoblot detection of proteins

After protein separation by SDS-containing PAGE under reducing conditions supplemented with 8 M urea, and protein transfer to PVDF membranes (Immobilon-P membrane, Merck Millipore), proteins were detected by immunoblot. For immunoblot detection, proteins were separated by urea-containing (8 M) SDS-PAGE (10% for detection of proteins <50 kDa; 7.5% for proteins >50 kDa) followed by electrophoretic protein transfer to PVDF membranes by semidry blotting or by wet blotting. Immunoblot detection of mitochondrial Aβ was performed after protein separation of mitochondrial proteins by urea-containing 15% SDS-PAGE. For urea-containing SDS-PAGE, the 1 × electrode buffer (14.4 g glycine and 3.0 g Tris base per 1000 mL) was supplemented with 0.2% SDS. Protein detection was performed with affinity-purified antibodies or F(ab)_2_ fragments of the respective antibodies, which had been pre-absorbed to mouse and human serum proteins. After blocking of the PVDF membrane with PBS supplemented with 0.05% (v/v) Tween 20 and 5% bovine serum albumin or non-fat dried milk for 1 h, primary antibodies were applied at a dilution of 1:2000 - 1:5000 in blocking buffer for 1 h. Unbound antibodies were removed by three washing steps with PBS supplemented with 0.05% (v/v) Tween 20, and the bound antibodies were visualized with F(ab)_2_ fragments of enzyme-coupled secondary antibodies (Jackson ImmunoResearch Laboratories; dilution 1:40,000), by peroxidase-coupled protein A (Calbiochem, Merck Millipore; dilution 1:10,000), or by peroxidase-coupled protein G (EMD Millipore Corporation; dilution 1:10,000), as applicable, followed by enhanced chemiluminescent detection (ECL Prime, GE Healthcare Cytiva; ECL Plus, Thermo Fisher Scientific).

#### Antibodies

The following affinity-purified antibodies were used for detection of proteins by immunoblot, for immunohistology, and for immuno-affinity enrichment of proteins: monoclonal anti-GRK2 antibody C-9 was raised in mouse against amino acids 468–689 of human GRK2 (sc-13143; Santa Cruz Biotechnology Inc., USA; Lot E0917; Lot G1410, Lot I1623, Lot L1119); polyclonal rabbit anti-human GRK2 antibody was raised in rabbit against a KLH-conjugated synthetic peptide encompassing a sequence within the N-terminus of human GRK2 (LS-C358899, LS Bio; Lot ID WOVU S31186); polyclonal anti-GRK2 antibodies were raised in rabbit against recombinant GRK2 expressed in baculovirus-infected Sf9 cells^27^^,^^28^; rabbit polyclonal anti-phospho-GRK2 (serine-670) antibodies were raised against a carrier-protein-conjugated peptide encompassing the S670-phosphorylated carboxyl-terminal region of human GRK2 (PA5-77851; Invitrogen; Lot ZH4432180D; ZI448570A; and this work); mouse monoclonal anti-Gαo (GNAO) antibody was raised against bovine Gαo (Clone A2; sc-13532; Santa Cruz Biotechnology; Lot A1005); rabbit monoclonal anti-ARRB1 antibody was raised against a synthetic peptide near the carboxyl terminus of the human ARRB1 protein (Clone D803J; Cat. No. 12697S; Cell Signaling Technology; Lot 3); mouse monoclonal anti-ARRB1 antibody was raised against recombinant human ARRB1 protein (Clone 1H8E4; Proteintech; Lot 10015333); rabbit polyclonal anti-APH1a antibodies were raised against a synthetic peptide corresponding to a sequence at the C-terminus of human APH1A (anti-APH1a antibody Picoband, HRP-conjugated, PB9421; Boster Biological Technology; Lot 25XD703K03); rabbit polyclonal anti-APH1A antibodies were raised against recombinant human APH1A protein aa 1–265 (Cat. No. 11643-1-PBS; Lot 00002255; Proteintech); rabbit polyclonal anti-TOMM6 antibodies were raised against the PrEST antigen TOMM6, which is recombinant TOMM6 with N-terminal His_6_ABP (albumin binding protein derived from streptococcal protein G) fusion tag (HPA004801; Sigma Prestige Antibodies, powered by Atlas Antibodies; Lot 000044616; Lot R07460; Lot A105468); polyclonal anti-TOMM6 antibodies were raised in rabbit against recombinant His_6_-TOMM6 (this work); rabbit polyclonal anti-TOMM40 antibodies were raised against human TOMM40-His_6_-fusion protein (18409-1-AP; Proteintech; Lot 00105350; Lot 00040008); rabbit polyclonal anti-ADSL antibodies were raised against recombinant human ADSL protein (PA5-51401; Invitrogen; Lot A118656); rabbit polyclonal anti-ADSL antibodies were raised against a recombinant fusion protein encompassing amino acids 1–310 of human ADSL (PA5-92861; Invitrogen; Lot AC46454371); mouse monoclonal anti-beta-amyloid antibody was raised against synthetic beta-amyloid peptide (clone BAM-10; A5213; ascites fluid; Sigma-Aldrich; Lot 103M4762; Lot 037K4877; Lot 087M4874V; Source 0000127507 with Batch #0000132482); mouse monoclonal anti-human PHF-tau (Ser202, Thr205) antibody was raised against partially purified human PHF-tau, recognizing a phosphatase-sensitive epitope on PHF-tau containing phosphorylated Ser202 residue (numbering according to human TAU40) without having cross-reactivity with normal tau (clone AT8, MN1020; Pierce Biotechnology Inc., Rockford, IL, USA; Lot HG105303; Lot IB112040; TL26525315); rabbit recombinant monoclonal anti-TAU antibody ([EP2456Y] (ab76128; Abcam; Lot GR3402049-7); mouse anti-Neuronal Nuclei (NeuN) monoclonal antibody (MAB377, Clone A60) was raised against purified cell nuclei from mouse brain (MAB377; Upstate/Chemicon; Lot LV1388597); rabbit monoclonal antibody against SNAP25 [EP3274] (ab108990; Abcam; lot 1018041-12); mouse monoclonal antibody against GSK3B was raised against amino acids 341–420 of human GSK3B (clone 1F7) (sc-53931; Santa Cruz Biotechnology; Lot B0117; Lot D1718); mouse monoclonal phospho-GSK3B (Ser9) antibody (Clone F-2) (sc-373800; Santa Cruz Biotechnology; Lot G2219; Lot S2217); mouse monoclonal anti-calcineurin (α-subunit) antibody (Clone CN-A1; C1956; Invitrogen; Lot 094M4820V); rabbit polyclonal anti-PPP2CA antibodies were raised against PPP2CA fusion protein Ag4294, which encompasses amino acids 1–309 of human PPP2CA (Cat. No. 13482-1-AP; Proteintech; Lot 00023021); rabbit recombinant monoclonal anti-PSMA3 antibody [EPR5455] (ab109532; Abcam; Lot 1003633-7); mouse monoclonal anti-UPAR antibody was raised against amino acids 1–290 of human UPAR protein (Clone E−3; Cat. No. sc-376494; Santa Cruz Biotechnology; Lot IO817); mouse monoclonal anti-Phosphoserine antibody (Clone 4A4; Cat. No. 05–1000; EMD Millipore Corp.; Lot 3754811; Lot 4290029).

Secondary antibodies: Peroxidase-conjugated AffiniPure F(ab’)_2_ Fragment Goat Anti-Mouse IgG, Fcgamma Fragment Specific (minimal cross-reaction to Human, Bovine, and Horse Serum Proteins) (115-036-071; Jackson ImmunoResearch Laboratories; Lot 77832; Lot 118230; Lot 151768); Peroxidase-conjugated AffiniPure F(ab’)_2_ Fragment Goat Anti-Rabbit IgG, Fcgamma Fragment Specific (minimal cross-rection to Human Serum Proteins) (111-036-046; Jackson ImmunoResearch Laboratories; Lot 84989; Lot 113992); Protein A, Peroxidase Conjugate, Native Protein A from *Staphylococcus aureus* conjugated to horseradish peroxidase (Cat. No. 539253; Calbiochem; Lot 3815982); Protein G, HRP conjugate (Catalog #18–161; Upstate/CHEMICON/Linco, EMD Millipore Corporation; Lot # 3734880); Alexa Fluor 488 goat anti-Mouse IgG (H + L) Highly Cross-Adsorbed Secondary Antibody (A-11029; Invitrogen Molecular Probes; Lot 57465A); Alexa Fluor 568 goat anti-Rabbit IgG (H + L) (A-11011; Invitrogen by Thermo Fisher Scientific; Lot 1811756).

#### Immunofluorescence of TOMM40 and GRK2

Immunofluorescence co-localization of mitochondrial TOMM40 with GRK2 was performed on cryosections of frozen brains from different study groups of 18-month-old, female and male mice (B6 mice, Tg2576 mice, and Tg2576-*TOMM6* mice, Tg2576 mice treated with CPD10). For preparation of cryosections, paraformaldehyde-fixed (4%, freshly prepared) specimens were placed in 15% sucrose in PBS for 6 h followed by incubation in 30% sucrose in PBS overnight. Thereafter specimens were mounted on a tissue holder with OCT (Tissue-Tek), and frozen with cryo spray. Specimens were cut on a cryostat (Microm HM 525) at 10 μm thickness at −20°C. Co-localization of TOMM40 and GRK2 on hippocampal specimens was performed with affinity-purified, rabbit polyclonal anti-TOMM40 antibodies (18409-1-AP; Proteintech) and mouse monoclonal anti-GRK2 antibody (sc-13143; Santa Cruz Biotechnology Inc.). Primary antibodies in blocking buffer (PBS with 5% [w/v] bovine serum albumin fraction V, 0.05% Tween 20, pH 7.4) were applied at a dilution of 1:400 for 1 h. After three washing steps with PBS supplemented with 0.05% Tween 20 to remove unbound antibodies, secondary antibodies labeled with Alexa Fluor 488 (Alexa Fluor 488 goat anti-Mouse IgG [H + L] Highly Cross-Adsorbed Secondary Antibody; A-11029; Invitrogen Molecular Probes) and labeled with Alexa Fluor 568 (Alexa Fluor 568 goat anti-Rabbit IgG (H + L); A-11011; Invitrogen by Thermo Fisher Scientific) were applied in blocking buffer at a dilution of 1:2000. After an incubation for 1 h, and three additional washing steps, cryosections were embedded in Aqua-Poly/Mount (Polysciences Inc.). Nuclei were stained with DAPI (Cat. No. 62248; Thermo Fisher Scientific). Imaging was performed with a confocal laser scanning microscope, CLSM (Leica TCS SPE and Leica SP8 Falcon; Center for Microscopy and Image Analysis at the University of Zurich, UZH). Analyses of colocalization of GRK2 with TOMM40 and of hippocampal mitochondrial phenotypes (area covered by clustered mitochondria) were performed with Fiji/ImageJ (ImageJ 1.54p) in a blinded manner with hippocampal specimens from 3 to 4 different mice per study group. All replicates of immunofluorescence co-localization studies of the mitochondrial protein TOMM40 with GRK2 on hippocampal specimens in different mouse groups are shown in Figures S1 and S7.

#### Immunofluorescence of GRK2 or TOMM40 and PHF

For immunofluorescence co-localization of GRK2 or TOMM40 with PHF-tau, cryosections were prepared from brains which were isolated from 16-month-old, male Tg2576 mice with 2 months of CUMS and without or with treatment with CPD10 (8 mg/kg/d) for two months. In addition, cryosections were prepared from brains of 12-month-old, male double-transgenic Tg(APPSWE)2576Kha-Tg(Prnp-MAPT∗P301L)JNPL3Hlmc mice (TgAPP-TAU) mice, as detailed above. The control group for TgAPP-TAU mice were 12-month-old, male single-transgenic Tg2576 (TgAPP) mice. For co-localization, rabbit polyclonal anti-GRK2 antibodies (raised against full-length recombinant GRK2 protein,^27^^,^^28^ or anti-TOMM40 antibodies (18409-1-AP; Proteintech), and mouse monoclonal anti-human PHF-Tau (Ser202, Thr205) antibody (clone AT8, MN1020, Invitrogen) were applied in blocking buffer (see above), at a dilution of 1:200 and 1:600, respectively. After an incubation for 1 h at 37°C and washing steps, the secondary antibodies were applied in blocking buffer at a dilution of 1:2000 (Alexa Fluor 488 goat anti-Mouse IgG [H + L] Highly Cross-Adsorbed Secondary Antibody; A-11029; Invitrogen Molecular Probes) and (Alexa Fluor 568 goat anti-Rabbit IgG (H + L); A-11011; Invitrogen by Thermo Fisher Scientific). Bound antibodies were visualized by a confocal laser scanning microscope (Leica TCS SPE) after staining of nuclei with DAPI (Cat. No. 62248; Thermo Fisher Scientific). Co-localization analysis of GRK2 or TOMM40 with PHF-tau on frontal cortex specimens was performed (*n* = 3 mice per group) by an observer who was blinded to genotype and treatment by Fiji/ImageJ (ImageJ 1.54p).

#### Immunohistological detection of Aβ

Immunohistological analyses were performed with paraffin-embedded, coronal brain sections (8 μm, taken at 50 μm intervals, at least 10-15 sections per set) of brain specimens from different groups of 16-month-old and 18-month-old, female and male mice (double-transgenic Tg2576-*GRK2S670D*, Tg2576-*GRK2K220R*, Tg2576-*GRKInh*, Tg2576-*GRK2*, Tg2576-*ARRB1*, and Tg2576-*TOMM6* mice; single-transgenic Tg2576 mice, without and with 6 months of treatment with CPD10 or CPD57, as indicated). The group size was 6 mice per group (3 female and 3 male mice). Immunohistological detection of human Aβ was performed with anti-β-amyloid-specific antibody (clone BAM-10; A5213; ascites fluid, Sigma-Aldrich). After deparaffinization and rehydration of sections, antigen retrieval was performed with citrate buffer (10 mM citric acid, pH 6.0, supplemented with 0.05% Tween 20) by microwave heating for 15 min at 90°C. Sections were allowed to cool to room temperature, and were rinsed twice with PBS. Thereafter endogenous peroxidases were inactivated by incubation with 3.7% H_2_O_2_ in PBS for 10 min, followed by another washing step. Specimens were placed in blocking buffer (PBS with 5% [w/v] bovine serum albumin fraction V, 0.05% Tween 20, pH 7.4) for 1 h at 37°C. Thereafter the primary antibody (1:200 dilution, pre-absorbed to mouse serum proteins) in blocking buffer was applied for 1 h at 37°C, followed by three washing steps with PBS supplemented with 0.05% Tween 20 to remove unbound antibody. The peroxidase-conjugated secondary antibody (goat anti-mouse IgG, peroxidase-conjugated AffiniPure F(ab’)_2_ fragment, Fcgamma fragment specific, Cat. No. 115-036-071; Jackson ImmunoResearch Laboratories; dilution 1:500 in blocking buffer) was added for 1 h at 37°C, followed by additional washing steps with PBS. Bound antibody was visualized by an enzyme substrate reaction (DAB Enhanced Liquid Substrate System for Immunohistochemistry, Product D 3939, Sigma-Aldrich). Counterstaining with hematoxylin (HE; Mayer’s hemalum solution, Cat. No. 1.09249.0500, Merck KGaA) was performed as indicated. Paraffin-embedded sections were mounted in Poly-Mount Xylene (Polysciences Inc.) and imaged with a DMI6000 microscope equipped with a DFC420 and K3 camera (Leica). The area covered by Aβ plaques was determined in a blinded manner by quantitative image analysis with Fiji/ImageJ (ImageJ 1.54p) with at least six different mouse brains per study group.

All replicates of Aβ detection on brain specimens of 16-month-old mice (Tg2576, Tg2576-*GRK2S670D*, Tg2576-*GRK2K220R*, Tg2576-*GRKInh*) are shown in Figure S3E. Replicates of stained brain specimens of 18-month-old Tg2576, Tg2576-*GRK2* and Tg2576-*ARRB1* mice are shown in Figure S4C. Replicates of brain specimens of 18-month-old, untreated Tg2576-*TOMM6*, CPD10-treated Tg2576, CPD57-treated Tg2576, and untreated Tg2576 mice are shown in Figure S5A.

#### Quantification of Aβ peptides

Soluble and insoluble human Aβ peptide (Aβ1-40 and Aβ1-42) contents in transgenic mouse brains (hippocampus and frontal cortex) with expression of the human APPSwe transgene were quantified similarly as described,^21^ by ELISA test (human amyloid beta 40 ELISA kit KHB3481; human amyloid beta 42 ELISA kit KHB3441; Invitrogen by Thermo Fisher Scientific). The study used brain extracts isolated from the indicated 16-month-old or 18-month-old, female and male mice, i.e., Tg2576 mice, Tg2576-*GRK2S670D* mice, Tg2576-*GRK2K220R* mice, Tg2576-*GRKInh* mice, Tg2576-*GRK2* mice, Tg2576-*ARRB1* mice, Tg2576-*TOMM6* mice, CPD10-treated Tg2576 mice, and CPD57-treated Tg2576 mice. Frozen brains (hippocampus and frontal cortex specimens) were extracted sequentially by Tris-HCl extraction buffer to yield the soluble Aβ1-40 and Aβ1-42, followed by extraction of insoluble Aβ with 70% formic acid. First, frozen brains were pulverized under liquid nitrogen and homogenized (1:10 weight/volume ratio) in 20 mM Tris-HCl, 150 mM NaCl, pH 7.4, supplemented with 5 mM EDTA and protease inhibitor cocktail (Sigma). Then, samples were centrifuged at 100,000 × g for 60 min at 4°C, and the supernatant containing the soluble Aβ was collected, diluted in ELISA assay buffer and used for soluble Aβ quantification by ELISA according to the instructions of the manufacturer (Thermo Fisher Scientific). The pellet containing the insoluble Aβ was extracted with 70% formic acid by sonication. After centrifugation (100,000 × g, 60 min), the supernatant was collected, neutralized with 1 M Tris-HCl, pH 8, diluted with assay buffer, and analyzed by ELISA. The absorbances of known concentrations of Aβ1-40 and Aβ1-42 peptides in identical buffer as the brain extracts were used to calculate picograms of Aβ per mg wet weight of the original brain.

#### Determination of α-, β-, and γ-secretase

Activities of hippocampal α-secretase, β-secretase and γ-secretase were determined as described,^21^ with the microsomal membrane fractions prepared from hippocampal tissue homogenates of the indicated female and male mice. Nuclei were removed from hippocampal homogenates by centrifugation for 10 min at 800 × g, and the microsomal membranes in the supernatant were collected by centrifugation at 25,000 × g for 60 min at 4°C and resuspended in the indicated assay buffer. The assay buffer for the α-secretase activity test was 10 mM Tris-HCl, pH 7.5. The fluorogenic α-secretase substrate Ac-RE(EDANS)-VHHQKLVF-K(DABCYL)-R-OH (Cat. No. 565767; Calbiochem) was added at a final concentration of 20 μM, and the assay was incubated for 1 h at 37°C. The assay buffer for the β-secretase activity test was 50 mM sodium acetate (pH 4.5). The assay was incubated for 30 min at 37°C, after the addition of 10 μM of β-secretase substrate H-K(DABSYL)-SEVNLDAEFRQ(LY) (Cat. No. 565781; Calbiochem). The γ-secretase activity was determined by incubation for 2 h at 37°C with the γ-secretase substrate, NMA-GGVVIATVK(DNP)-DRDRDR-NH_2_ (Cat. No. 565764; Calbiochem), which was used at a concentration of 8 μM in assay buffer (50 mM Tri-HCl, pH 6.8, supplemented with 150 mM NaCl, 2 mM EGTA, and 0.25% CHAPSO). Fluorescence intensities (excitation wavelength 340 nm, emission wavelength 490 nm for the α-secretase substrate; excitation wavelength 430 nm, emission wavelength 520 nm for the β-secretase substrate; excitation wavelength 355 nm, emission wavelength 440 nm for the γ-secretase substrate) were determined with a LS55B spectrophotometer (PerkinElmer).

#### Quantification of CUMS-induced neuronal loss

CUMS-induced hippocampal neuronal loss was quantitated in 16-month-old, male Tg2576 mice with and without 8 weeks of CUMS (and with CPD10 treatment during the CUMS protocol) by direct binding assay with [^125^I]-labeled anti-NeuN antibody, similarly as described.^24^ Before the assay, labeling of the affinity-purified, BSA-free anti-NeuN antibody (1–10 μg) was performed with 0.1–0.5 mCi Na[^125^I] in polypropylene tubes, which had been precoated with 100 μg of Iodo-Gen in CHCl_3_. After 10 min of incubation at room temperature, the iodination reaction was stopped by removal of the [^125^I]-labeled anti-NeuN antibody from the polypropylene tube, followed by purification with Sephadex LH-20 column chromatography. The binding assay quantified hippocampal neuronal cell bodies with the [^125^I]-labeled anti-NeuN antibody (5 × 10^−8^ M, 1.0 μCi/point),^22^ in crude hippocampal homogenates (0.5 mg protein/ml, PBS supplemented with 10 mg/mL bovine serum albumin) prepared from 16-month-old Tg2576 mice subjected for 2 months to the CUMS protocol, 16-month-old Tg2576 mice without CUMS, and 16-month-old Tg2576 mice treated with CPD10 during the CUMS protocol. After removal of unbound radioactivity by washing steps and centrifugation, specifically bound radioactivity of [^125^I]-labeled anti-NeuN antibody to neuronal hippocampal cell bodies was determined by scintillation counting (Beckman Coulter LS6500). Bound [^125^I]-labeled anti-NeuN antibody is given as % of control, i.e., 16-month-old, untreated Tg2576 mice without CUMS, which was set to 100%.

#### Determination of 20S proteasome activity

Hippocampal and brain (hippocampus and frontal cortex) 20S proteasome activities were determined with hippocampal (or hippocampal and frontal cortex) specimens isolated on ice from 18-month-old, untreated Tg2576 mice and from 18-month-old Tg2576 mice treated for 14 days with CPD57, or from 18-month-old Tg2576 mice treated for 6 months without or with the indicated doses of CDP57 (female and male mice). After washing with PBS, hippocampal or brain tissue (30 mg) was homogenized in lysis buffer (20 mM Tris pH 7.5, 5 mM MgCl_2_; buffer wet-weight ratio 3:1). After centrifugation at 20 000 × g for 15 min at 4°C, the pellet was discarded, and the lysate was diluted with assay buffer (without or with 0.03% SDS for activation of the chymotrypsin-like activity) and samples (10–50 μg protein) were immediately used for proteasome activity determination with the fluorogenic substrates to determine the chymotrypsin-like activity (Suc-LLVY-AMC; Suc-Leu-Leu-Val-Tyr-7-amino-4-methylcoumarin), the trypsin-like activity (Boc-LRR-AMC; Boc-Leu-Arg-Arg-AMC), and the caspase-like activity (Z-LLE-AMC; Z-Leu-Leu-Glu-AMC) (final concentration 50 μΜ) according to the instructions of the assay (BML-AK740; Enzo Life Sciences; and UBPBio, Cat. J4120). The fluorescence was recorded (excitation/emission 360 nm/460 nm) in a microplate reader after an incubation for 15–30 min at 37°C. Background fluorescence was subtracted, which was determined by incubation of the lysate for 10 min with the 20S proteasome inhibitor epoxomicin (500 nM) or MG132 (100 μM) before addition of the fluorogenic proteasome substrate.

#### Cultivation of primary mouse cortical neurons

Primary cortical neurons were isolated from brains of fetal B6 mice as described.^62^ Fetuses (embryonic day E16-E18) were released from 3 to 4 euthanized pregnant mice, fetuses were decapitated, brains were isolated, and brain cortices were dissected in dissection buffer (0.1 M PBS supplemented with 0.6% glucose) under a dissection microscope. Brain cortices were digested with prewarmed trypsin solution (0.025% Trypsin/EDTA) at 37°C for 15 min, with gentle agitation of the tissue every 5 min. The trypsin was inactivated with prewarmed horse serum. Tissue pieces were allowed to settle down, the supernatant was aspirated, and DNase I (40 μg/mL) in dissection buffer was added, and incubated for 5–10 min at 37° with gentle tipping after 5 min. Cells were dissociated by pipetting with a P1000 pipette (PIPETMAN, Gilson), the remaining tissue pieces were allowed to settle for 2 min, and the supernatant with the dissociated neurons was transferred to a new tube. Cells were collected by centrifugation at 800 × g for 5 min, resuspended in prewarmed neuronal culture medium, and seeded on poly-D-lysine-coated (50 μg/mL) culture plates. Primary cortical neurons were kept in Neurobasal medium supplemented with 1% heat-inactivated horse serum, 2% B-27 Supplement (Gibco, Thermo Fisher Scientific), 0.5 mM glutamine, 33 mM glucose, and 1% penicillin-streptomycin for 72 h. After 72 h of *in vitro* culture in a humidified atmosphere at 37°C with 5% CO_2_, cells were transferred to serum-free Neurobasal medium, and 50% of the medium were replaced every 3–4 days. After 7–10 days in culture, primary cortical neurons were used for experiments, including addition of aggregated Aβ1-42 (20 μM) and the GRK2 inhibitor CMPD101 (10 μM; SML2784, Sigma-Aldrich), ACO2 measurements, and transfection by electroporation or lentiviral transduction of lentiviruses encoding TOMM6 or the phosphomimetic mutant, TOMM6-ST-DD, with exchange of GRK2 phosphorylation sites (S3-S4-T5-S9-S13-T17) for aspartates.

#### Mitochondrial aconitase (ACO2) activity

Mitochondrial aconitase activity of mitochondrial ACO2 in aged mouse hippocampi (from female and male mice of the indicated mouse lines) and fetal primary mouse neurons was determined by the coupled reaction of aconitase converting citrate to isocitrate and of isocitrate dehydrogenase converting isocitrate to alpha-ketoglutarate. The assay measures the absorbance of the generated NADPH at 340 nm (Aconitase assay kit, item no. 705502, Cayman chemical). One milliunit of aconitase is the amount of enzyme which catalyzes the formation of 1 nmol of isocitrate/min, at 25°C, pH 7.4. For aconitase activity measurement with crude hippocampal mitochondria, hippocampi were isolated, rinsed with PBS (pH 7.4), homogenized in 0.2 mL cold assay buffer (50 mM Tris-HCl, pH 7.4) for 50 mg tissue, and centrifuged at 800 × g for 10 min at 4°C. The supernatant was collected and centrifuged at 20,000 × g for 10 min at 4°C. The pellet was resuspended in ice-cold assay buffer by sonication for 20 s (protein concentration of 0.5–1 mg/mL) and used immediately for the aconitase assay (see below).

If indicated, aconitase was reactivated by adding Na_2_S and Fe^3+^ directly to the assay mixtures. To 90 μL of extract, 10 μL of DTT (0.5 mM) and 1 μL each of Na_2_S (20 mM) and ferrous ammonium sulfate (20 mM) were added and incubated for 40 min on ice. After reactivation, the ACO2 activity was measured immediately.

Before ACO2 activity measurement of primary cortical neurons from fetal mice (embryonic age: E16-E18), neurons were treated for 48 h with 20 μM of aggregated Aβ1-42 (previously aggregated in 0.1 M sodium phosphate buffer, pH 7.8, for 2 h at 37°C at a concentration of 350 μg/mL) in the absence and presence of increasing concentrations of CPD10 (1 nM–100 μM). At the end of the incubation period, cells were washed with ice-cold PBS, collected and homogenized in 100 μL of ice-cold assay buffer (50 mM Tris-HCl, pH 7.4) buffer. The lysate was centrifuged at 800 × g for 10 min at 4°C. The supernatant was collected and centrifuged at 20,000 × g for 15 min at 4°C. The pellet was suspended by sonication in 0.1 mL of ice-cold assay buffer (at a protein concentration of 0.5–1 mg/mL) and immediately used for the aconitase assay.

The reaction was started by adding the mitochondrial extract (10–20 μg of protein) to the reaction buffer and measuring the increase in absorbance at 340 nm (at 22°C) in a 0.2 mL reaction mixture containing 50 mM Tris-HCl, pH 7.4, 5 mM sodium citrate, 0.5 mM MnCl_2_, 0.2 mM NADP+, and 2 units/ml of isocitrate dehydrogenase. The increase in fluorescence intensity was measured (excitation wavelength 340 nm, emission wavelength 430 nm), and the reaction rate was calculated with the extinction coefficient of NADPH at 340 nm (6200 M^−1^ cm^−1^). As indicated, the aconitase was reactivated as detailed above.

#### Hippocampal mitochondrial ATP contents

Hippocampal mitochondrial ATP contents were determined in female and male, 12- and 18-month-old, untreated Tg2576 mice and non-transgenic B6 mice, in 18-month-old Tg2576 mice after treatment for six months with CPD10 or CPD57, and in 18-month-old, untreated Tg2576-*ARRB1* and Tg2576-*TOMM6* mice. After hippocampus isolation from the brain on ice, the crude mitochondrial pellet was rapidly prepared on ice as detailed above. A small fraction was taken and immediately used for determination of mitochondrial ATP contents, following the instructions of the kit (ATP determination kit, Catalog number A22066; Thermo Fisher Scientific).

#### Chemical synthesis of CPD10

CPD10 (1-(1,3-benzodioxol-5-yl)-4-(cyclopropanecarbonyl)-3-hydroxy-2-phenyl-2H-pyrrol-5-one) was synthesized by ChiroBlock GmbH (Wolfen, Germany) with a purity of >98%. First, methyl 2-(1,3-benzodioxol-5-ylamino)-2-phenyl-acetate was synthesized by refluxing (in combination with a Dean-Stark apparatus) a mixture of methyl 2-oxo-2-phenyl-acetate (96 g, 583 mmol, 2.0 eq), 1,3-benzodioxol-5-amine (40g, 292 mmol, 1.0 eq), and TsOH (0.5 g, 3 mmol, 0.01 eq) in toluene (1500 mL) under N_2_ for 4 h. Thereafter, 5% Pd/C (12 g) was added, and hydrogenation of the obtained suspension was performed at 20 bar and 20°C for 48 h. The resulting heterogeneous mixture was filtered through Celite, and the filtrate was concentrated in vacuo. The resulting brown oil was purified by flash chromatography (silica gel, ethyl acetate - petroleum ether 1:5) to yield methyl 2-(1,3-benzodioxol-5-ylamino)-2-phenyl-acetate as a brown solid (38 g, purity 95%, yield: 46%). In the second step, S-*tert*-butyl ethanethioate was synthesized by cooling a solution of pyridine (144 g, 1829 mmol, 1.1 eq) in chloroform (660 mL) in an ice bath and treatment with acetyl chloride (144 g, 1829 mmol, 1.1 eq), with the reaction temperature not exceeding 10°C. To the resulting orange suspension, 2-methylpropane-2-thiol (150 g, 1663 mmol, 1.0 eq) was dropwise added over 40 min. The mixture was stirred for 48 h and subsequently quenched with water (900 mL). After phase separation, the aqueous phase was extracted with chloroform (600 mL), and the combined organic extracts were washed with saturated NaHCO_3_ (500 mL) and dried over Na_2_SO_4_. The resulting chloroformic solution was subjected to fractional distillation, which afforded target S-*tert*-butyl ethanethioate as a colorless liquid (181 g, purity 95%, yield 82%). In the next step, S-(2-pyridyl) cyclopropanecarbothioate was synthesized by the dropwise addition of cyclopropanecarbonyl chloride (47.0 g, 449 mmol, 1.0 eq.) to a solution of pyridine-2-thiol (50.0 g, 449 mmol, 1.0 eq) in THF (500 mL) at 20°C. Thereafter, the mixture was stirred for 10 min, filtered, and the filter cake was washed with 1:4 Et_2_O/petrol ether (2 × 250 mL). The resulting solid was dissolved in water (500 mL), and treated with NaHCO_3_ (37.7 g, 449 mmol, 1.0 eq). The aqueous solution was extracted with EtOAc (2 × 250 mL), and the organic fractions were combined, dried over Na_2_SO_4_, and concentrated in vacuo to yield S-(2-pyridyl) cyclopropanecarbothioate as yellow oil (74 g, purity 95%; yield 92%). Thereafter, S-*tert*-butyl 3-cyclopropyl-3-oxo-propanethioate was synthesized. For the synthesis, a 2-L 3-neck round-bottom flask was charged with HMDS (112.5 g, 669 mmol, 2.5 eq) and freshly distilled THF (1500 mL). The resulting mixture was cooled in an acetone/dry ice bath. Then, 1.6 M nBuLi in hexanes (436 mL, 697 mmol, 2.5 eq) was added dropwise while the temperature was kept below −50°C. The resulting mixture was first treated with a solution of S-(2-pyridyl) cyclopropanecarbothioate (50.0 g, 279 mmol, 1.0 eq) and then with S-*tert*-butyl ethanethioate (38.4 g, 290 mmol, 1.04 eq). Thereafter, the resulting solution was stirred at −30°C for 1 h, and the reaction was quenched by 2 N HCl (840 mL). The resulting suspension was extracted with ethyl acetate (3 × 400 mL), and the organic fractions were combined, dried over Na_2_SO_4_, and concentrated in vacuo. The product was purified by flash chromatography (silica gel, diethyl ether) to yield S-*tert*-butyl 3-cyclopropyl-3-oxo-propanethioate as a red oil (54.6 g, purity 70%, yield: 68%). Then, the synthesis of methyl 2-[1,3-benzodioxol-5-yl-(3-cyclopropyl-3-oxo-propanoyl)amino]-2-phenyl-acetate was performed by charging a 1-L round-bottom flask with methyl 2-(1,3-benzodioxol-5-ylamino)-2-phenyl-acetate (50 g, 175 mmol, 1.0 eq), S-*tert*-butyl 3-cyclopropyl-3-oxo-propanethioate (54.7 g, 273 mmol, 1.55 eq), CF_3_COOAg (56.1 g, 254 mmol, 1.45 eq), and distilled THF (1300 mL). The obtained mixture was stirred for 3 h at 20°C. The process was controlled by TLC. The dark-brown reaction mixture was concentrated in vacuo, purified by flash chromatography (silica gel, ethyl acetate – dichloromethane 1:9) to yield methyl 2-[1,3-benzodioxol-5-yl-(3-cyclopropyl-3-oxo-propanoyl)amino]-2-phenyl-acetate as a brown oil (50.1 g, purity: 90%, yield 65%). In the last step, the synthesis of 1-(1,3-benzodioxol-5-yl)-4-(cyclopropanecarbonyl)-3-hydroxy-2-phenyl-2H-pyrrol-5-one (CPD10) was performed in a 1000 mL round-bottom flask, which was charged with methyl 2-[1,3-benzodioxol-5-yl-(3-cyclopropyl-3-oxo-propanoyl)amino]-2-phenyl-acetate (50 g, 126 mmol, 1.0 eq), CsF (19.2 g, 126 mmol, 1.0 eq), and DMF (500 mL). The mixture was stirred for 20 h at 60°C. The process was controlled by TLC. Thereafter, the dark-brown reaction mixture was concentrated in vacuo, treated with 2 N aq. HCl (500 mL), and extracted with EtOAc (500 mL). The organic phase was washed with brine (2 × 300 mL), dried over Na_2_SO_4_, and concentrated in vacuo to yield crude 1-(1,3-benzodioxol-5-yl)-4-(cyclopropanecarbonyl)-3-hydroxy-2-phenyl-2H-pyrrol-5-one as a brown solid. The crude solid product was washed with EtOAc until the colorless target product, 1-(1,3-benzodioxol-5-yl)-4-(cyclopropanecarbonyl)-3-hydroxy-2-phenyl-2H-pyrrol-5-one (CPD10) was obtained as an off-white solid (20.0 g, purity 95%, yield 43%). Obtained products from two batches were recrystallized from EtOAc to yield target CPD10 (25.2 g, purity 99.2%). MS (m/z): 364 [M + H]^+^. ^1^H-NMR (500.13 MHz; DMSO) δ 1.21–1.27 (m, 4H), 2.98 (s, 1H), 5.76 (s, 1H), 5.96–5.98 (m, 2H), 6.82–6.84 (d, 1H, J = 10.0 Hz), 6.95–6.95 (m, 1H), 7.22–7.23 (m, 1H), 7.26–7.32 (m, 5H).

#### Chemical synthesis of NH2-CPD10

The chemical synthesis of 2-(4-aminophenyl)-1-(1,3-benzodioxol-5-yl)-4-(cyclopropanecarbonyl)-3-hydroxy-2H-pyrrol-5-one (NH2-CPD10) was performed by ChiroBlock GmbH (Wolfen, Germany). A mixture of methyl 2-(4-nitrophenyl)-2-oxo-acetate (100 g, 478 mmol, 1.0 eq), 1,3-benzodioxol-5-amine (65 g, 478 mmol, 1 eq), and TsOH (0.9 g, 5 mmol, 0.01 eq) in toluene (1000 mL) was refluxed with a Dean-Stark apparatus under N2 for 2h. Then toluene was evaporated and the residue was dissolved in dichloromethane (1000 mL) and methanol (1000 mL). After addition of acetic acid (27.3 mL, 478 mmol, 1.0 eq) the mixture was cooled to 5°C. Thereafter, sodium cyanoborohydride (30 g, 478 mmol, 1.0 eq) was added over 30 min, and the obtained suspension was stirred at 20°C for 20 h. The resulting mixture was filtered through a 10 cm pad of silica gel. The filtrate was concentrated in vacuo to yield an orange-brown solid of methyl 2-(1,3-benzodioxol-5-ylamino)-2-(4-nitrophenyl)acetate (120 g, purity 95%, yield 76%). In the next step, a mixture of methyl 2-(1,3-benzodioxol-5-ylamino)-2-(4-nitrophenyl)acetate (20 g, 61 mmol, 1 eq) and pyridine (9.8 mL, 121 mmol, 2 eq) in 200 mL of dichloromethane was cooled to −70°C. Trifluoroacetic anhydride (9.2 mL, 67 mmol, 1.1. eq) was added, the mixture was warmed to room temperature and stirred for 3 h. Then the mixture was washed with aqueous HCl (2 N, 36 mL, 1.2 eq), dried (5 g Na_2_SO_4_) and concentrated in vacuo to yield a brown oil. The crude product was further purified by flash chromatography (silica gel, ethyl acetate-petroleum ether 1:3) to yield methyl 2-[1,3-benzodioxol-5-yl-(2,2,2-trifluoroacetyl)amino]-2-(4-nitrophenyl)acetate as an orange oil (19.9 g, purity: 95%, yield 77%). In the next step, a solution of Na_2_S_2_O_4_ (46.5 g, 267 mmol, 6 eq) and NaHCO_3_ (48.6 g, 579 mmol, 13 eq) in water (210 mL) was added at 20°C to a solution of methyl 2-[1,3-benzodioxol-5-yl-(2,2,2-trifluoroacetyl)amino]-2-(4-nitrophenyl)acetate (19 g, 45 mmol, 1 eq) in tetrahydrofuran (525 mL). After stirring for 1 h under process control by thin-layer chromatography (EtOAc:petroleum ether 1:3, UV-, ninhydrin detection), di-*tert*-butyldicarbonate (19.4 g, 89 mmol, 2 eq) was added. The mixture was stirred for 4 days at 20°C. Thereafter, the reaction mixture was partitioned between water (200 mL) and ethyl acetate (200 mL) and the layers were separated. The organic layer was dried (5 g Na_2_SO_4_) and evaporated, and the resulting crude product was purified by flash chromatography (silica gel, ethyl acetate - petroleum ether 1:3) to yield methyl 2-[1,3-benzodioxol-5-yl-(2,2,2-trifluoroacetyl)amino]-2-[4-(2-*tert*-butoxy-2-oxo-ethyl)phenyl]acetate as a beige solid (5.3 g, purity: 95%, yield: 24%). Thereafter, an aqueous solution of ammonia (35% in H_2_O, 28 mL, 50 eq) was added to a solution of methyl 2-[1,3-benzodioxol-5-yl-(2,2,2-trifluoroacetyl)amino]-2-[4-(2-*tert*-butoxy-2-oxo-ethyl)phenyl]acetate (5 g, 10 mmol, 1 eq) in methanol (280 mL) at 20°C. The mixture was stirred for 4 days under process control by thin-layer chromatography (EtOAc:dichloromethane 1:50, UV detection). The reaction mixture was evaporated, and the crude product was purified by flash chromatography (silica gel, ethyl acetate – dichloromethane 1:50) to yield methyl 2-(1,3-benzodioxol-5-ylamino)-2-[4-(2-*tert*-butoxy-2-oxo-ethyl)phenyl]acetate as a beige solid (1.7 g, purity 95%, yield 42%). In the next step, CF_3_COOAg (1.4 g, 6.5 mmol, 1.5 eq) was added to a solution of methyl 2-(1,3-benzodioxol-5-ylamino)-2-[4-(2-*tert*-butoxy-2-oxo-ethyl)phenyl]acetate (1.72 g, 4.3 mmol, 1.0 eq) and *S*-*tert*-butyl 3-cyclopropyl-3-oxo-propanethioate (1.3 g, 6.5 mmol, 1.5 eq) in distilled tetrahydrofuran (65 mL), and the obtained mixture was stirred at 20°C for 3 h under process control by thin-layer chromatography (EtOAc:petroleum ether 1:1, detection UV). The resulting dark brown reaction mixture was concentrated in vacuo and purified by flash chromatography (silica gel, ethyl acetate - dichloromethane 1:9) to yield methyl 2-[1,3-benzodioxol-5-yl-(3-cyclopropyl-3-oxo-propanoyl)amino]-2-[4-(2-*tert*-butoxy-2-oxo-ethyl)phenyl]acetate as a brown oil (2.9 g, purity 40%, yield: 71%). Thereafter, K_2_CO_3_ (1.2 g, 8.5 mmol, 1.5 eq) was added to a solution of methyl 2-[1,3-benzodioxol-5-yl-(3-cyclopropyl-3-oxo-propanoyl)amino]-2-[4-(2-*tert*-butoxy-2-oxo-ethyl)phenyl]acetate (2.9 g, 5.7 mmol, 1.0 equiv.) in tetrahydrofuran (100 mL), and the obtained mixture was stirred at 65°C for 12 h under process control by thin-layer chromatography (EtOAc, UV-detection). The dark-brown reaction mixture was concentrated in vacuo to yield crude *tert*-butyl N-[4-[1-(1,3-benzodioxol-5-yl)-4-(cyclopropanecarbonyl)-3-hydroxy-5-oxo-2H-pyrrol-2-yl]phenyl]carbamate as a brown solid. The crude product was further purified by flash chromatography (silica gel, ethyl acetate-petroleum ether 1:1) to yield *tert*-butyl N-[4-[1-(1,3-benzodioxol-5-yl)-4-(cyclopropanecarbonyl)-3-hydroxy-5-oxo-2H-pyrrol-2-yl]phenyl]carbamate as a brown oil (2.37 g, purity: 30%, yield 44%). In the next step, trifluoroacetic acid (5 mL, 65 mmol, 13 eq) was added to a solution of *tert*-butyl N-[4-[1-(1,3-benzodioxol-5-yl)-4-(cyclopropanecarbonyl)-3-hydroxy-5-oxo-2H-pyrrol-2-yl]phenyl]carbamate (2.37 g, 5 mmol, 1.0 eq) in dichloromethane (50 mL), and the mixture was stirred for 12 h at 20°C under process control by thin-layer chromatography (EtOAc:MeOH 9:1, UV-detection). The reaction mixture was concentrated in vacuo to afford crude 2-(4-aminophenyl)-1-(1,3-benzodioxol-5-yl)-4-(cyclopropanecarbonyl)-3-hydroxy-2H-pyrrol-5-one. The crude product was purified 3 times by flash chromatography (silica gel, ethyl acetate – methanol 9:1) to yield target 2-(4-aminophenyl)-1-(1,3-benzodioxol-5-yl)-4-(cyclopropanecarbonyl)-3-hydroxy-2H-pyrrol-5-one as a brown solid (0.244 g, purity: 70.4% [HPLC], yield 6%). MS (ESI) (m/z): 377 [M-1] negative mode (formula weight 378.39 [g/mol]; exact mass 378). ^1^H-NMR (500.13 MHz; MeOD) d 0.86–0.88 (m, 3.8H), 1.07 (s, 1.6H), 3.75 (m, 1.1H), 5.08 (s, 0.5H), 5.84 (s, 2.3H), 6.79–6.81 (m, 4.0H), 7.09–7.11 (m, 3.0H).

#### Chemical synthesis of CPD57

CPD57 (4-(4-fluorophenyl)-6-(hydroxymethyl)-2-methyl-pyridine-3-carboxamide) was synthesized by ChiroBlock GmbH (Bitterfeld-Wolfen, Germany) with a purity of >97%. First, (E)-4-(4-fluorophenyl)-2-oxo-but-3-enoic acid was synthesized. To an ice-cold mixture of 4-fluorobenzaldehyde (240 g, 1.9 mol, 1 eq) and 2-oxopropanoic acid (170 g, 1.9 mol, 1 eq) in 1600 mL methanol, a solution of KOH (191 g, 2.9 mol, 1.5 eq) in 300 mL water was slowly added dropwise. This exothermic reaction was kept below 25°C, and the addition took approx. 75 min. The resulting yellow suspension was cooled to 5°C overnight. The yellow solid was filtered off and washed once with 450 mL of cold MeOH. The crude residue was taken up in 1.2 L of water and the pH value was adjusted to 1 by conc. HCl (162 mL, 1.9 mol, 1 eq). The resulting suspension was extracted with DCM (3 L), the organic phase was dried over anhydrous sodium sulfate and evaporated to dryness in vacuo. The resulting solid was washed with petroleum ether (500 mL) and dried at 60° in vacuo to yield (E)-4-(4-fluorophenyl)-2-oxo-but-3-enoic acid as pale-yellow solid (112.68 g, purity: 95%, yield 30%). In the next step 5-cyano-4-(4-fluorophenyl)-6-methyl-pyridine-2-carboxylic acid was synthesized. Therefore, KOtBu (280 g, 2.5 mol, 3 eq) was added slowly to an ice-cooled solution of (E)-4-(4-fluorophenyl)-2-oxo-but-3-enoic (161 g, 0.831 mol, 1 eq) in 4.5 L CH_3_CN. Because of the exothermic reaction, the temperature was kept below 30°C, and the addition took approx. 65 min. The resulting red-brown gelatinous suspension was stirred at 23°C for 42 h. The solvent was removed under reduced pressure and the residue was taken up in 4.8 L water. The brown solution was extracted with TBME (2 × 1.2 L) and then acidified with 2.1 L of 2N HCl. The resulting suspension was filtered off and the crude product was purified by crystallization from 800 mL of CH_3_CN. The precipitate was collected by filtration and dried at high vacuum to yield 5-cyano-4-(4-fluorophenyl)-6-methyl-pyridine-2-carboxylic acid as beige solid (60.81 g, purity: 95%, yield: 29%). In the next step, 5-carbamoyl-4-(4-fluorophenyl)-6-methyl-pyridine-2-carboxylic acid was synthesized. To this end, KOtBu (162.4 g, 1.4 mol, 3 eq) was added to a suspension of 5-cyano-4-(4-fluorophenyl)-6-methyl-pyridine-2-carboxylic acid (123.62 g, 0.482 mol, 1 eq) in water (1.2 L). The mixture was stirred at 100°C for 3 h. After cooling down to 20°C, the resulting brown solution was acidified by 2 N HCl (780 mL, 3.23 eq). The precipitated product was collected by filtration, washed with water (140 mL) and dried at high vacuum for 24 h to yield 5-carbamoyl-4-(4-fluorophenyl)-6-methyl-pyridine-2-carboxylic acid (109.57 g, purity: 95%, yield: 83%). Thereafter, ethyl chloroformate (8.3 mL, 87.5 mmol, 1.2 eq) was added slowly at 5°C during 30 min to a mixture of 5-carbamoyl-4-(4-fluorophenyl)-6-methyl-pyridine-2-carboxylic acid (20 g, 73 mmol, 1 eq) and triethylamine (12.3 mL, 87.5 mmol, 1.2 eq) in absolute THF (560 mL). The resulting suspension was warmed to 20°C, cooled to 5°C and then added slowly to a solution of NaBH_4_ (5.5 g, 149.9 mmol, 2 eq) in 80 mL of water at 5°C. The brown solution was stirred at 20°C for 1h, neutralized by the addition of 1 N HCl (75 mL) and concentrated in vacuo. The resulting suspension was extracted by ethyl acetate (1 × 700 mL, 3 × 250 mL). The combined organic phases were dried over anhydrous sodium sulfate and evaporated to dryness in vacuo to obtain crude 4-(4-fluorophenyl)-6-(hydroxymethyl)-2-methyl-pyridine-3-carboxamide (CPD57) (∼15 g).

Crude material of CPD57 from 3 batches (∼45 g) were further purified by flash chromatography (silica gel, EE:MeOH 5:1). The resulting 90–95% pure CPD57 (20.15 g) was finally crystallized in ethanol/water 1:1 (500 mL) to yield 17.73 g of 4-(4-fluorophenyl)-6-(hydroxymethyl)-2-methyl-pyridine-3-carboxamide (CPD57) with a purity of >97% (yield 31%). MS (m/z): 261 [M + H]^+^. ^1^H-NMR (500.13 MHz; DMSO) δ 2.49–2.50 (d, 3H, J = 5.0 Hz), 4.57–4.58 (d, 2H, J = 5.0 Hz), 5.43–5.45 (t, 1H, J = 5.0 Hz), 7.24 (s, 1H), δ 7.28–7.32 (m, 2H), δ 7.50 (s, 1H), δ 7.52–7.55 (m, 2H), 7.80 (s, 1H).

#### Computational docking of TOMM6 and TOMM6-ST-DD

Computational docking of TOMM6 and of the phosphomimetic mutant TOMM6-ST-DD was performed to define the propensity for formation of TOMM6 dimers and higher order oligomers and to elucidate the impact of GRK2-mediated phosphorylation on TOMM6 oligomerization. All computational docking studies were performed with Biovia Discovery Studio 2024 (v24.1.0.23298, Dassault Systèmes Biovia Corp.). Computational docking (ZDOCK algorithm) used the AlphaFold monomer model of TOMM6 (AF-Q96B49-F1-v4) and of the phosphomimetic TOMM6-ST-DD mutant (S3D-S4D-T5D-S9D-S13D-T17D), in which all GRK2 phosphorylation sites were exchanged to aspartate (D). The obtained dockings were refined using the RDOCK algorithm and the scoring function RDOCK energy (E_RDOCK). Default settings were used for docking. The top three dimers, according to the E_RDOCK scoring function, are shown for the dimer (Figure 2J). For the top three dimers, the docking analysis was performed (dimer with itself), yielding top three tetramers for each dimer (total of 9 tetramers). For these 9 tetramers, docking was performed (tetramer with itself), yielding octamers. For these top 9 octamers, E_RDOCK values are reported in Figure 2J, one value for each tetramer-tetramer docking complex.

#### Conceptual model of GRK2 function modulation by CPD10

The conceptual model of GRK2 function modulation by CPD10 shown in Figure 5I was created by using the crystal structures of the (p-S670)-GRK2 tetramer (PDB ID: 1YM7; ref.^37^) and the TOMM6 monomer (PDB ID 7CK6; ref.^25^). Missing N- and C-terminal residues of TOMM6 were added from the AlphaFold structure of TOMM6 (AF-Q96B49-F1-v4). Dimer and tetramer docking yielding the TOMM6 octamer was performed with ZDOCK (Biovia Discovery Studio 2024 v24.1.0.23298, Dassault Systèmes Biovia Corp.).

#### Computational docking studies of CPD10 and CPD57

All computational docking studies were performed with Biovia Discovery Studio 2024 (v24.1.0.23298, Dassault Systèmes Biovia Corp.). Data of the protein crystal structures, human GRK2 and the human 20S proteasome, were directly retrieved from the RCSB Protein DataBank (PDB ID: 3CIK^35^; PDB ID: 8QYM^44^). The PDB files were loaded into the program (Biovia Discovery Studio 2024) and prepared for docking studies by addition of hydrogen to satisfy any unsatisfied valences, and by removal of alternate amino acid conformations. The input of missing loop regions was conducted based on SEQRES data by DS Modeler with the Modeller algorithm.^63^ The final structure was protonated with the Protein Ionization method,^64^ and water molecules were removed.

The compounds, CPD10 and CPD57, were first drawn in 2D, transformed into 3D structures, and then used as a ligand. We used a CHARMm-based molecular dynamics (MD) scheme to dock ligands into a receptor binding site. For initial ligand placement, we defined 23 binding spheres in the GRK2 structure (PDB 3CIK) with modeled C-terminus, which had been identified by the LigFit algorithm.^65^ The serine-670-phospho-GRK2 structure with modeled C-terminus showed 21 binding spheres. We identified one binding sphere in GRK2, which was specific for GRK2 (H280, L283, L318, K319, P320, A321, N322, T353, H354, G355, Y356, M380, K383, G387, H388, S389, R392, I402), because docking of CPD10 to this binding sphere of phospho-S670-GRK2 failed. Moreover, we also identified one binding sphere in phospho-S670-GRK2, to which CPD10 was favorably docked (I197, G198, V205, Y206, A218, M219, K220, E239, V255, C256, F269, L271, D272, L273, M274, N275, G277, D278, N322, L324, L325, R332, I333, S334, D335). This phospho-S670-GRK2-specific binding sphere was not identified in non-phosphorylated GRK2 (without and with modeled C-terminus). This binding sphere was specific for GRK2 with 8 specific amino acids (of 25), which were not conserved in other members of the GRK family or in PKA.^35^

For initial ligand placement of the proteasome enhancer (CPD57), 113 binding spheres were identified in the human 20S proteasome assembly intermediate structure 3 (PDB 8QYM). The proteasome enhancer CPD57 was docked to a binding sphere located in the 20S proteasome gate of the human 20S proteasome assembly intermediate structure 3 (PDB 8QYM), contacting PSMA3 and PSMA1. The interaction involves a carbon oxygen hydrogen bond between E94 in PSMA3 and CPD57 (Figure 7A), which is an unconventional but common and important type of interaction in biological structures.^66^ The binding sphere encompasses the following residues of PSMA3: F68, N69, V79, L82, A83, D84, A85, R86, S87, L88, A89, D90, I91, A92, R93, E94, E95, S96, Y119, Y123, F132. The following residues of PSMA1 are also part of the binding sphere: V66, D67, H69, I70, I72, D80, A81, R82, L83, L84, C85, N86, F87, M88, R89, Q90, E91, C92, L93, D94, S95, R96, F97, V98, F99, R101, P102, L103, P104, V105, S106, R107, L108, V109, S110, L111, I112, G113, S114, K115, T116, Q117, I118, P119, T120, Q121, R122, R125, P127, Y128, V130, L132, I134, D138, F145, Q146, T147, C148, P149, S150, A151, N152, Y153, F154.

For docking studies, 10 starting random conformations of the ligand were generated using high temperature MD (dynamic target temperature of 1000K, with 1000 dynamic steps) including electrostatics. For each conformation, 10 random rigid body rotations were generated and tested for simulated annealing (with 2000 heating steps to reach heating target temperature of 700K, and 5000 cooling steps to reach cooling target temperature of 300K). Docking used the CHARMm forcefield and the ligand partial charge method.^67^ Grid extension was set to 8.0 Å beyond ligand bounds. By this approach candidate poses were found, which were subjected to final minimization for refinement. After performing the CDOCKER alignment, CDOCKER scores were calculated for each pose based on typing of receptor and ligand with a CHARMm forcefield.^68^ The 10 poses with the lowest CDOCKER interaction and CDOCKER binding energy were determined,^69^ and saved as “Top Hits”.

The scoring algorithm was completed by applying several other scoring functions, a method which improves reliability of the process and its results because each function focuses on other aspects of the binding. The first set of scoring functions used included the Jain scoring function,^70^ the Ligscore 1 and 2 scoring functions,^71^ and the LUDI function,^72^^,^^73^ which are empirical scoring functions and rely on analysis of binding affinity data between receptor and ligand. The LigScore 1 and 2 functions employed the Dreiding forcefield for their calculations.^74^ A second set of scoring functions which was applied, consisted of the Piecewise Linear Potential, PLP,^75^ and the Potential of Mean Force (PMF/PMF04).^76^^,^^77^ PLP is a simple function, which considers only steric interactions and hydrogen bond interactions between receptor and ligand, while PMF uses a knowledge-based statistical method relying on database data and distances between interacting atom pairs of the receptor (GRK2, 20S proteasome) and the ligand (CPD10, CPD57).

After conducting docking and performing scoring of the resulting poses, we calculated the binding energies between the receptor (GRK2, p-S670-GRK2) and its ligand (CPD10), and between the 20S proteasome assembly intermediate structure 3 (receptor) and its ligand (CPD57). The binding energy in kcal/mol is derived by subtracting the energy of the receptor and the energy of the ligand from the energy of the ligand-receptor complex. Flexible receptor atoms are identified in the workflow of the docking procedure, and receptor-ligand interactions are calculated accordingly. No implicit solvent model and no explicit solvent model was used to calculate binding energies. Translational and rotational entropy were not included in the calculation of the binding energy between receptor and ligand to improve the accuracy of the calculation. *In situ* ligand minimization resulted only in minor changes in binding energy.

#### Homology modeling of the C-terminus of GRK2

Because the C-terminal region of GRK2 (S670-L689) including serine-670 is not resolved in crystal structures of GRK2, we performed homology modeling of residues S670-L689 of GRK2. Homology modeling was performed with Biovia Discovery Studio Studio 2024 (v24.1., Dassault Systèmes Biovia Corp.). Homology modeling was based on protein domain structures, which showed homology to the C-terminal GRK2 region. An RCSB PDB database search identified that residues A581-G622 of polyribonucleotide nucleotidyltransferase 1, PNPT1 (PDB entry 3U1K) showed homology to residues A650-G688 of GRK2, and residues E261-A273 of homoserine kinase (PDB entry 2PPQ) were homologous to residues E674-A686 of GRK2. Based on the structural elements of both proteins, the C-terminus of GRK2 was modeled as a short helix followed by a beta sheet-like (coil-turn-coil) structure: Ser670-Lys677 (helix), Val678-Gly684 (coil), Ser685-Asn687 (turn) and Gly688-Leu689 (coil). In phospho-GRK2, serine-670 was replaced by phospho-serine-670. The Psi and Phi angles were as follows: Ser670 or phospho-Ser670 (−47; −57); Pro671 (−47; −57); Val672 (−47; −57); Val673 (−47; −57); Glu674 (−47; −57); Leu675 (−47; −57); Ser676 (−47; −57); Lys677 (−47; −57); Val678 (120; −120); Pro679 (120; −71); Leu680 (120; −120); Val681 (120; −120); Gln682 (120; −120); Arg683 (120; −120); Gly684 (120; −120); Ser685 (−47; −57); Ala686 (−47; −57); Asn687 (−47; −57); Gly688 (120; −120); Leu689 (--; −120).

#### Molecular dynamics simulation of effects of CPD10 on GRK2 and phospho-S670-GRK2

Ligand-induced changes in total energy of GRK2 and phospho-S670-GRK2 were determined by molecular dynamics (MD) simulations using the CHARMm forcefield (Biovia Discovery Studio Studio 2024; v24.1., Dassault Systèmes Biovia Corp.). The MD simulation strategy was adapted from a previously published protocol, which was established for proteins to discriminate correct and misfolded protein conformations.^36^ Initially, after cleaning of geometry, energy minimization was performed with the Smart Minimizer Algorithm without implicit solvent model, with 1000 steps of Steepest Descent with an RMS gradient tolerance of 3, followed by Conjugate Gradient minimization. After energy minimization, MD simulation was performed with the Standard Dynamics Cascade and the Generalized Born implicit solvent model, with a simulation time of 4 ps, a time step of 2 fs, an initial temperature of 50 K and a target temperature of 300 K. The velocity frequency was adjusted to 50. Total energies are reported after minimization (Potential CHARMm Energy in kcal/mol, without implicit solvent model) and after MD simulation plus minimization (Potential Energy in kcal/mol, with implicit solvent model) for structures of GRK2 (PDB ID: 3CIK^35^ with modeled C-terminus S670-L689) before and after CPD10 binding, and of phospho-S670-GRK2 before and after CPD10 binding.

### Quantification and statistical analysis

Statistical details of experiments can be found in the figure legends. Data are from individual mice or represent biological replicates if not otherwise indicated and are shown as mean ± s.d. Immunoblot data were quantitated after scanning by image analysis with Fiji/ImageJ (ImageJ 1.54p) and normalized to the respective loading control, detecting a housekeeping protein. Quantitative evaluation of immunofluorescence images and of immunohistological sections was performed by Fiji/ImageJ (ImageJ 1.54p) by an observer who was blinded to genotype and treatment. All data were tested for normality by the Shapiro-Wilk test. After normality test was passed (Shapiro-Wilk test, alpha = 0.05), *p*-values were calculated with the unpaired *t* test. One-way analysis of variance (ANOVA) was performed for comparisons between more than two groups followed by a post-hoc test (Tukey’s test for comparisons between all possible pairs of groups, and Dunnett’s test for comparisons against a control group). For comparisons of groups with unequal variances, the Welch’s ANOVA test was used. The t-values and degrees of freedom are shown for t-tests, and F-values and degrees of freedom (df) are shown for ANOVAs. Statistical significance was set at a *p*-value of <0.05 unless otherwise stated. Probability of survival of different transgenic mouse lines was determined by Kaplan-Meier survival curves with log rank (Mantel-Cox) test. Statistical data analysis was performed with Prism version 10.4.1 (GraphPad Software, Inc.) and R for macOS (R Foundation). All experimental data were repeated at least three times with similar results. Treatment and control groups were assigned by the researcher based on genotype. Mice with (healed) bite wounds were excluded. No randomization was performed. Sample size of animal experiments was predefined by power analysis which applied a linear mixed model test (Hotelling Lawley Trace test),^78^ with a target power of 0.8 and a type I error rate of 0.05. Outcome variables for sample size determination were Aβ plaque accumulation, changes in protein (e.g., GRK2, ARRB1, TOMM6) and phospho-S670-GRK2 contents in hippocampus, with sex and genotype as fixed nominal predictors and age as fixed continuous predictor. Genotype or treatment was considered as main effect. The expected effect sizes were estimated from previous studies with Tg2576 mice,^21^^,^^22^ and from the initial phenotyping data. The heatmap of Tg2576 mouse frontal cortex microarray gene expression profiling data was generated by Morpheus (https://software.broadinstitute.org/morpheus). The graphical abstract was drawn with Adobe Illustrator 2026, version 30.0 (Adobe Systems Software Ireland Limited). The overrepresentation analysis of whole genome microarray gene expression data with statistically significant up-regulation in the CPD10-treated Tg2576 group compared to the untreated control group (*p* < 0.01, and ≥2-fold up-regulation) was performed with g:GOSt of g:Profiler (g:Profiler version e113_e.g.,59_p19_f6a03c19, database updated on 23/05/2025). *p* values were determined with Fisher’s one-tailed test, and multiple testing correction was performed with G:SCS algorithm of g:GOSt50.

## Supplemental information

Document S1. Figures S1–S9 and Tables S1–S4

Table S5. Overrepresentation analysis of transcripts upregulated by CPD10 treatment in frontal cortices of Tg2576 mice (Table S5A) and list of significantly up-regulated genes that were used for the overrepresentation analysis (Table S5B)

Document S2. Article plus supplemental information
