# Supplementary material for: Analysis of GRK2 aggregation in the pathology of Alzheimer disease in animal models
Source: Cell Rep Med. 2026 Mar 26;7(4):102707. doi: 10.1016/j.xcrm.2026.102707 (PMC13130637; doi:10.1016/j.xcrm.2026.102707)
Supplement: Document S1. Figures S1–S9 and Tables S1–S4 [file mmc1.pdf]

Cell Reports Medicine, Volume 7

Supplemental information

Analysis of GRK2 aggregation in the pathology

of Alzheimer disease in animal models

Joshua Abd Alla, Alexander Perhal, Xuebin Fu, Andreas Langer, Yasser el Faramawy, and Ursula Quitterer

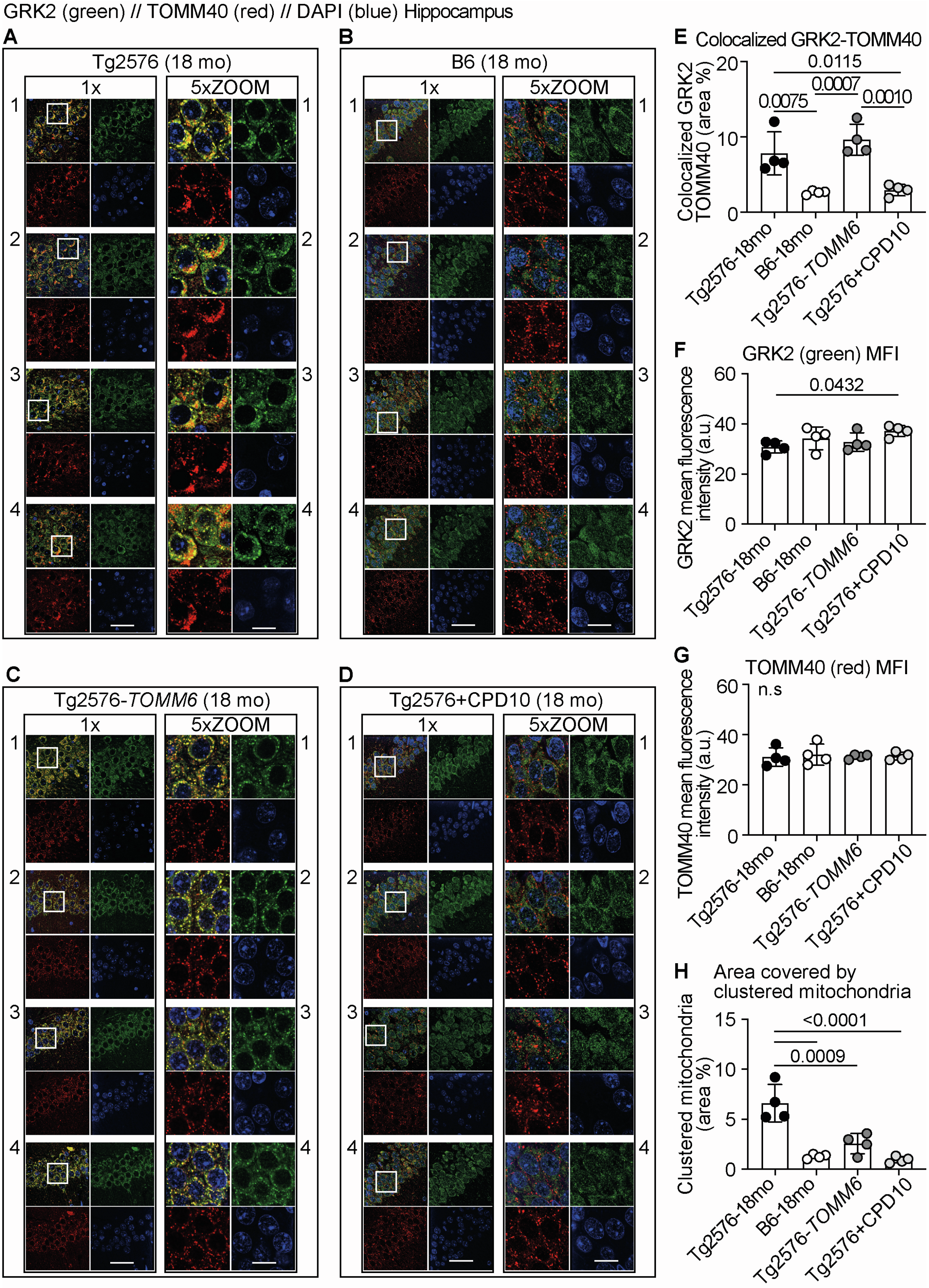

Figure S1. Immunofluorescence co-localization of GRK2 with the mitochondrial protein TOMM40 on hippocampal specimens of mice. Related to Figure 1, Figure 4, and Figure 6.

(A-D) Immunofluorescence co-localization of TOMM40 (red) with GRK2 (green) on hippocampal specimens of 18-month-old Tg2576 mice (A), 18-month-old, non-transgenic B6 mice (B), 18-month-old, double-transgenic Tg2576-*TOMM6* mice (C), and 18-month-old Tg2576 mice treated for 6 months with CPD10 (8mg/kg/d) (D). Nuclei were stained with DAPI (blue). Overview images of the CA1 hippocampal area are shown on the left (bar: 100 μm). The area marked by a white square frame is shown in a 5-fold higher magnification (ZOOM) on the right (bar: 20 μm). Sections were prepared from 4 different mice per group (No. 1-4, two females and two males). Representative immunofluorescence images of Tg2576 (A) and B6 (B) mice are shown in Figure 1B, and of Tg2576 (A) and Tg2576-*TOMM6* (C) mice are shown in Figure 4G.

(E) Quantitative analysis of GRK2 co-localization with mitochondrial TOMM40. The area covered by GRK2 colocalized with TOMM40 is shown (area %) and was determined by quantitative image analysis of hippocampal specimens (A-D).

(F,G) Mean fluorescence intensities (MFI) of GRK2 (F) and TOMM40 (G) on hippocampal sections shown in (A-D).

(H) The bar graph shows the area (%) covered by clustered mitochondria on hippocampal specimens shown in (A-D).

Data (E-H) represent mean ± s.d. (n = 4 mice per group). P-values were determined by one-way ANOVA and Tukey's test (E,G,H) and Dunnett's test (F); $F(3,12) = 15.08$ (E), 2.568 (F), 0.08613 (G), 23.23 (H).

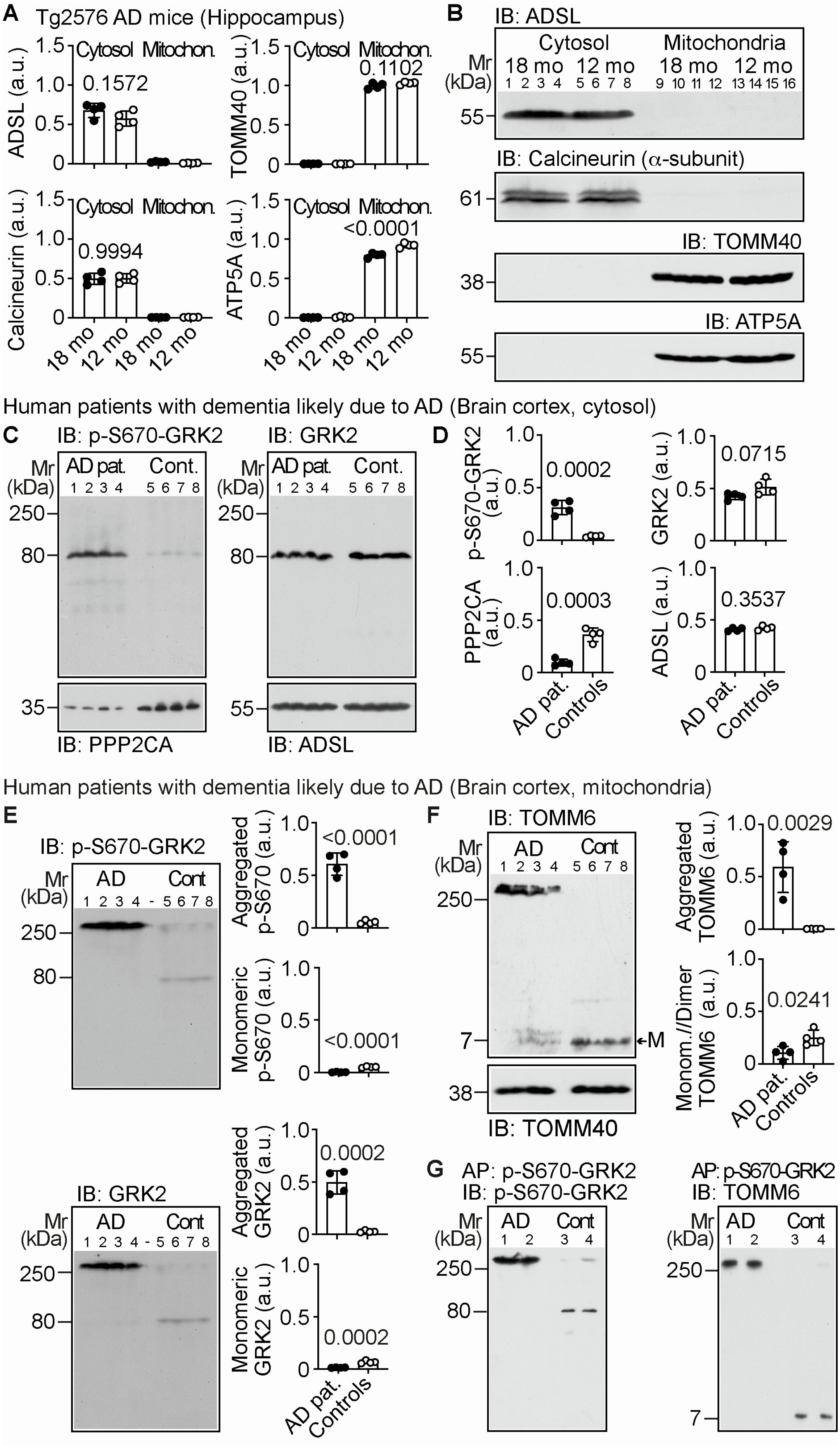
A
Tg2576 AD mice (Hippocampus)
ADSL (a.u.)
Cytosol
Mitochon.
0.1572
TOMM40 (a.u.)
0.1102
Calcineurin (a.u.)
0.9994
ATP5A (a.u.)
<0.0001
18 mo
12 mo
B
IB: ADSL
Cytosol
Mitochondria
Mr (kDa)
18 mo
12 mo
IB: Calcineurin (α-subunit)
IB: TOMM40
IB: ATP5A
Human patients with dementia likely due to AD (Brain cortex, cytosol)
C
IB: p-S670-GRK2
IB: GRK2
AD pat.
Cont.
IB: PPP2CA
IB: ADSL
D
p-S670-GRK2 (a.u.)
0.0002
GRK2 (a.u.)
0.0715
PPP2CA (a.u.)
0.0003
ADSL (a.u.)
0.3537
AD pat.
Controls
Human patients with dementia likely due to AD (Brain cortex, mitochondria)
E
IB: p-S670-GRK2
AD
Cont
Aggregated p-S670 (a.u.)
<0.0001
Monomeric p-S670 (a.u.)
<0.0001
IB: GRK2
Aggregated GRK2 (a.u.)
0.0002
Monomeric GRK2 (a.u.)
0.0002
F
IB: TOMM6
←M
IB: TOMM40
Aggregated TOMM6 (a.u.)
0.0029
Monom.//Dimer TOMM6 (a.u.)
0.0241
G
AP: p-S670-GRK2
IB: p-S670-GRK2
AP: p-S670-GRK2
IB: TOMM6

Figure S2. Aggregated, mitochondrial phospho-S670-GRK2 in human brain specimens from patients with dementia likely due to AD interacts with TOMM6. Related to Figure 1 and Figure 2.

(A,B) Immunoblot of cytosolic ADSL and Calcineurin (Calcineurin α-subunit), and of mitochondrial TOMM40 and ATP5A (ATP synthase F1 subunit alpha) in hippocampi of 18-month-old (18 mo) and 12-month-old (12 mo) Tg2576 mice. Quantitative immunoblot data (A) and representative immunoblots (B) are shown. Data represent mean ± s.d., n = 4 biological replicates per group. Hippocampi from three male or three female mice were pooled for one biological replicate (6 male and 6 female mice for n = 4 biological replicates). P-values were determined by one-way ANOVA and Tukey's test; $F(3,12) = 118.7$ (ADSL), 155.0 (Calcineurin), 3461 (TOMM40), 2295 (ATP5A).

(C,D) Immunoblot of cytosolic p-S670-GRK2, GRK2, and PPP2CA contents in frontal cortex specimens of human patients with dementia likely due to AD. ADSL was determined as a loading control. Representative immunoblots (C) and quantitative immunoblot data are shown (D). Data are the mean ± s.d. (n = 4 patients with dementia likely due to AD, 3 females, 1 male; n = 4 control patients without dementia, 3 females, 1 male). P-values were determined by the unpaired, two-tailed t-test [(df = 6; t = 8.059 (p-S670), 2.186 (GRK2), 7.453 (PPP2CA), 1.005 (ADSL)].

(E,F) Immunoblot detection of phospho-S670-GRK2 (E, upper) and GRK2 (E, lower), and TOMM6 (F) in mitochondrial protein lysates prepared from human cerebral cortex specimens of four patients with dementia likely due to AD and of four control patients without dementia. Left panels show representative immunoblots, and right panels show quantitative data. TOMM40 was used as a mitochondrial loading control (F). Data represent mean ± s.d. (n = 4 patients with dementia likely due to AD, 3 females, 1 male; n = 4 control patients without dementia, 3 females, 1 male). P-values were determined by the unpaired, two-tailed t-test [df = 6; t = 10.25 (E, aggregated p-S670), 13.76 (E, monomeric p-S670), 8.387 (E, aggregated GRK2), 7.887 (E, monomeric GRK2), 4.850 (F, aggregated TOMM6), 2.999 (F, monomeric-dimeric TOMM6)].

(G) Immunoaffinity-enrichment of phospho-S670-GRK2 from human cerebral cortex specimens from patients with dementia likely due to AD (AP: p-S670-GRK2), and detection of enriched phospho-S670-GRK2 with p-S670-GRK2-specific antibodies (IB: p-S670-GRK2; left panel), and detection of co-enriched TOMM6 by TOMM6-specific antibodies (IB: TOMM6; right panel) in immunoblot. Two patients with dementia likely due to AD (AD) are shown.

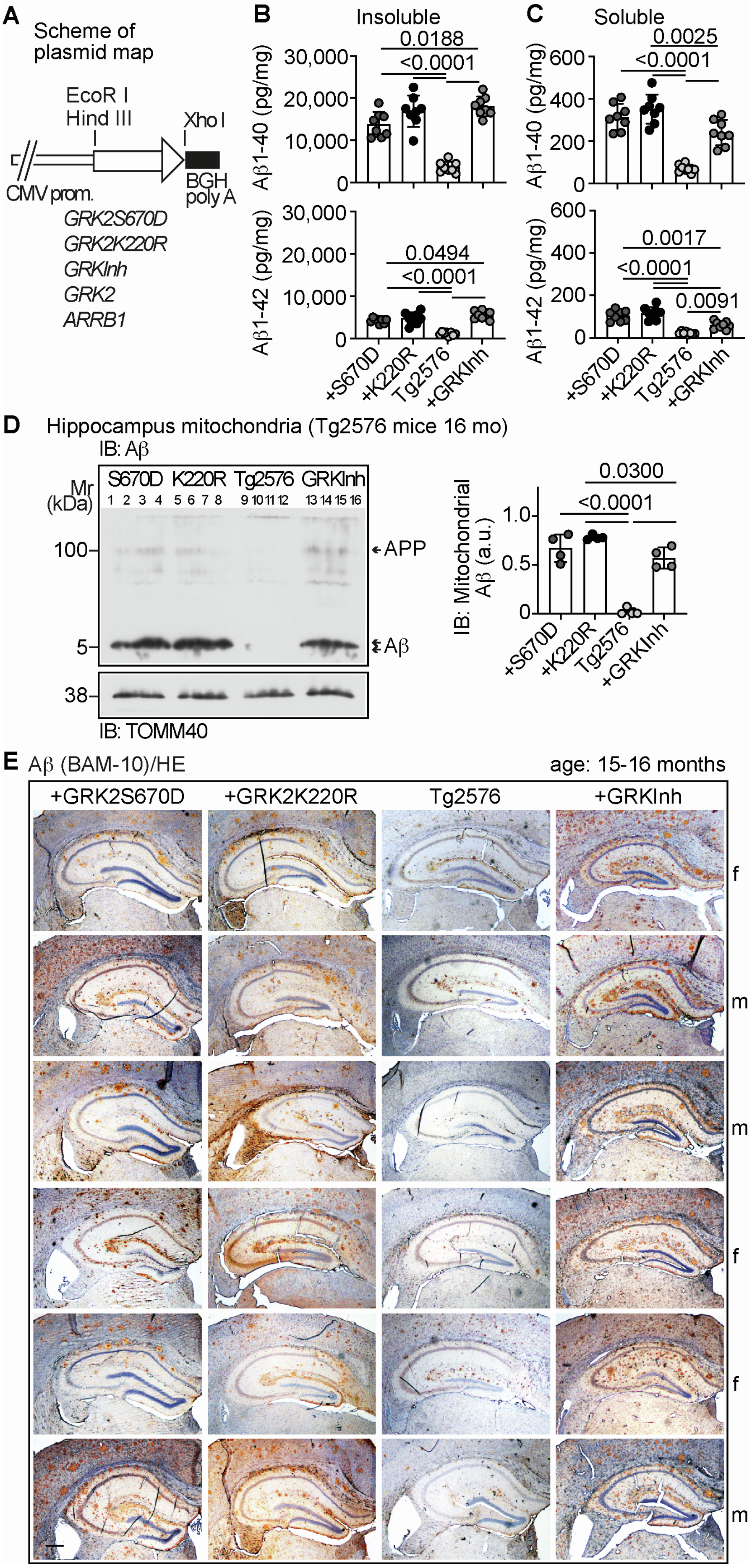
A
Scheme of plasmid map
EcoR I
Hind III
Xho I
CMV prom.
BGH poly A
GRK2S670D
GRK2K220R
GRKInh
GRK2
ARRB1
B
Insoluble
C
Soluble
Aβ1-40 (pg/mg)
Aβ1-42 (pg/mg)
0.0188
<0.0001
0.0494
0.0025
0.0017
0.0091
+S670D
+K220R
Tg2576
+GRKInh
D
Hippocampus mitochondria (Tg2576 mice 16 mo)
IB: Aβ
S670D K220R Tg2576 GRKInh
Mr (kDa)
APP
Aβ
IB: TOMM40
IB: Mitochondrial Aβ (a.u.)
0.0300
E
Aβ (BAM-10)/HE
age: 15-16 months
+GRK2S670D
+GRK2K220R
Tg2576
+GRKInh
f
m
m
f
f
m

Figure S3. GRK2 inactivation by GRK2-S670D, GRK2-K220R and GRKInh leads to increased insoluble, soluble and mitochondrial Aβ, and to Aβ plaque accumulation. Related to Figure 3.

(A) Scheme of plasmid map used for generation of different transgenic mouse lines.

(B,C) Contents of insoluble (B) and soluble (C) Aβ1-40 and Aβ1-42 peptides in brains (hippocampus and frontal cortex) of 16-month-old, double-transgenic Tg2576-*GRK2S670D* mice (+S670D), double-transgenic Tg2576-*GRK2K220R* mice (+K220R), single-transgenic Tg2576 mice (Tg2576), and double-transgenic Tg2576-*GRKInh* mice (+GRKInh). Data represent mean ± s.d., n=8 mice per group (4 females and 4 males). P-values were determined by one-way ANOVA and Tukey's test; $F(3,28) = 46.13$, 34.80 (B); 39.62, 31.54 (C).

(D) Immunoblot detection of mitochondrial Aβ in hippocampal, mitochondrial protein lysates of 16-month-old, Tg2576-*GRK2S670D* mice (+S670D), Tg2576-*GRK2K220R* mice (+K220R), Tg2576 mice (Tg2576), and Tg2576-*GRKInh* mice (+GRKInh). The blotting time was reduced to 30 min to detect preferentially monomeric Aβ peptides. The left panel shows a representative immunoblot, and the right panel shows quantitative immunoblot data of monomeric mitochondrial Aβ peptides. Arrowheads mark monomeric Aβ peptides and APP. TOMM40 was used as a mitochondrial loading control. Data represent mean ± s.d; n=4 biological replicates per group. Hippocampi from three male or three female mice were pooled for one biological replicate (6 male and 6 female mice for n = 4 biological replicates). P-values were determined by one-way ANOVA and Tukey's test; $F(3,12)=54.67$.

(E) Immunohistological detection of hippocampal Aβ plaques (brown) on coronal brain sections of 15-16-month-old double-transgenic Tg2576-*GRK2S670D* mice (+GRK2S670D), double-transgenic Tg2576-*GRK2K220R* mice (+GRK2K220R), single-transgenic Tg2576 mice (Tg2576), and double-transgenic Tg2576-*GRKInh* mice (+GRKInh) was performed by immunostaining with Aβ-specific antibody, BAM-10. Counterstaining was performed with hematoxylin (HE). Immunohistological images are from six mice per group with 3 females (f), and 3 males (m); bar: 200 µm. Quantitative data showing the area covered by Aβ plaques are shown in Figure 3L, and two representative immunohistological sections per group are presented in Figure 3M.

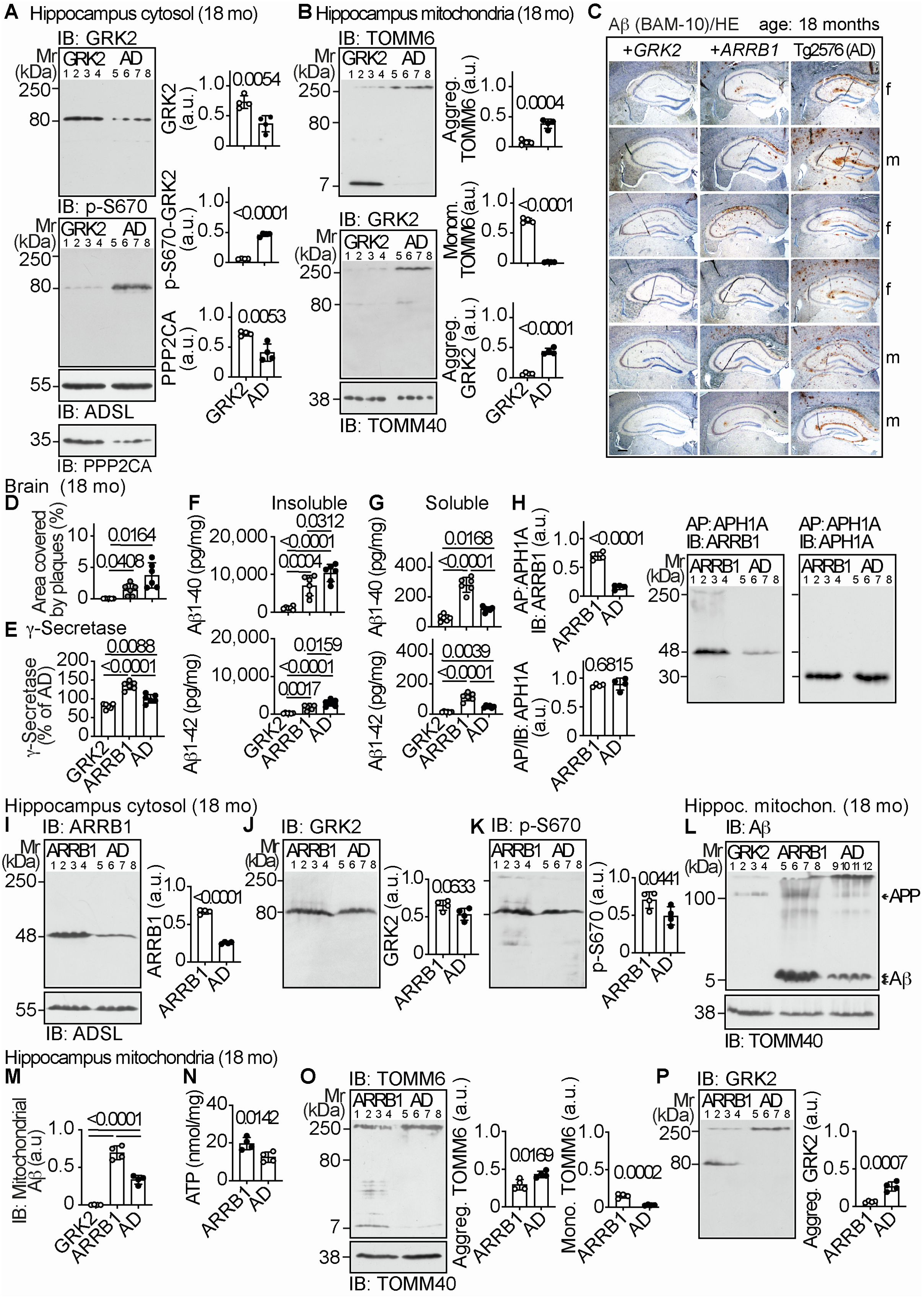

Figure S4. Neuroprotective GRK2 activities differ from ARRB1-mediated effects. Related to Figure 3.

(A) Immunoblot analysis of hippocampal cytosolic GRK2, p-S670-GRK2, and PPP2CA levels in 18-month-old, double-transgenic Tg2576-*GRK2* mice (GRK2) and in single-transgenic Tg2576 AD mice (A). The control

immunoblot detects cytosolic ADSL. Left panels show representative immunoblots, and right panels show quantitative data (mean ± s.d., n = 4 mice/group; 2 females, 2 males). P-values were determined with the unpaired, two-tailed t-test (df = 6; t = 4.243, 26.19, 4.271).

(B) Immunoblot quantification of hippocampal mitochondrial, aggregated and monomeric TOMM6 levels, and aggregated GRK2 levels in 18-month-old, double-transgenic Tg2576-*GRK2* mice (GRK2) and in single-transgenic Tg2576 AD mice (AD). The control immunoblot detects mitochondrial TOMM40. Left panels show representative immunoblots, and right panels show quantitative data (mean ± s.d., n = 4 biological replicates per group). Hippocampi from three male or three female mice were pooled for one biological replicate (6 male and 6 female mice for n = 4 biological replicates). P-values were determined with the unpaired, two-tailed t-test (df = 6; t = 7.122, 35.58, 13.52).

(C) Immunohistological detection of hippocampal Aβ plaques (brown) on coronal brain sections of 18-month-old, double-transgenic Tg2576-*GRK2* mice (+*GRK2*), double-transgenic Tg2576-*ARRB1* mice (+*ARRB1*), and single-transgenic Tg2576 AD mice was performed by immunostaining with β-amyloid-specific antibody, BAM-10. Counterstaining was performed with hematoxylin (HE). Immunohistological images are from n = 6 mice per group with 3 females (f) and 3 males (m); bar: 200 µm. Two representative immunohistological sections of double-transgenic Tg2576-*GRK2* mice and single-transgenic Tg2576 AD mice are shown in Figure 3M.

(D) Quantitative analysis of immunohistological sections shown in (C) determined the area (%) covered by Aβ plaques. Data represent mean ± s.d., n = 6 mice per group (3 females, 3 males). Quantitative data of double-transgenic Tg2576-*GRK2* mice and single-transgenic Tg2576 AD mice presenting the area covered by Aβ plaques are also shown in Figure 3L. P-values were determined by Welch's ANOVA test followed by Dunnett's T3 test; W(2,6.668) = 15.23.

(E) Activity of hippocampal γ-secretase in 18-month-old, double-transgenic Tg2576-*GRK2* mice (GRK2), double-transgenic Tg2576-*ARRB1* mice (ARRB1), and single-transgenic Tg2576 AD mice (AD). Data represent means ± s.d., n = 6 mice per group (3 females, 3 males). P-values were determined by one-way ANOVA followed by Tukey's test; F(2,15) = 47.39.

(F,G) Contents of insoluble (F) and soluble (G) Aβ1-40 and Aβ1-42 peptides in brains (hippocampus and frontal cortex) of 18-month-old, double-transgenic Tg2576-*GRK2* mice (GRK2), double-transgenic Tg2576-*ARRB1* mice (ARRB1), and single-transgenic Tg2576 AD mice (AD). Data are mean ± s.d., n=6 mice per group (3 females, 3 males). P-values were determined by one-way ANOVA followed by Tukey's test; F(2,15) = 31.84 (F), 28.19 (F), 74.31 (G), 62.14 (G).

(H) Immunoaffinity enrichment of APH1A (AP: APH1A) with an anti-APH1A antibody-containing affinity matrix and immunoblot detection of co-enriched ARRB1 (IB: ARRB1) was performed with brain lysates (frontal cortex, hippocampus) from 18-month-old, double-transgenic Tg2576-*ARRB1* (ARRB1) mice and single-transgenic Tg2576 (AD) mice. The bar graphs show quantitative immunoblot data (left panels), and the middle and right panels show representative immunoblots of ARRB1 co-enriched with the anti-APH1A affinity matrix (AP:APH1A, IB:ARRB1) and of APH1A(-L), which was enriched with the anti-APH1A affinity matrix (AP:APH1A, IB:APH1A). Data are presented as mean ± s.d., n = 4 biological replicates. Brains from three male or three female mice were pooled for one biological replicate (6 male mice and 6 female mice for n = 4 biological replicates). P-values were determined with the unpaired, two-tailed t-test (df = 6; t =13.75, 0.4310).

(I,J,K) Immunoblot detection of hippocampal, cytosolic ARRB1 (I), GRK2 (J), and phospho-S670-GRK2 (K) in 18-month-old Tg2576-*ARRB1* mice (ARRB1) and in age-matched Tg2576 AD mice (AD). The lower control blot (I) detects ADSL. Bar graphs show quantitative immunoblot data (mean ± s.d., n = 4 mice per group; 2 females and 2 males). P-values were determined by the unpaired, two-tailed t-test [df = 6; t = 25.50 (I), 2.274 (J), 2.539 (K)].

(L,M) Immunoblot determination of mitochondrial Aβ in hippocampal mitochondria from 18-month-old, double-transgenic Tg2576-*GRK2* mice (GRK2), double-transgenic Tg2576-*ARRB1* mice (ARRB1), and single-transgenic Tg2576 AD mice (AD). The blotting time was reduced to 30 min to detect preferentially monomeric Aβ peptides. Representative immunoblots (L) are presented, and the bar graph (M) shows quantitative data. The lower control blot (L) detects mitochondrial TOMM40. Data are presented as mean ± s.d., n = 4 biological replicates per group. Hippocampi from three male mice or three female mice were pooled for one biological replicate (6 male mice and 6 female mice for n = 4 biological replicates). P-values were determined by one-way ANOVA followed by Tukey's test; F(2,9) = 126.9.

(N) Hippocampal, mitochondrial ATP levels of 18-month-old Tg2576-*ARRB1* (ARRB1) and Tg2576 AD mice (AD). Data are presented as mean ± s.d., with n = 4 mice per group (2 females and 2 males). P-values were determined by the unpaired, two-tailed t-test; df = 6; t = 3.418.

(O,P) Quantitative immunoblot analysis of hippocampal mitochondrial TOMM6 (O), and GRK2 (P) in 18-month-old Tg2576-*ARRB1* (ARRB1) and Tg2576 AD mice (AD). The lower control immunoblot (O) detects TOMM40. Data are mean ± s.d., with n = 4 biological replicates per group. Hippocampi from three male mice or three female mice were pooled for one biological replicate (6 male mice and 6 female mice for n = 4 biological replicates). P-values were determined by the unpaired, two-tailed t-test; df = 6; t = 3.279 (O), 8.131 (O), 6.431 (P).

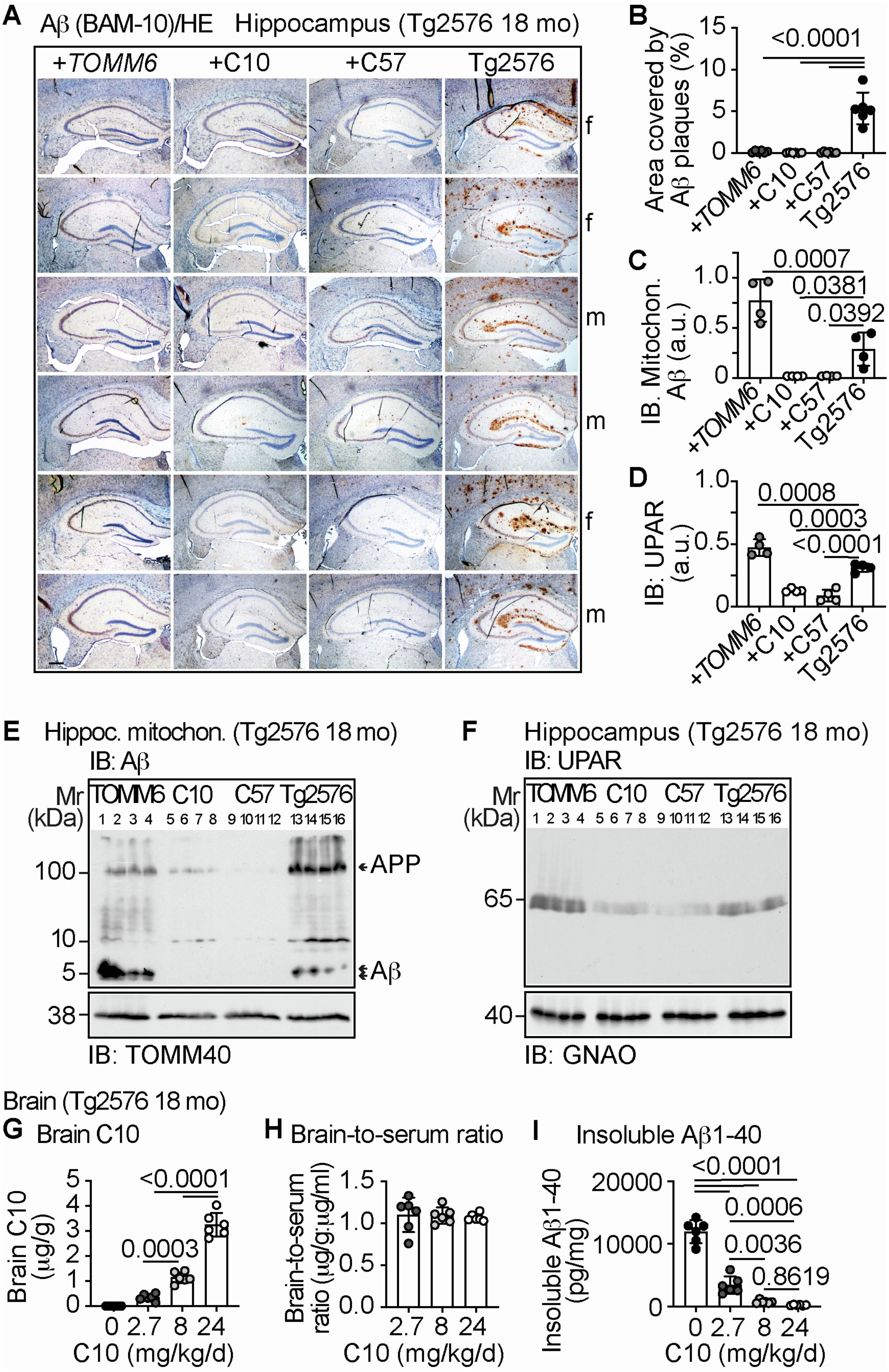

Figure S5. Effects of neuron-specific *TOMM6* expression, and CPD10 or CPD57 treatment on Aβ plaque accumulation, mitochondrial Aβ and the senescence marker UPAR (urokinase plasminogen activator receptor). Related to Figures 4, 6, and 7.

(A) Immunohistological detection of hippocampal Aβ plaques (brown) on coronal brain sections of 18-month-old, untreated Tg2576-*TOMM6* mice (+*TOMM6*), 18-month-old Tg2576 mice treated for 6 months with CPD10 (+C10; 8 mg/kg/d), or with CPD57 (+C57; 10 mg/kg/d), and of 18-month-old, untreated Tg2576 mice (Tg2576). Specimens are from n = 6 mice per group, with three female (f), and three male (m) mice; bar: 200 μm. Figure 4J shows two representative immunohistological sections of Tg2576-*TOMM6* and Tg2576 mice. Figure 6M shows four representative sections of CPD10-treated and untreated Tg2576 mice. Figure 7L shows two representative sections of CPD57-treated and untreated Tg2576 mice.
(B) The area covered by Aβ plaques (%) was determined by quantitative image analysis of immunohistological sections shown in (A). Data are mean ± s.d., n = 6 mice/group. The P-value was determined by one-way ANOVA and Tukey's test, $F(3,20) = 45.20$.
(C,E) Immunoblot detection of mitochondrial Aβ (IB: Aβ) in hippocampal mitochondria of 18-month-old, untreated Tg2576-*TOMM6* mice (+*TOMM6*), 18-month-old Tg2576 mice treated for 6 months with CPD10 (+C10), or with CPD57 (+C57), and of 18-month-old, untreated Tg2576 mice (Tg2576). Panel (C) shows quantitative immunoblot data, and (E) shows representative immunoblots. Data represent mean ± s.d., n = 4 biological replicates per group. Hippocampi from three male or three female mice were pooled for one biological replicate (6 male mice and 6 female mice for n = 4 biological replicates). The lower control blot (E) detects TOMM40. Arrowheads mark Aβ peptides, and APP. P-values were determined by one-way ANOVA and Dunnett's test, $F(3,12) = 27.90$ (C).
(D,F) Immunoblot detection of hippocampal contents of the senescence marker, UPAR, in 18-month-old, untreated Tg2576-*TOMM6* mice (+*TOMM6*), 18-month-old Tg2576 mice treated for 6 months with CPD10 (+C10), or with CPD57 (+C57), and in 18-month-old, untreated Tg2576 mice (Tg2576). Panel (D) shows quantitative immunoblot data and (F) shows representative immunoblots. Data are mean ± s.d., n = 4 mice (2 female, 2 male) per group. The control blot detects GNAO. P-values were determined by one-way ANOVA and Dunnett's test, $F(3,12) = 61.54$.
(G,H) Brain concentrations (G) and brain-to-serum ratios (H) of CPD10 (C10) in 18-month-old Tg2576 mice treated for 6 months with the indicated doses of CPD10. Data are presented as mean ± s.d., n = 6 mice (3 female and 3 male) per group. P-values were determined by one-way ANOVA and Tukey's test, $F(3,20) = 176.1$ (G); $F(2,15) = 0.09057$ (H).
(I) Insoluble Aβ1-40 contents in brains (frontal cortex and hippocampus) of 18-month-old Tg2576 mice treated for 6 months with the indicated dose of CPD10. Data are presented as mean ± s.d., n = 6 mice (3 female, 3 male) per group. The P-value was determined by one-way ANOVA and Tukey's test, $F(3,20) = 132.7$.

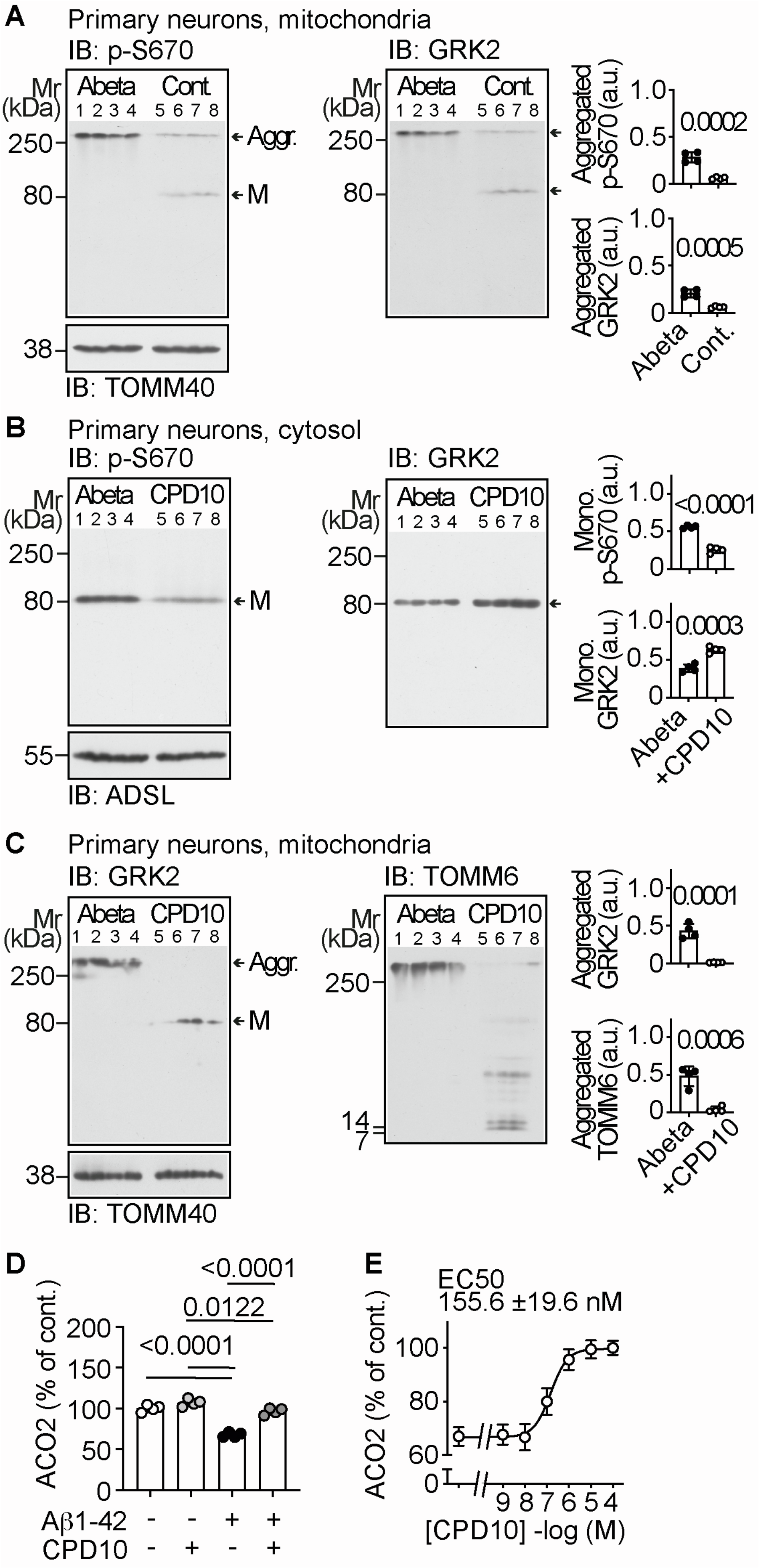

A
Primary neurons, mitochondria
IB: p-S670
IB: GRK2
Mr (kDa)
Abeta
Cont.
1 2 3 4 5 6 7 8
250
80
38
Aggr.
M
IB: TOMM40
Aggregated p-S670 (a.u.)
0.0002
Aggregated GRK2 (a.u.)
0.0005
Abeta
Cont.
B
Primary neurons, cytosol
IB: p-S670
IB: GRK2
CPD10
55
IB: ADSL
Mono. p-S670 (a.u.)
<0.0001
Mono. GRK2 (a.u.)
0.0003
+CPD10
C
Primary neurons, mitochondria
IB: GRK2
IB: TOMM6
14
7
Aggregated GRK2 (a.u.)
0.0001
Aggregated TOMM6 (a.u.)
0.0006
D
ACO2 (% of cont.)
<0.0001
0.0122
<0.0001
Aβ1-42
CPD10
E
EC50
155.6 ±19.6 nM
[CPD10] -log (M)

Figure S6. CPD10 effects on primary mouse cortical neurons. Related to Figure 5.

(A) Immunoblot detection of mitochondrial phospho-S670-GRK2 (IB: p-S670) and GRK2 of primary mouse cortical neurons treated for 48 h without (Cont.) or with Aβ1-42 (Abeta; 20 μM of aggregated Aβ1-42). Left and middle panels show representative immunoblots. The lower control immunoblot detects the mitochondrial marker TOMM40. Right panels represent quantitative data as mean ± s.d. (n = 4 biological replicates/group). P-values were determined with the unpaired, two-tailed t-test (df = 6, t = 7.899, 6.835).

(B) Immunoblot analysis (IB:) of phospho-S670-GRK2 and GRK2 in cytosolic protein fractions of primary mouse cortical neurons treated for 48 h with Aβ1-42 (Abeta; 20 μM of aggregated Aβ1-42), or with aggregated Aβ1-42 and CPD10 (+CPD10, 10 μM). Left and middle panels show representative immunoblots, and right panels show quantitative data (mean ± s.d., n = 4 biological replicates/group). P-values were determined with the unpaired, two-tailed t-test (df = 6, t = 13.68, 7.493). The lower control immunoblot detects ADSL.

(C) Immunoblot detection of GRK2 and TOMM6 in mitochondrial protein fractions of primary mouse cortical neurons treated for 48 h with aggregated Aβ1-42 (Abeta), or with aggregated Aβ1-42 and CPD10 (+CPD10, 10 μM). Left and middle panels show representative immunoblots and right panels show quantitative data (mean ± s.d., n=4 biological replicates/group). P-values were determined with the unpaired, two-tailed t-test (df = 6, t = 8.483, 6.588). The lower control immunoblot detects TOMM40.

(D) Mitochondrial ACO2 activity of primary mouse cortical neurons treated for 48 h without (control) or with aggregated Aβ1-42, or with aggregated Aβ1-42 and CPD10 (+CPD10, 10 μM). Data are expressed as % of control (set to 100 %) and represent the mean ± s.d., n=4 biological replicates/group. P-values were determined by one-way ANOVA followed by Tukey's test; $F(3,12) = 74.82$.

(E) Mitochondrial ACO2 activity was determined of primary mouse cortical neurons treated for 48 h without (control) or with aggregated Aβ1-42 and increasing concentrations of CPD10 as indicated. Data are presented as % of control (set to 100 %) and show a representative experiment performed in triplicates (mean ± s.d.). The EC50 value of CPD10 was determined by four parameter logistic (4PL) curve fit of three independent experiments (performed with three technical replicates each) and is expressed as mean ± s.d.

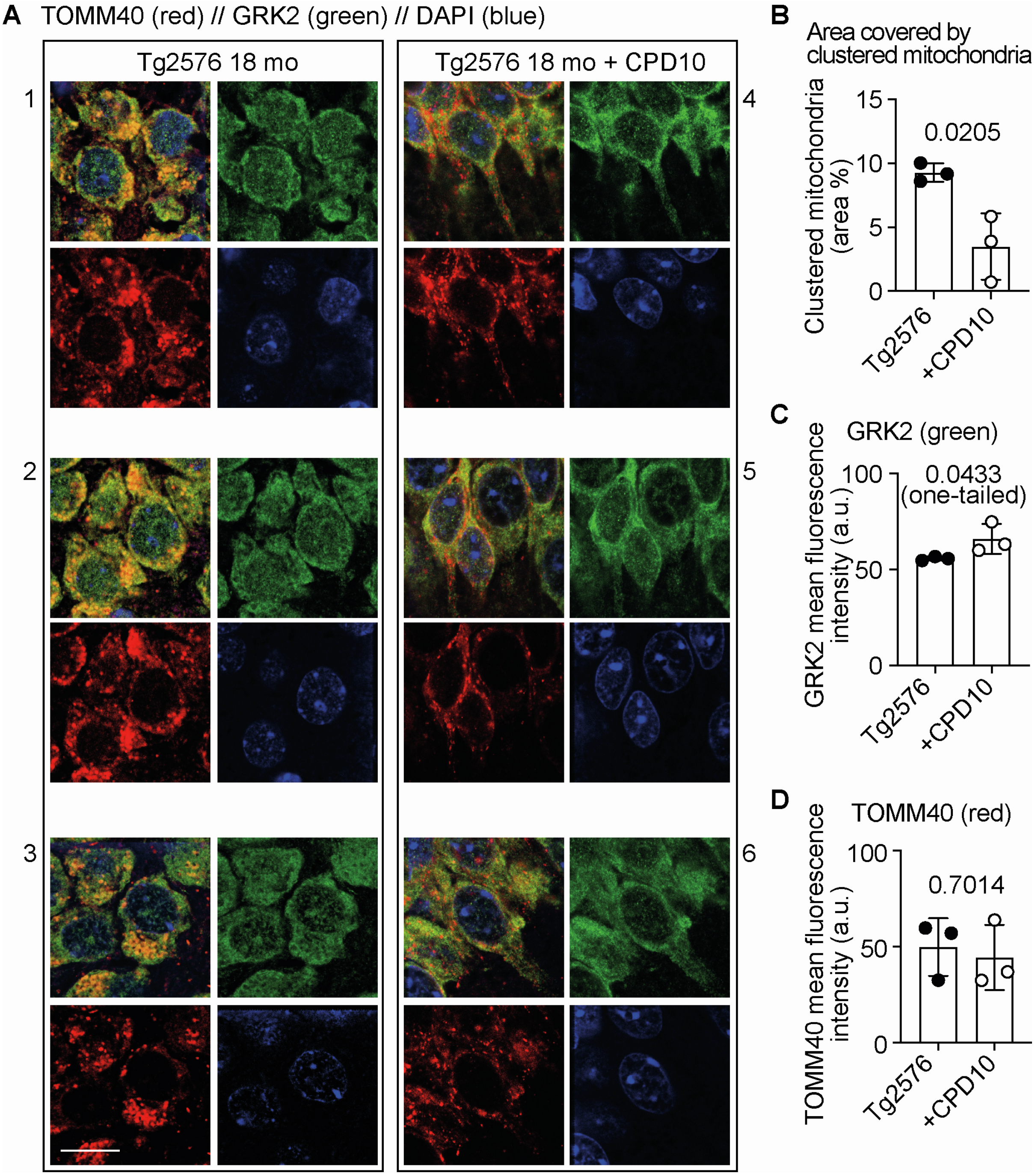

Figure S7. Treatment with the GRK2 function modulator, CPD10, prevents mitochondrial clustering in brains of Tg2576 mice. Related to Figure 6.

(A) Immunofluorescence imaging of mitochondrial TOMM40 (red) and GRK2 (green) in the hippocampal CA1 area of brain specimens from untreated, 18-month-old Tg2576 mice (Tg2576 18 mo), and from age-matched Tg2576 mice treated with CPD10 (8 mg/kg/d) for 6 months (Tg2576 18 mo + CPD10). Nuclei were stained with DAPI (blue). Brain sections are from three different untreated Tg2576 mice (1-3) and from three different CPD10-treated mice (4-6); bar: 20 µm. Sections are from female mice (1,2,4,5), and male mice (3,6). Representative immunofluorescence images of a CPD10-treated Tg2576 mouse (mouse 4) and an untreated Tg2576 mouse (mouse 1) are shown in Figure 6I.

(B-D) Quantitative evaluation of immunofluorescence images shown in (A) determined the area covered by clustered mitochondria (B), the GRK2 mean fluorescence intensity (C), and the TOMM40 mean fluorescence intensity (D). Data are presented as mean ± s.d., n=3 mice per group. P-values were determined by the unpaired, two-tailed (B,D) or one-tailed (C) t-test; df = 4; t = 3.721 (B); 2.263 (C); 0.4120 (D).

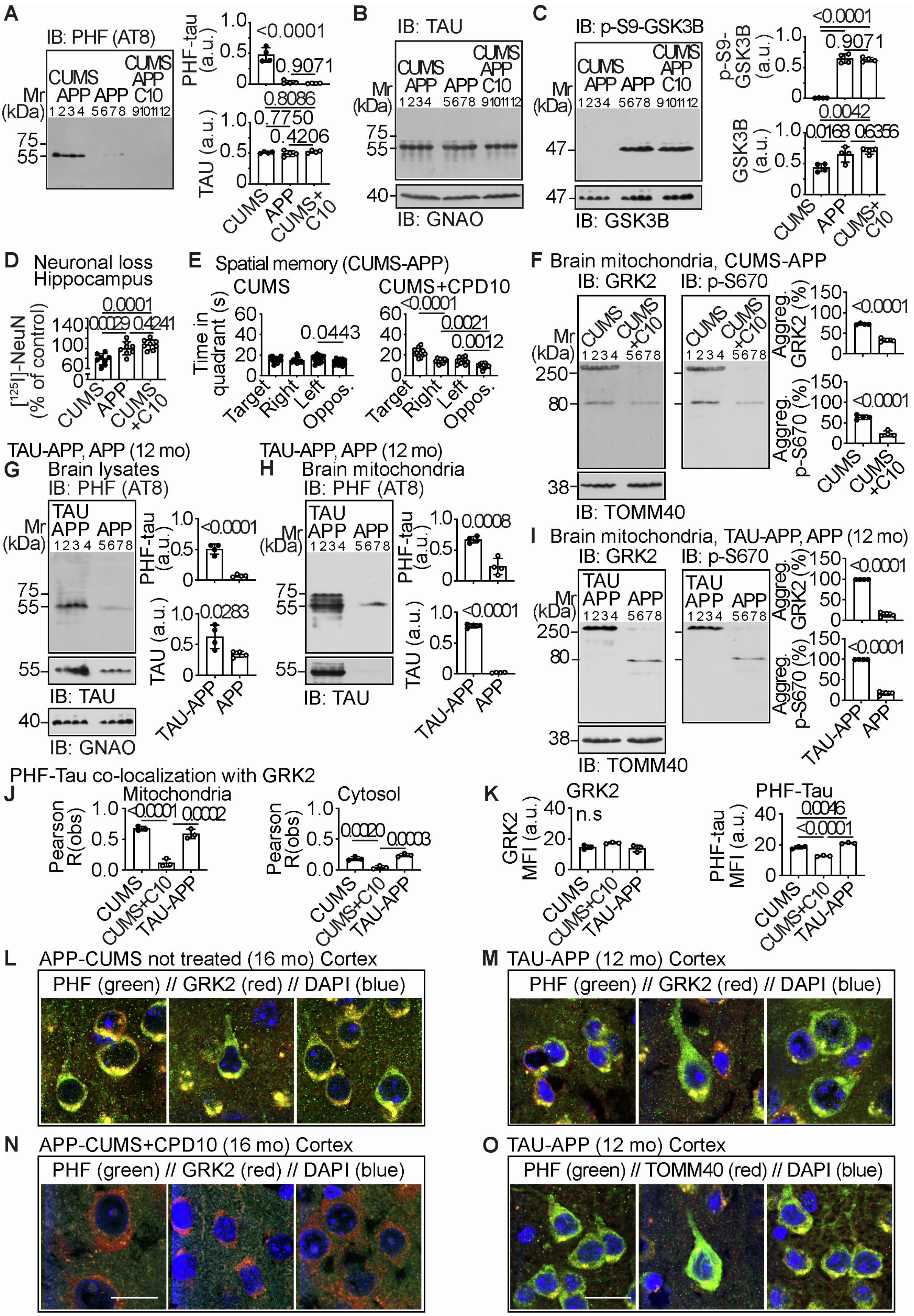
A
IB: PHF (AT8)
CUMS APP APP CUMS APP C10
Mr (kDa)
75
55
PHF-tau (a.u.)
<0.0001
0.9071
0.8086
0.7750
0.4206
TAU (a.u.)
CUMS
APP
CUMS+ C10
B
IB: TAU
IB: GNAO
C
IB: p-S9-GSK3B
IB: GSK3B
p-S9-GSK3B (a.u.)
<0.0001
0.9071
0.0042
0.0168
0.6356
GSK3B (a.u.)
D Neuronal loss Hippocampus
[125I]-NeuN (% of control)
0.0001
0.0029
0.4241
E Spatial memory (CUMS-APP)
CUMS
CUMS+CPD10
Time in quadrant (s)
0.0443
<0.0001
0.0021
0.0012
Target
Right
Left
Oppos.
F Brain mitochondria, CUMS-APP
IB: GRK2
IB: p-S670
CUMS
CUMS +C10
Aggreg. GRK2 (%)
Aggreg. p-S670 (%)
<0.0001
IB: TOMM40
TAU-APP, APP (12 mo)
G Brain lysates
IB: PHF (AT8)
TAU APP APP
<0.0001
0.0283
IB: TAU
IB: GNAO
TAU-APP
APP
H Brain mitochondria
IB: PHF (AT8)
0.0008
<0.0001
I Brain mitochondria, TAU-APP, APP (12 mo)
IB: GRK2
IB: p-S670
PHF-Tau co-localization with GRK2
J Mitochondria
Cytosol
Pearson R(obs)
<0.0001
0.0002
0.0020
0.0003
CUMS
CUMS+C10
TAU-APP
K GRK2
n.s
GRK2 MFI (a.u.)
PHF-Tau
PHF-tau MFI (a.u.)
0.0046
<0.0001
L APP-CUMS not treated (16 mo) Cortex
PHF (green) // GRK2 (red) // DAPI (blue)
M TAU-APP (12 mo) Cortex
PHF (green) // GRK2 (red) // DAPI (blue)
N APP-CUMS+CPD10 (16 mo) Cortex
PHF (green) // GRK2 (red) // DAPI (blue)
O TAU-APP (12 mo) Cortex
PHF (green) // TOMM40 (red) // DAPI (blue)

Figure S8. GRK2 function modulation by CPD10 retards neurodegenerative PHF-tau hyperphosphorylation and neuronal loss. Related to Figure 6.

(A,B) Hippocampal contents of hyperphosphorylated PHF-tau were determined by immunoblot (IB) with AT8 antibody (A), and contents of total TAU were determined with anti-TAU antibody (B) of hippocampal lysates of 16-month-old, CUMS-subjected male Tg2576 mice [(CUMS APP (immunoblots) and CUMS (bar graph)], 16-month-old male Tg2576 mice (APP), and 16-month-old, CUMS-subjected male Tg2576 mice with CPD10 treatment (8 mg/kg/d) during the CUMS protocol [(CUMS APP C10 (immunoblots) and CUMS+C10 (bar graph)]. Representative immunoblots (A, left panel; B) and quantitative immunoblot data (A, right panels) are shown. The lower control blot detects GNAO (B). Data are presented as mean ± s.d., n = 4 male mice/group. P-values were determined by one-way ANOVA followed by Tukey's test; $F(2,9) = 57.96$, 0.8698.

(C) Hippocampal contents of inactive, serine-9-phosphorylated GSK3B (p-S9-GSK3B) were determined in 16-month-old, CUMS-subjected, male Tg2576 mice [(CUMS APP (immunoblot) and CUMS (bar graph)], 16-month-old male Tg2576 mice (APP), and 16-month-old, CUMS-subjected male Tg2576 mice with CPD10 treatment during the CUMS protocol [(CUMS APP C10 (immunoblot) and CUMS+C10 (bar graph)]. The lower blot shows total hippocampal GSK3B levels. The right panels show quantitative immunoblot data. Data represent mean ± s.d., n = 4 male mice/group. P-values were determined by one-way ANOVA followed by Tukey's test; $F(2,9) = 231.8$ and 10.90.

(D) Hippocampal neuronal loss was determined by a radioactive binding assay, which quantified hippocampal neuronal cell bodies with [^{125}I]-labeled NeuN antibody in 16-month-old, male Tg2576 mice subjected to CUMS (CUMS), 16-month-old, male Tg2576 mice without CUMS (APP; control, set to 100 %), and 16-month-old, male, CUMS-subjected Tg2576 mice with CPD10 treatment during the CUMS protocol (CUMS+C10). Data are mean ± s.d., n = 8 male mice/group. P-values were determined by one-way ANOVA followed by Tukey's test; $F(2,21) = 13.90$.

(E) CPD10 treatment improved the spatial memory of CUMS-subjected, male Tg2576 mice (CUMS+CPD10; right panel) compared to untreated CUMS-subjected, male Tg2576 mice (CUMS; left panel). Data are mean ± s.d., n = 12 male mice/group. P-values were determined by one-way ANOVA followed by Tukey's test; $F(3,44) = 2.629$ (untreated; CUMS), 37.81 (treated; CUMS + CPD10).

(F) Immunoblot (IB) of mitochondrial GRK2 and p-S670-GRK2 aggregates (shown as % of total GRK2 or p-S670-GRK2) in brains of 16-month-old, CUMS-subjected male Tg2576 mice without treatment (CUMS) and with CPD10 treatment (CUMS+C10). The left and middle panels show representative immunoblots, and the right panels show quantitative data (mean ± s.d., n=4 biological replicates per group). Brains from three male mice were pooled for one biological replicate (12 male mice for n = 4 biological replicates). P-values were determined by the unpaired, two-tailed t-test; df = 6; t = 16.52, 9.353.

(G,H) Immunoblot (IB) of PHF-tau in brain (hippocampus, frontal cortex) lysates (G) and brain mitochondria (H) of 12-month-old, male, double-transgenic Tg2576-TAU-P301L (TAU-APP) mice and Tg2576 (APP) mice. The middle (G) and lower (H) panels detect total TAU, and the lower panel (G) is a loading control detecting GNAO. The left panels show representative immunoblots, and the right panels show quantitative immunoblot data [mean ± s.d., n = 4 mice/group (G), and n = 4 biological replicates (H)]. Brains from three male mice were pooled for one biological replicate (12 male mice for n = 4 biological replicates). P-values were determined by the unpaired, two-tailed t-test; df = 6; t = 10.37, 2.875 (G); 6.196, 47.92 (H).

(I) Immunoblot (IB) of aggregated mitochondrial GRK2 and p-S670-GRK (shown as % of total) in brains (hippocampus, frontal cortex) of 12-month-old, male, double-transgenic Tg2576-TAU-P301L (TAU-APP) and single-transgenic Tg2576 (APP) mice. The lower left panel detects TOMM40. Data are mean ± s.d., n=4 biological replicates per group. Brains from three male mice were pooled for one biological replicate (12 male mice for n = 4 biological replicates). P-values were determined by the unpaired, two-tailed t-test; df = 6; t = 31.20, 35.55.
(J) Observed Pearson correlation coefficients [Pearson R(obs)] for the mitochondrial (left) and cytosolic (right) co-localization of PHF-tau with GRK2 on frontal cortex specimens of 16-month-old, male, CUMS-subjected Tg2576 mice without treatment (CUMS), 16-month-old, male, CUMS-subjected Tg2576 mice with CPD10 treatment (CUMS+C10), and of 12-month-old, male Tg2576-TAU-P301L mice without treatment (TAU-APP).
(K) Quantitative immunofluorescence data show the mean fluorescence intensity (MFI) of GRK2 (K, left) was not significantly different whereas the MFI of PHF-tau was elevated (K, right) in brains of CUMS-subjected Tg2576 (CUMS) and Tg2576-TAU-P301L (TAU-APP) mice compared to CPD10-treated, CUMS-subjected Tg2576 mice (CUMS+C10).
(J,K) Data represent mean ± s.d., of n=3 male mice per group. P-values were determined by one-way ANOVA followed by Tukey's test; F(2,6) = 71.75, 39.78 (J); 4.593, 134.6 (K).
(L-O) Immunofluorescence imaging [(quantitative data shown in (J,K)] of PHF-tau (green) and GRK2 (L-N, red) or the mitochondrial marker TOMM40 (O, red), on frontal cortex specimens of 16-month-old, CUMS-subjected Tg2576 (APP-CUMS not treated) mice (L), 12-month-old Tg2576-TAU-P301L (TAU-APP) mice (M,O), and 16-month-old, CPD10-treated (8 mg/kg/d, for two months during the CUMS protocol), CUMS-subjected Tg2576 (APP-CUMS+CPD10) mice (N). Nuclei were stained with DAPI (blue). Images are from n=3 male mice per group; bar: 20 μm.

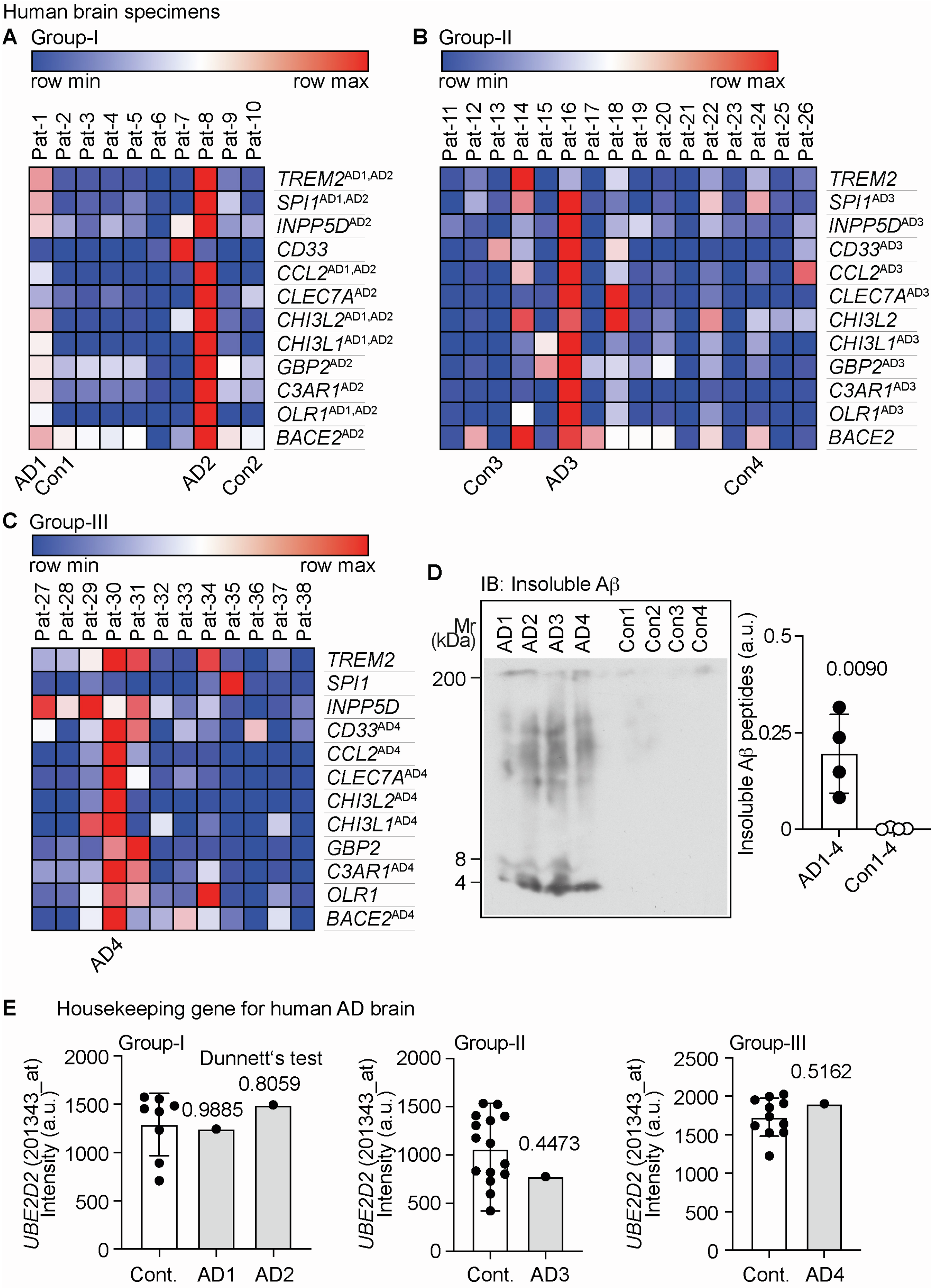

Figure S9. Whole genome microarray transcriptome profiling of AD patient brain biopsy specimens. Related to STAR Methods and Key Resources Table.

(A-C) Expression levels of AD-related transcripts were determined by whole genome microarray gene expression profiling in brain cortex specimens of human patients with dementia likely due to AD (AD1, AD2,

AD3, AD4) in comparison to control patients. The following AD-related transcripts with corresponding Affymetrix probe set IDs are shown (*TREM2*: 219725_at; *SPI1*: 205312_at; *INPP5D*: 203332_s_at; *CD33*: 206120_at; *CCL2*: 216598_s_at; *CLEC7A*: 221698_s_at; *CHI3L2*: 213060_s_at; *CHI3L1*: 209395_at; *GBP2*: 202748_at; *C3AR1*: 209906_at; *OLR1*: 210004_at; *BACE2*: 217867_x_at). Probe set intensities are presented as heat maps. Brain biopsy specimens were taken from three groups of glioblastoma patients (Group-I, Group-II, Group-III) in frame of surgery for glioblastoma. AD-related probe sets of transcripts with significantly increased signal intensities in AD patients (AD1, AD2, AD3, AD4) in comparison to those of patients without dementia are marked in uppercase letters after the gene name. Significant differences between probe set intensities of patients with dementia likely due to AD (AD1, AD2, AD3, AD4) and patients without dementia were determined within the individual groups by one-way ANOVA and Dunnett's test. Age- and gender-matched control patients (Con1 - Con4) which served as controls for protein studies, are also marked.

(D) Immunoblot detection of insoluble Aβ (IB: insoluble Aβ) contents in formic acid extracts of brain specimens from four patients with dementia likely due to AD (AD1-AD4) in comparison to those from four age- and gender-matched control patients without dementia (Con1-Con4). The right panel shows quantitative immunoblot data of insoluble Aβ peptides. Data are presented as mean ± s.d., $n = 4$. The p-value was determined by the unpaired, two-tailed t-test ($df = 6$, $t = 3.794$).

(E) Expression levels of the housekeeping gene for human AD brain (*UBE2D2*) were not significantly different between human brain specimens from patients with dementia likely due to AD and specimens of patients without dementia (Cont.). P-values were determined by one-way ANOVA and Dunnett's test (left panel), and the unpaired, two-tailed t-test (middle and left panels); $F(2,7) = 0.1953$; $df = 14$, $t = 0.7819$; $df = 10$, $t = 0.6731$. Data are mean ± s.d., $n = 8$ (left panel), $n = 15$ (middle panel), $n = 11$ (right panel).

Assay<br>(Radioligand competition assay; h: human)	1st replicate / % of Control Specific Binding	2nd replicate / % of Control Specific Binding	Mean / % of Control Specific Binding
ADORA2A (h) (agonist radioligand)	86.5	95.5	91
ADRA1A (h) (antagonist radioligand)	100.4	99.3	99.9
ADRA2A (h) (antagonist radioligand)	99.4	100	99.7
ADRB1 (h) (agonist radioligand)	90.2	90.8	90.5
ADRB2 (h) (antagonist radioligand)	94.6	101	97.8
BZD (central) ([^{3}H]flunitrazepame binding site; rat cerebral cortex; agonist radioligand)	103	93.4	98.2
CNR2 (h) (agonist radioligand)	100.1	96.9	98.5
CNR1 (h) (agonist radioligand)	97.4	100.7	99
CCKAR (CCK1) (h) (agonist radioligand)	117.5	111.3	114.4
DRD1 (h) (antagonist radioligand)	97.6	93.8	95.7
DRD2 short isoform (h) (agonist radioligand)	113.2	108.1	110.7
EDNRA (h) (agonist radioligand)	65	64	64.5
NMDA ([^{3}H]CGP 39653, rat cerebral cortex; antagonist radioligand)	103.1	104.1	103.6
HRH1 (h) (antagonist radioligand)	98.6	100	99.3
HRH2 (h) (antagonist radioligand)	90.3	90	90.2
MAOA ([^{3}H]Ro 41-1049 binding site, rat cerebral cortex; antagonist radioligand)	115	109.9	112.5
CHRM1 (h) (antagonist radioligand)	100.2	86.1	93.1
CHRM2 (h) (antagonist radioligand)	90.3	102.6	96.5
CHRM3 (h) (antagonist radioligand)	101	92	96.5
N neuronal alpha4beta2 (h) (agonist radioligand)	98.6	100.2	99.4
OPRD1 (h) (agonist radioligand)	95	95.7	95.3
OPRK1 (h) (agonist radioligand)	96.2	98.7	97.5
OPRM1 (h) (agonist radioligand)	90.8	89.4	90.1
HTR1A (h) (agonist radioligand)	112.7	82.3	97.5
HTR1B (h) (antagonist radioligand)	92.1	94.2	93.2
HTR2A (h) (agonist radioligand)	97.7	85.3	91.5
HTR2B (h) (agonist radioligand)	108.6	94.9	101.8
HTR3A (h) (antagonist radioligand)	97.2	99.9	98.5
NR3C1 (h) (agonist radioligand)	99.3	107.6	103.4
AR (h) (agonist radioligand)	96.4	100.1	98.2
AVPR1A (h) (agonist radioligand)	111.1	104.7	107.9
Ca2+ channel (L dihydropyridine site; rat cerebral cortex) (antagonist radioligand)	100.2	99.1	99.6
KCNH2 hERG (human)- [^{3}H] Dofetilide binding site)	106.2	106.2	106.2
KV channel ([^{125}I]α-dendrotoxin binding site, antagonist radioligand, rat cerebral cortex)	100	107.4	103.7
NET (h) (antagonist radioligand)	112.8	105.9	109.4
DAT1 (h) (antagonist radioligand)	102.4	99.3	100.8
SERT (h) (antagonist radioligand)	100.3	104.1	102.2

Assay<br>(Enzyme and cell-based assays; h: human)	1st replicate / % of Control	2nd replicate / % of Control	Mean / % of Control
LCK Human TK Kinase Enzymatic Radiometric Assay [Km ATP]	108.9	111.9	110.4
COX1 (h)	127.6	130.3	129
COX2 (h)	80.1	86.9	83.5
PDE3A (h)	102	107.6	104.8
PDE4D2 (h)	106.1	123.1	114.6
Acetylcholinesterase (h)	107.1	95.5	101.3

Table S1. Target profiling of CPD10. Related to Figure 5 and STAR Methods

Upper panel: Target profiling by radioligand competition assay was performed in the absence (Control) and presence of CPD10 (10 μM).

Lower panel: Target profiling by enzyme and cell-based assays in the absence (Control) and presence of CPD10 (10 μM).

The table shows two individual replicates of each assay in the presence of CPD10, and their means represented as % of control values, which were set to 100 %.

Gene name	Affymetrix Probe set ID	AD+C10-1	AD+C10-2	AD-1	AD-2
NeuN (Hrnbp3; Rbfox3)	1427286_at	949.4	911.9	780.7	818.6
NeuN (Hrnbp3; Rbfox3)	1456149_at	1747.9	1720.6	1400.5	1422.9
Gfap	1426509_s_at	97.9	97.5	125.1	124.4
Gfap	1440142_s_at	467.5	414.5	854.5	897.5
Cx3cr1	1450020_at	752.8	717.6	507	402
Cx3cr1	1450019_at	44	43.4	25	19.1

Table S2. Heat map data of whole genome microarray gene expression profiling of CPD10 treatment effects in frontal cortices of Tg2576 AD mice. Related to Figure 6K.

Whole transcriptome microarray gene expression profiling was performed to detect CPD10 treatment effects in frontal cortices of 18-month-old, male Tg2576 mice. The heat map of Figure 6K shows selected, AD-related genes, with were significantly altered ($p<0.05$; unpaired, two-tailed t-test) in frontal cortex specimens of treated Tg2576 mice (AD+C10) compared to untreated control Tg2576 mice (AD). Table S2 lists gene names, probe set IDs and probe set intensities of genes shown in Figure 6K. Probe sets detect neurons (*NeuN*), the AD-related gliosis response of astrocytes (*Gfap*), and Aβ-clearing microglia (*Cx3cr1*). Results are from n = 2 GeneChips (Affymetrix GeneChip Mouse Genome MG430 2.0 Array) with cRNAs from n=3 mice pooled for one GeneChip (total n = 6 mice per study group). P-values were determined by the unpaired, two-tailed t-test for comparisons of probe set intensities of treated AD mice (AD+C10-1, AD+C10-2) vs. untreated AD controls (AD-1, AD-2): p=0.039, 0.003, 0.0002, 0.0061, 0.0368, 0.0182 (df = 2; t = 4.914, 18.27, 67.10, 12.75, 5.069, 7.301).

Group-1	Gender	Age (years)	AD symptoms
Patient1*	female	60	yes
Patient2*	female	67	
Patient3	female	67	
Patient4	female	67	
Patient5	female	67	
Patient6	female	57	
Patient7	male	68	
Patient8*	female	67	yes
Patient9	female	68	
Patient10*	female	58	
**Group-2**	**Gender**	**Age (years)**	**AD symptoms**
Patient11	male	58	
Patient12	female	73	
Patient13*	female	72	
Patient14	female	63	
Patient15	male	45	
Patient16*	female	72	yes
Patient17	female	58	
Patient18	female	40	
Patient19	female	43	
Patient20	female	51	
Patient21	female	56	
Patient22	female	38	
Patient23*	male	68	
Patient24	female	67	
Patient25	female	74	
Patient26	male	51	
**Group-3**	**Gender**	**Age (years)**	**AD symptoms**
Patient27	female	55	
Patient28	female	61	
Patient29	female	50	
Patient30*	male	66	yes
Patient31	female	67	
Patient32	female	56	
Patient33	male	56	
Patient34	female	48	
Patient35	female	46	
Patient36	female	51	
Patient37	female	62	
Patient38	female	55	

Table S3. Characteristics of human patients. Related to STAR Methods and Key Resources Table.

The study performed whole genome microarray gene expression profiling of cerebral cortex specimens from 38 human patients. Cerebral cortex biopsy specimens were taken in frame of surgery for glioblastoma. Four patients had symptoms of AD and dementia. The four patients with dementia likely due to AD, and the four age- and gender-matched patients without dementia, which were used as controls for protein studies, are marked with an asterisk.

Table S4. Oligonucleotides. Related to Key Resources Table.

CosSHa-forward
5`-GCT TCA GCC TGC GTG CTG GAC AAT GAC GTG-3`

TOMM6-reverse
5`-CCT GGC TGA GGT GCC ATG AGG TCA ATG TCA C-3`

Prp-forward
5`-AAG CGG CCA AAG CCT GGA GGG TGG AAC A-3`

APP-forward
5`-CTG ACC ACT CGA CCA GGT TCT GGG T-3`

Prp-reverse
5`-GTG GAT AAC CCC TCC CCC AGC CTA GAC CA-3`

GRK2-forward
5`-GCC TGC CCA TGG AGG AGA TCC AGT CGG-3`

GRKInh-forward
5 - ATG GCC AAG TTC GAG CGC CTG CAG ACC GTG - 3`

Sp6-reverse
5`-TAG AAG GCA CAG TCG AGG CTG ATC AGC GAG-3`

CMV-forward
5`-CGC AAA TGG GCG GTA GGC GTG-3

ARRB1-reverse
5`-GGT CAG GCC CAG GAC ATC CAG GTC CTC CCG-3`

TAU-forward
5`-GAC CAA GAG GGT GAC ACG GAC GC-3`

TAU-reverse
5`-TGC CGC CTC CCG GGA CGT GTT TG-3`
